# Resuscitation Leadership Training: A Simulation Curriculum for Emergency Medicine Residents

**DOI:** 10.15766/mep_2374-8265.11278

**Published:** 2022-10-11

**Authors:** Rachel Gartland, Lauren Conlon, Scott Livingston, Joshua E. Glick, Gillian Bach, Michael E. Abboud

**Affiliations:** 1 Instructor of Clinical Emergency Medicine, Department of Emergency Medicine, University of Rochester Medical Center; 2 Associate Professor, Department of Emergency Medicine, Perelman School of Medicine at the University of Pennsylvania; 3 Second-Year Resident, Department of Emergency Medicine, Hospital of the University of Pennsylvania; 4 Assistant Professor, Department of Emergency Medicine, Perelman School of Medicine at the University of Pennsylvania; 5 First-Year Fellow, Department of Emergency Medicine, Hospital of the University of Pennsylvania

**Keywords:** Resuscitation, Communication Skills, Emergency Medicine, Leadership Development/Skills, Simulation

## Abstract

**Introduction:**

Throughout training, emergency medicine (EM) residents must learn to work within, and eventually lead, multidisciplinary teams in high-acuity dynamic situations. Most residents do not undergo formal resuscitation team leadership training but learn these skills through mentorship by and observation of senior physicians. We designed and implemented a formal simulation-based leadership training program for EM residents.

**Methods:**

We developed a resuscitation team leadership curriculum in which 24 junior EM residents participated in an initial simulation of a critically ill patient before undergoing a didactic presentation regarding crisis resource management (CRM) principles. Residents applied those principles in three subsequent simulations. Faculty observers evaluated each case using EM Milestones, the Ottawa Global Rating Scale (GRS), and critical actions checklists. Residents then completed surveys evaluating their own leadership and communication skills before and after the course.

**Results:**

Scores from the Ottawa GRS, critical actions checklists, and several of the EM Milestones were significantly better in the latter three cases (after completing the CRM didactics) than in the first case. After completing this curriculum, residents felt that their ability to both lead resuscitations and communicate effectively with their team improved.

**Discussion:**

Implementation of the resuscitation team leadership curriculum improved EM residents’ leadership performance in critically ill patient scenarios. The curriculum also improved residents’ comfort in leading and communicating with a team. Similar formal leadership development curricula, especially when combined with simulation, may enhance EM physician training. Future studies will include other multidisciplinary team members to create a more realistic and inclusive learning environment.

## Educational Objectives

By the end of this curriculum, learners will be able to:

1. Identify leadership principles as described in crisis resource management (CRM) and TeamSTEPPS.
2. Apply leadership principles as described in CRM and TeamSTEPPS while leading a team in high-fidelity simulations of critically ill patient scenarios.
3. Exhibit increased competence in their communication and resuscitation team leadership skills.

## Introduction

Emergency medicine (EM) resident physicians not only must develop extensive medical knowledge and technical skills to care for a vast array of patients but also must learn to lead a diverse medical team in critical patient situations. This ability to lead and communicate effectively with a variety of team members in dynamic, high-stress situations is expected of graduating EM residents. Nevertheless, most residents do not undergo formal leadership training, instead learning these skills by observing more senior physicians before being thrust into the team leader role themselves. While some programs and specialties have implemented a dedicated leadership development curriculum, the practice is not widespread.^1^

Frameworks that focus on leadership skills, particularly in times of crisis or high-stress situations, are commonly taught in nonmedical fields and can be adapted to medical training. Crisis resource management (CRM), originally used for aviation, is one such paradigm that teaches the clear allocation of responsibilities, effective communication, situational awareness, and resource utilization.^2–5^ Likewise, the Department of Health and Human Services has collaborated with other health care experts to create programs like TeamSTEPPS, a tool kit with standardized printed and video resources that emphasize team skills by highlighting four keys to CRM: leadership, communication, situation monitoring, and mutual support.^6^

As CRM began to become part of health care delivery, scales like the Ottawa CRM Global Rating Scale (Ottawa GRS) were subsequently created to evaluate CRM skills specific to medicine.^7,8^ The Ottawa GRS rates learners on a 7-point scale in the categories of overall performance, leadership skills, problem-solving skills, situational awareness skills, resource utilization skills, and communication skills and has been used to reliably evaluate CRM performance in simulations.^9,10^

Prior research has shown that leadership training using these frameworks can improve a team's care of critical patients,^11^ is valued by learners,^4^ is beneficial to providers of all skill levels,^12^ and leads to improved communication.^13^ Other groups have specifically combined CRM-based didactics with simulation to improve leadership skills, which can be measured using CRM-based milestones and the Ottawa GRS.^3,4,6,7,14,15^ The simulated setting offers a practical and realistic way for junior residents in particular to practice and improve their skills before becoming senior residents leading real-life resuscitation teams.

The ACGME instituted the EM Milestones in 2012 to provide competency-based benchmarks for trainees in EM. These Milestones allow residency programs to track progression of their residents from intern year through graduation, focusing on over 20 areas measured by observers.^16,17^ Select Milestones have also been used to measure trainee performance in simulation.^18^ Likewise, critical actions checklists, which are a list of the most important interventions in a simulated case, have been found useful in evaluating resident performance in simulated resuscitations, with a high degree of interrater reliability.^19^ Combining CRM metrics, Milestones, and critical actions checklists gives EM faculty several tools to evaluate resident effectiveness in leading medical resuscitation.

Our institution did not previously have a standardized leadership training curriculum for EM residents. We addressed this deficiency in leadership training by developing a curriculum called Resuscitation Leadership Training (RLT), which featured high-fidelity simulation, critical care scenarios, and CRM teaching to improve the leadership and communication skills of junior EM residents at our institution. We used the TeamSTEPPS framework to teach leadership and teamwork via a combination of didactic learning, simulation cases, and deliberate practice to hone these important skills. We intentionally created simulation cases that involved critically ill patients, as these would require the leading resident to coordinate with a larger medical team in highly dynamic situations. The goal of this curriculum was to facilitate the transition from junior to senior resident by improving resuscitation team leadership skills that are often difficult to teach but imperative to the growth of EM physicians.

While prior research has similarly measured trainee improvement after CRM-based teaching and simulation using leadership metrics such as the Ottawa GRS, we developed a curriculum in which we could also quantify and trend trainee performance in the medical management of cases using the EM Milestones and critical actions checklists. We hypothesized that participation would specifically improve residents’ scores in CRM metrics as measured by select EM Milestones and the Ottawa GRS, which would in turn improve their medical management of critically ill patients. We also anticipated that residents would have increased comfort in leading resuscitations and their ability to communicate effectively with their team after completing this curriculum. Other curricula in *MedEdPORTAL* and elsewhere have sought to teach similar skills to trainees (within fields including EM and trauma),^20,21^ but our curriculum is unique in its novel didactics and also given the extensive evaluation of the efficacy of its educational materials on simulated patient care and team performance.

## Methods

### Development

For this curriculum, we created four simulation cases, each based on cardiac resuscitation in critically ill patient scenarios (Appendices A–H) and lasting 15–20 minutes. Each case included 10 critical actions that participants were expected to complete during the case (Appendices I–L). The simulation mannequin was controlled by a trained senior resident with extensive knowledge of each case. Each case was designed to address specific high-yield teaching points in medical management; in particular, the cases focused on the management of various types of hemodynamic instability due to a cardiac emergency.

The curriculum was run seven separate times in 4-hour sessions over 2 years, involving PGY 2 residents across 2 separate years within our EM residency. Prior to the implementation of the curriculum, we did a trial run with the class a year ahead of the inaugural PGY 2 group in which we ran through each case, discussed possible management decisions, anticipated errors, and made small modifications to minimize the risk of unanticipated medical decision-making.

### Equipment/Environment

The curriculum was completed at the Penn Medicine Rittenhouse Simulation Center. This complex is housed within a building that was previously a hospital, using old operating suites that have been retrofitted into simulation rooms meant to mimic hospital care rooms, equipped with a fully functional high-fidelity simulation mannequin, monitors, moulage, and a full array of resuscitation equipment and imitation medications.

### Personnel

Junior EM residents (end of PGY 2 year) from our institution participated in the RLT program. The residents’ time was considered equivalent to on-shift time and was approved by the program director. All participants gave informed consent prior to starting the first session. The curriculum received institutional review board exemption.

Two trained faculty members were present at all times to observe and grade each case. One senior resident, who had been trained in the use of the simulation mannequins, operated the mannequin and monitors during each case.

### Implementation

EM residents at our institution completed this curriculum in groups of three or four. The residents in each group took turns leading the simulation cases while the others were assigned supporting roles including history taking, airway management, and pharmacy. Faculty were on-site with a tablet or computer with large display for showing all case media, including imaging, EKGs, and relevant lab results. After the first simulation case, residents were given a standardized didactic session discussing CRM and resuscitation team leadership. The didactic presentation (Appendix M) used TeamSTEPPS concepts to discuss techniques to help learners become competent leaders with good teamwork and communication skills; in particular, we stressed leading from the foot of the bed, practicing closed-loop communication, frequent summarization (sharing the mental model), and appropriate utilization of resources. Following the discussion, the residents took turns completing the final three patient scenarios in which they applied and solidified the concepts from the didactics.

### Debriefing

The groups debriefed after each simulation case. Each debrief was led by two faculty members and one senior resident who were present during all the cases. The residents were asked to identify parts of the case that were done well or could have been done better, regarding not only the medical management of the case but also its leadership, communication, and teamwork aspects. The faculty members then led a brief discussion about their own observations regarding the teamwork and leadership of the resident group during the simulation and the application of CRM and TeamSTEPPS principles. The faculty members also reviewed the medical management and teaching pearls for each case as listed in the debrief teaching pearls handout (Appendix N). The goal of the debrief was to discuss and further solidify the knowledge and skills practiced in each simulation case.

### Assessment

Two trained faculty members observed and independently graded each case using select EM Milestones (Appendix O),^16,17^ the Ottawa GRS (Appendix P),^9^ and the critical actions checklists (Appendices I–L). Prior to the curriculum's onset, all the faculty members read through the Ottawa GRS references within this resource and attended a meeting where the use of the scoring systems was discussed to ensure consistency amongst evaluators.

The scores from the two faculty for each category were averaged to give a final score. Milestones were graded from 1 to 5, the Ottawa GRS graded from 1 to 7, and checklists graded as yes (1) or no (0) for each critical action. The resident participants at the end of each session were asked to complete a survey (Appendix Q) to assess their comfort levels on a 7-point scale (1 = *not at all ready,* 7 = *very ready*) in leadership, communication, and medical management skills. Residents were also asked to describe the curriculum qualitatively, and coding was used to categorize answers.

The scores from the EM Milestones, Ottawa GRS, critical actions checklists, and self-evaluation survey results were compiled, and the mean, standard error, standard deviation, and 95% confidence interval were calculated for each category. A paired *t* test was performed to determine significance at a two-sided alpha of .05.

## Results

Twenty-four residents—11 during the first year and 13 in the second year—have participated in this curriculum over the 2 years that it has been implemented.

**Figure 1.**
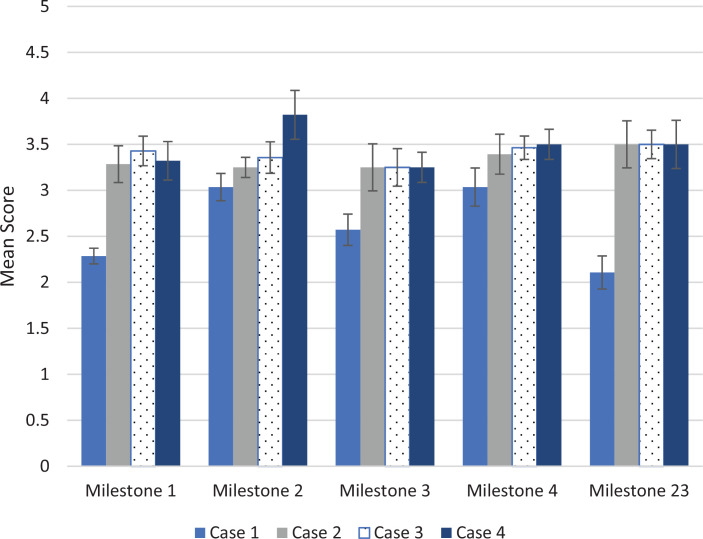
Emergency medicine Milestone score results. Mean scores and standard errors (error bars) for five emergency medicine Milestones for each of the four cases are shown. Each Milestone was scored on a 5-point scale, with 5 being the highest score possible. The *p* values for cases 2, 3, and 4 as compared to case 1 were less than .05 for Milestones 1, 3, and 23.

After the didactic presentation following case 1, residents’ ratings for Milestones 1 (Emergency Stabilization), 3 (Diagnostic Studies), and 23 (Team Management) were significantly higher for cases 2, 3, and 4 compared to case 1 (*p*s < .05). The ratings for Milestone 2 (Performance of Focused History and Physical Exam) and 4 (Diagnosis) were not significantly increased but trended towards higher scores in the latter three cases. Furthermore, while not statistically significant, the ratings for Milestones 2 and 4 trended towards higher scores in each subsequent simulation case (Figure 1).

**Figure 2.**
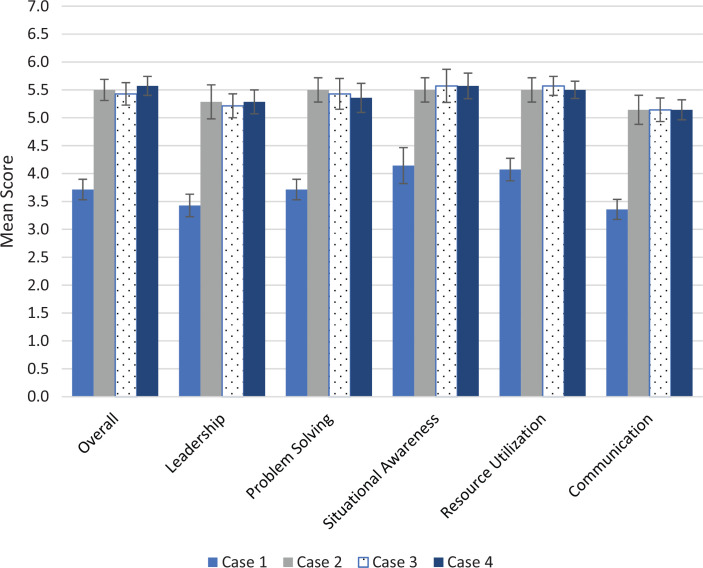
Ottawa Global Rating Scale score results. Mean scores and standard errors (error bars) for each category of the Ottawa scale for each of the four cases are shown. Each category was scored on a 7-point scale, with 7 being the highest score possible. The *p* values of scores for cases 2, 3, and 4 as compared to case 1 for each category were all less than .05.

**Figure 3.**
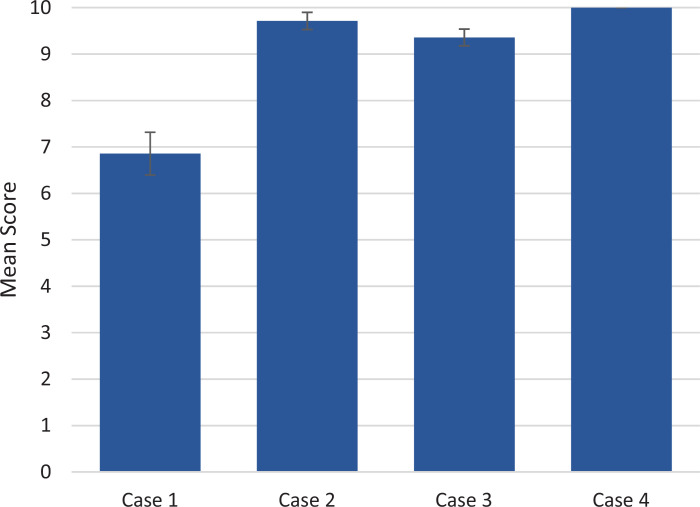
Critical actions checklist results. Ten critical actions essential to proper medical management were predetermined for each case. The mean numbers of completed critical actions and standard errors (error bars) are shown for each case. The *p* values of scores for cases 2, 3, and 4 as compared to case 1 were all less than .05.

Residents also scored significantly higher on all categories of the Ottawa GRS in cases 2, 3, and 4 than in the first case (Figure 2; all *p*s < .05). Each case had 10 critical actions that were essential to proper management of the patient; residents’ scores on the critical actions checklists were also significantly higher on cases 2,3, and 4 after completing the CRM didactics than on case 1, and although not statistically significant, scores trended higher in case 4 than in case 2 (mean for case 1: 6.9 vs. case 2: 9.7, case 3: 9.4, case 4: 10.0; *p* < .05; Figure 3). Formal interrater reliability statistical analysis of the faculty evaluator scores was not used, but all scores for each metric were within 1 point for all scenarios.

**Figure 4.**
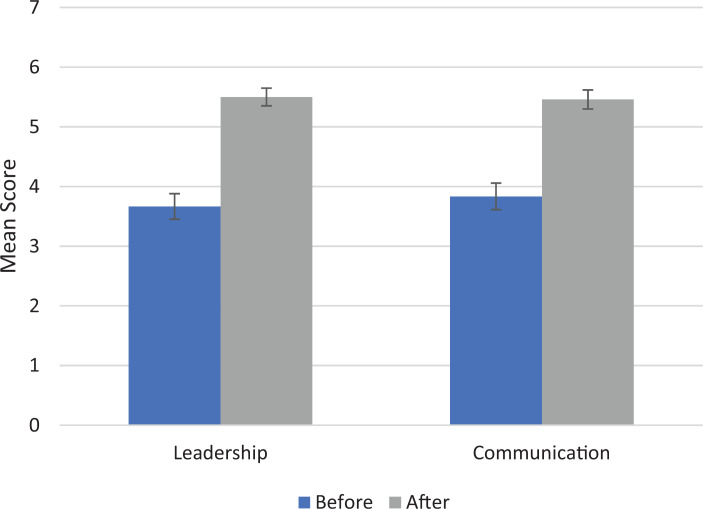
Leadership and communication survey results. Residents were asked at the end of day 1 to rate how ready they felt to lead a resuscitation before and after completing Resuscitation Leadership Training (1 = *not at all ready*, 7 = *very ready*); the mean scores and standard errors (error bars) are shown under Leadership. Residents were also asked after day 1 to rate how effective their communication skills were before and after completing Resuscitation Leadership Training (1 = *not at all effective*, 7 = *very effective*); the mean scores and standard errors (error bars) are shown under Communication. All *p* values were less than .01.

After completion of the RLT simulation curriculum, survey results revealed that residents felt significantly more ready to lead a resuscitation (scale: 1–7; mean before course: 3.7 vs. after course: 5.5, *p* < .001) and believed that their communication skills during resuscitations were significantly more effective (mean before course: 3.8 vs. after course: 5.5, *p* < .01; Figure 4).

Qualitative feedback for the RLT curriculum was overwhelmingly positive. Residents described the curriculum as “high yield” and “helpful” and felt it was an important addition to their residency training. Multiple residents suggested expanding the training to include more types of cases, such as trauma cases, to address other areas of need.

## Discussion

The goal of this curriculum was to improve junior EM residents’ effectiveness and comfort in leadership roles during resuscitations by providing formal, structured, CRM-based teaching and practice via simulation. We found that after attending didactic presentations about CRM and TeamSTEPPS and how they might be applied to EM, residents scored higher on several measures of leadership skills. Residents also completed more critical actions in each of the cases after the didactics than in the first case, suggesting that their medical management was also enhanced by learning these leadership frameworks and applying good communication and team skills. Scores in subsequent simulations trended towards improved performance in several of our measures of success, suggesting that repeated application of these new skills in simulation might be beneficial. Future investigations will be helpful to evaluate this improvement with repeated practice. We found that after completion of the RLT curriculum, EM residents also felt better prepared to be successful leaders and to communicate effectively with their team when caring for critical patients.

Because we modified cases to ensure clarity after our trial run of the curriculum, we did not encounter any major challenges during the implementation. Cases were effective in getting residents to work together and identify medical diagnoses and treatments but did not result in any major unanticipated medical decisions by the resident teams. However, we recommend that any institution adopting a program like this one also do a trial run of the cases, since, because team structure and medical management can vary across different institutions, challenges may arise when the protocol is implemented with a new group of learners. Due to schedule constraints, multiple faculty members participated in different sessions. Additionally, faculty ratings using the Ottawa GRS and EM Milestones could have been influenced by personal bias. Although we trained each faculty member on the scoring systems in the same manner, having the same faculty present at every session may lend more consistency to the data and evaluations.

Our data have several limitations. While the use of simulation mannequins allowed residents to practice in a realistic yet controlled manner, there were clear limitations to using simulation rather than actual clinical scenarios. Beyond the use of a mannequin as opposed to a real patient, this simulation curriculum was limited to residents only. As we further establish the curriculum and expand its footprint, we hope to include nurses, ancillary staff, and others both to create a more realistic simulated scenario and to teach CRM skills to more members of the emergency department team. Additionally, the sample size was inherently limited by the number of EM residents per year at our institution. Applying the curriculum over several years of training or with larger populations (including expanding beyond a single site) will improve the power and generalizability of our data.

Some of our measurements of success (several EM Milestones and the critical actions checklists) are based on outcomes reached by the resuscitation team as whole. However, the assumption that a team is successful because it has a strong or effective leader is not always true; weaker team leaders may be supplemented by stronger teammates. Strong team members, if also using shared mental models and open communication during resuscitations, may falsely elevate scores for a team leader. Future studies could use other assessment tools to more directly evaluate the skills of the team leader independent of the rest of the team.

While our resident survey results suggest that learners felt more confident leading and communicating during resuscitations, these data rely on self-report in a single postcurriculum survey and are therefore prone to bias. After completing the simulations, learners reported their feelings both from before and after the curriculum. We considered a pre- and postcurriculum survey design to increase the strength of the data but felt that a precurriculum survey would tip the residents off to the nature of the cases in which they were about to participate. As a result, we have likely introduced recall bias that skews our pretest data. For the sake of reporting and analyzing data, minimizing recall bias by having separate pre- and postcurriculum surveys may be more helpful but could detract from the in situ educational value for residents. It is also possible that residents’ increased confidence was confounded by increased comfort in managing these particular critically ill patient scenarios after review of the medical management for each case in the debrief.

Future research could also look at the effect that the curriculum's implementation has on development of residents’ resuscitation leadership skills compared to residents of previous years (or from other institutions) who did not receive formal CRM training. As we expand this curriculum to involve more residents and other members of our interdisciplinary team, we hope to measure the ability of participants to apply the skills they have learned in simulation to improve care for real emergency department patients.

## Conclusions

Our data suggest that didactics in CRM, when combined with subsequent application of skills in simulation, may improve EM residents’ resuscitation leadership skills, medical management, and abilities to lead and effectively communicate during resuscitations. As the care of critically ill patients involves many team members in dynamic and stressful situations, the ability to effectively lead is vital to safely caring for patients. We hope that this curriculum will help create strong emergency physician leaders and eventually can be applied more broadly across both the University of Pennsylvania and other medical training programs throughout the country.

## Appendices


Sim Case - STEMI and VFib Arrest.docxCase Media and Labs - STEMI and VFib Arrest.pptxSim Case - Massive Pulmonary Embolism.docxCase Media and Labs - Massive PE.pptxSim Case - Wide Complex Tachycardia.docxCase Media and Labs - WCT.pptxSim Case - Missed Dialysis.docxCase Media and Labs - Missed Dialysis.pptxCAC - STEMI and VFib Arrest.docxCAC - Massive Pulmonary Embolism.docxCAC - Wide Complex Tachycardia.docxCAC - Missed Dialysis.docxCRM Presentation.pptxDebrief Handout.pdfSelect ACGME EM Milestones List.pptxOttawa GRS.docxResident Survey.docx
*All appendices are peer reviewed as integral parts of the Original Publication.*
