# Supplementary material for: Resuscitation Leadership Training: A Simulation Curriculum for Emergency Medicine Residents
Source: MedEdPORTAL. 2022 Oct 11;18:11278. doi: 10.15766/mep_2374-8265.11278 (PMC9550795; doi:10.15766/mep_2374-8265.11278)
Supplement: Supplementary file 1 — Sim Case - STEMI and VFib Arrest.docxCase Media and Labs - STEMI and VFib Arrest.pptxSim Case - Massive Pulmonary Embolism.docxCase Media and Labs - Massive PE.pptxSim Case - Wide Complex Tachycardia.docxCase Media and Labs - WCT.pptxSim Case - Missed Dialysis.docxCase Media and Labs - Missed Dialysis.pptxCAC - STEMI and VFib Arrest.docxCAC - Massive Pulmonary Embolism.docxCAC - Wide Complex Tachycardia.docxCAC - Missed Dialysis.docxCRM Presentation.pptxDebrief Handout.pdfSelect ACGME EM Milestones List.pptxOttawa GRS.docxResident Survey.docx [file mep_2374-8265.11278-s001.zip › B. Case Media and Labs - STEMI and VFib Arrest.pptx]

## Slide 1
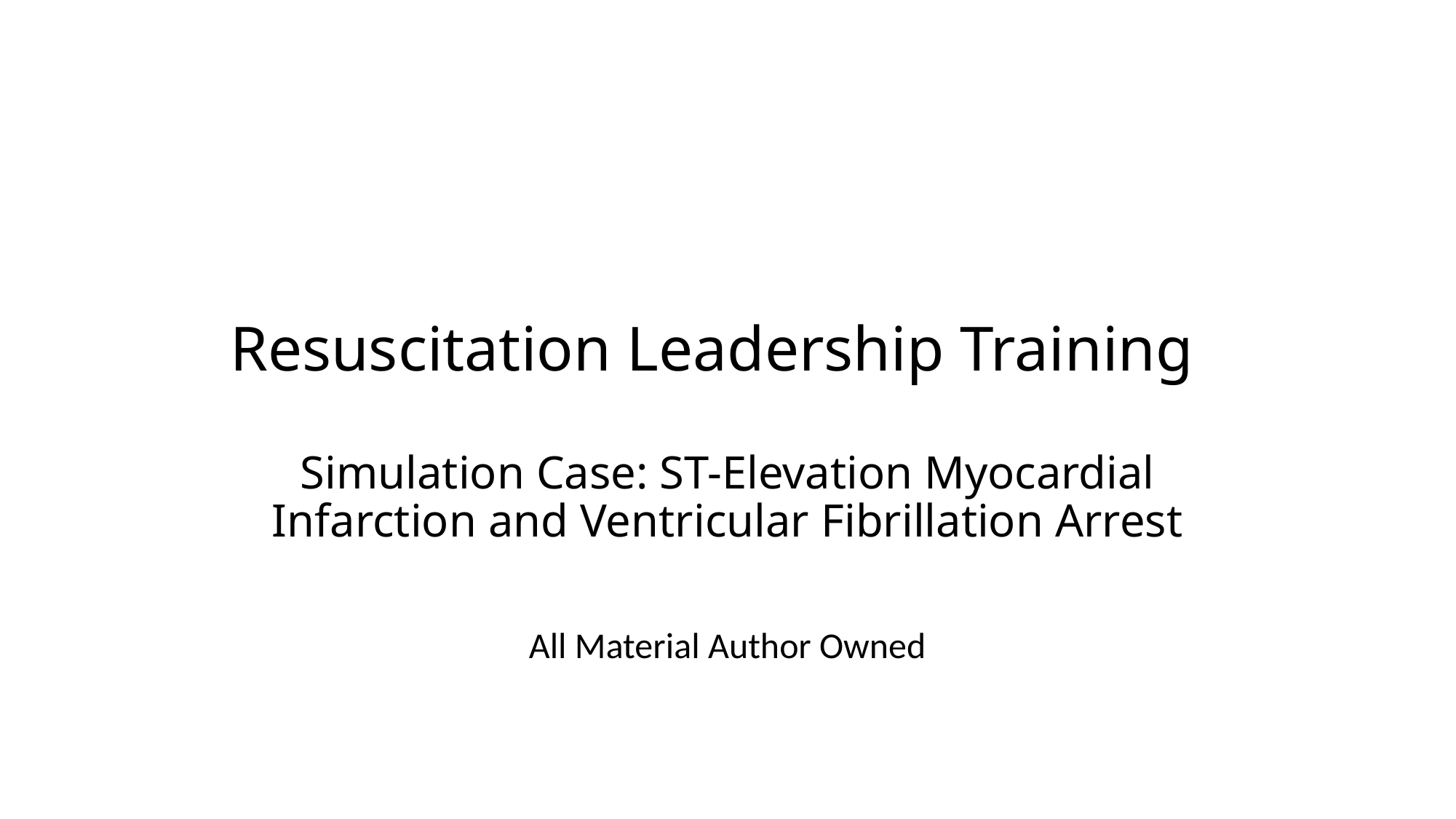

# Resuscitation Leadership Training Simulation Case: ST-Elevation Myocardial Infarction and Ventricular Fibrillation Arrest
All Material Author Owned

## Slide 2
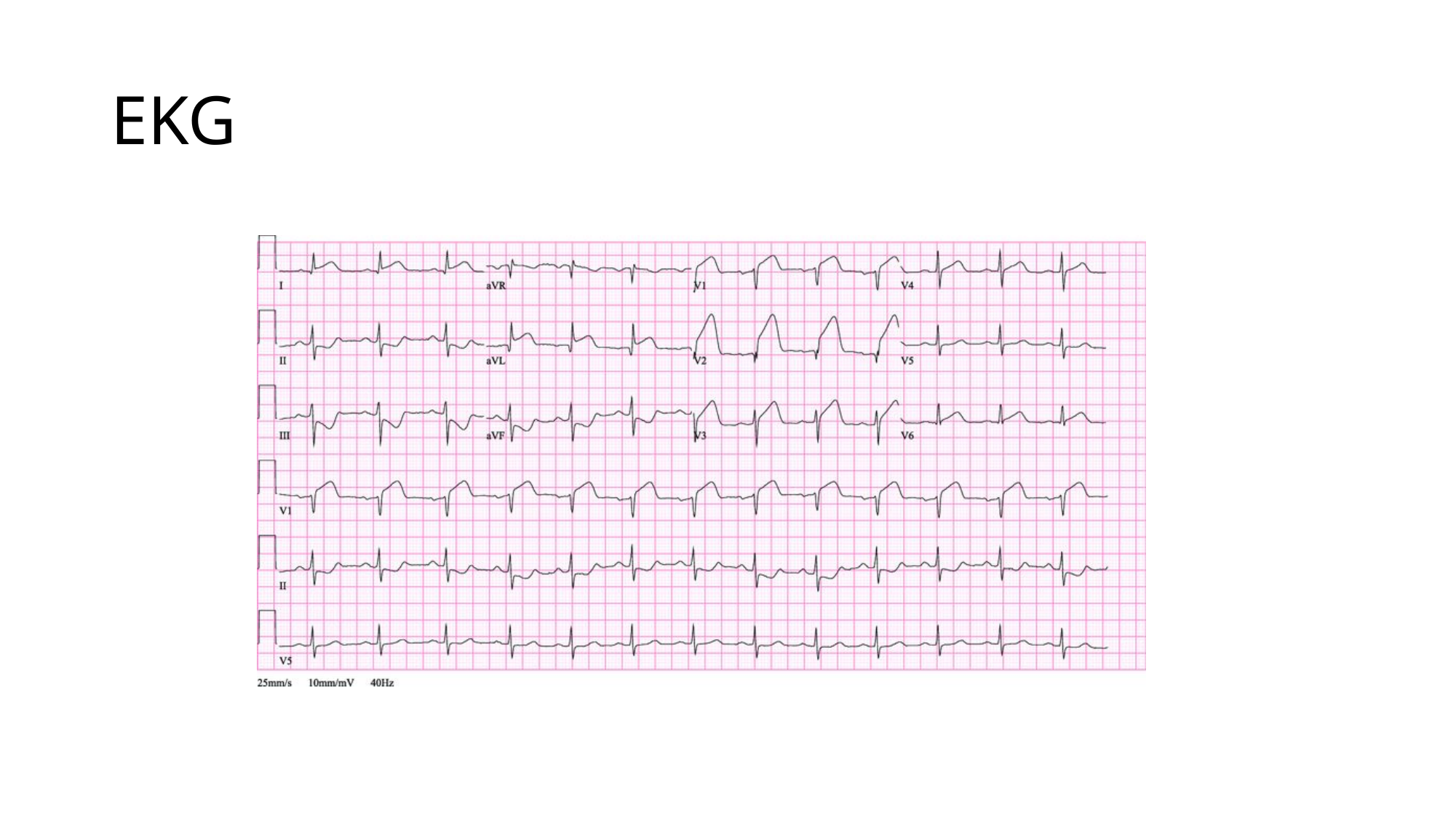

# EKG

## Slide 3
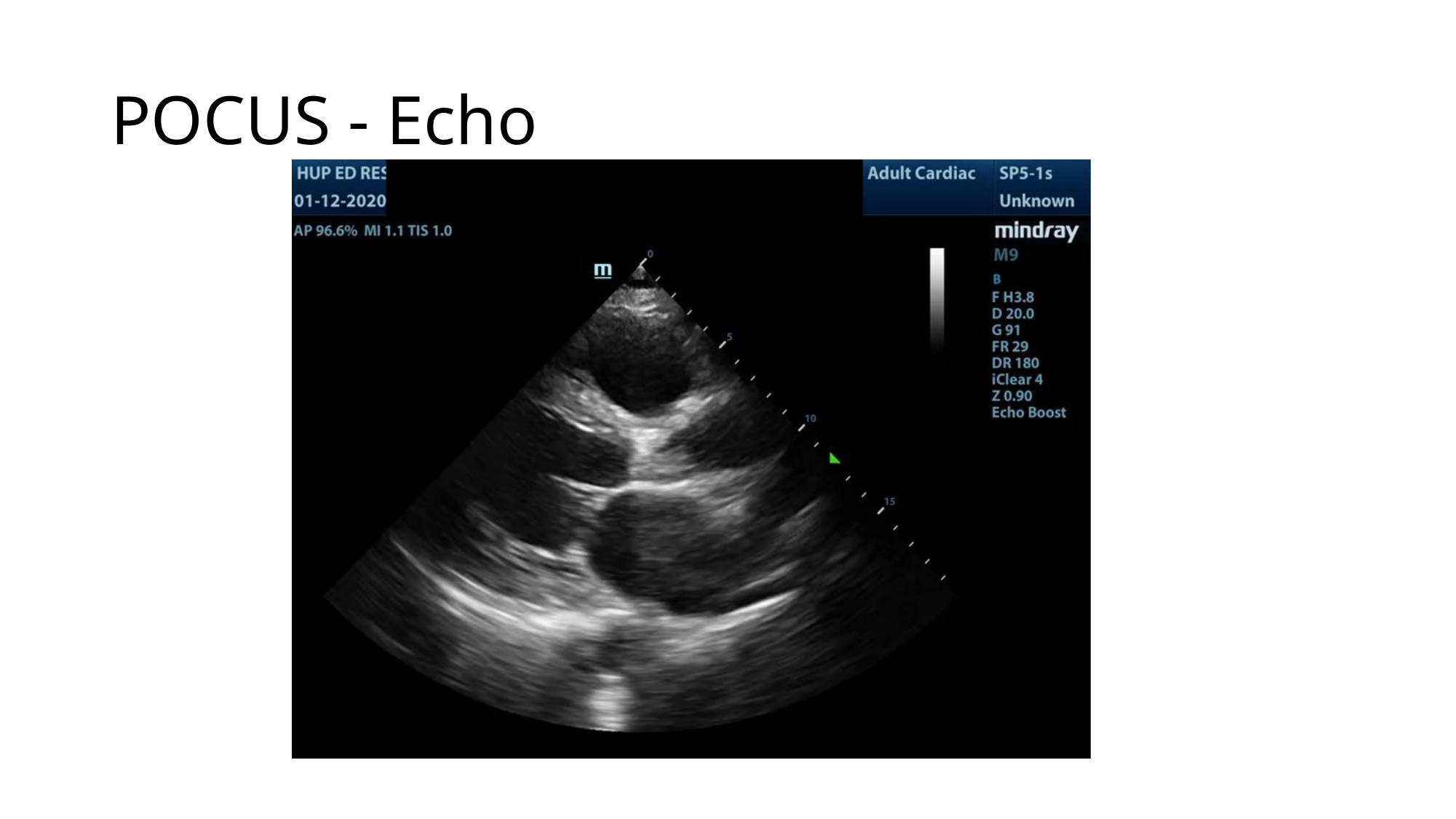

# POCUS - Echo

## Slide 4
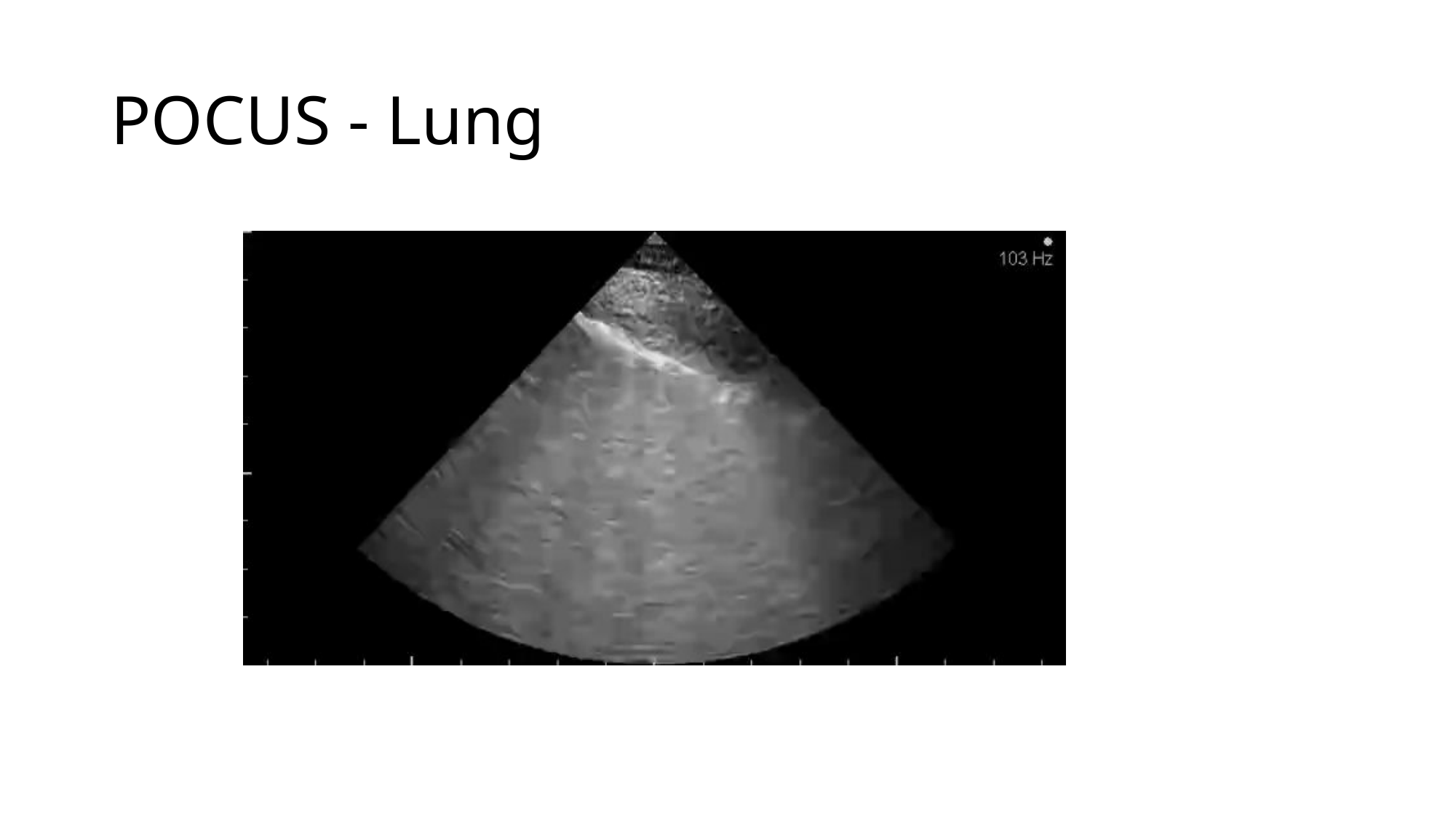

# POCUS - Lung

## Slide 5
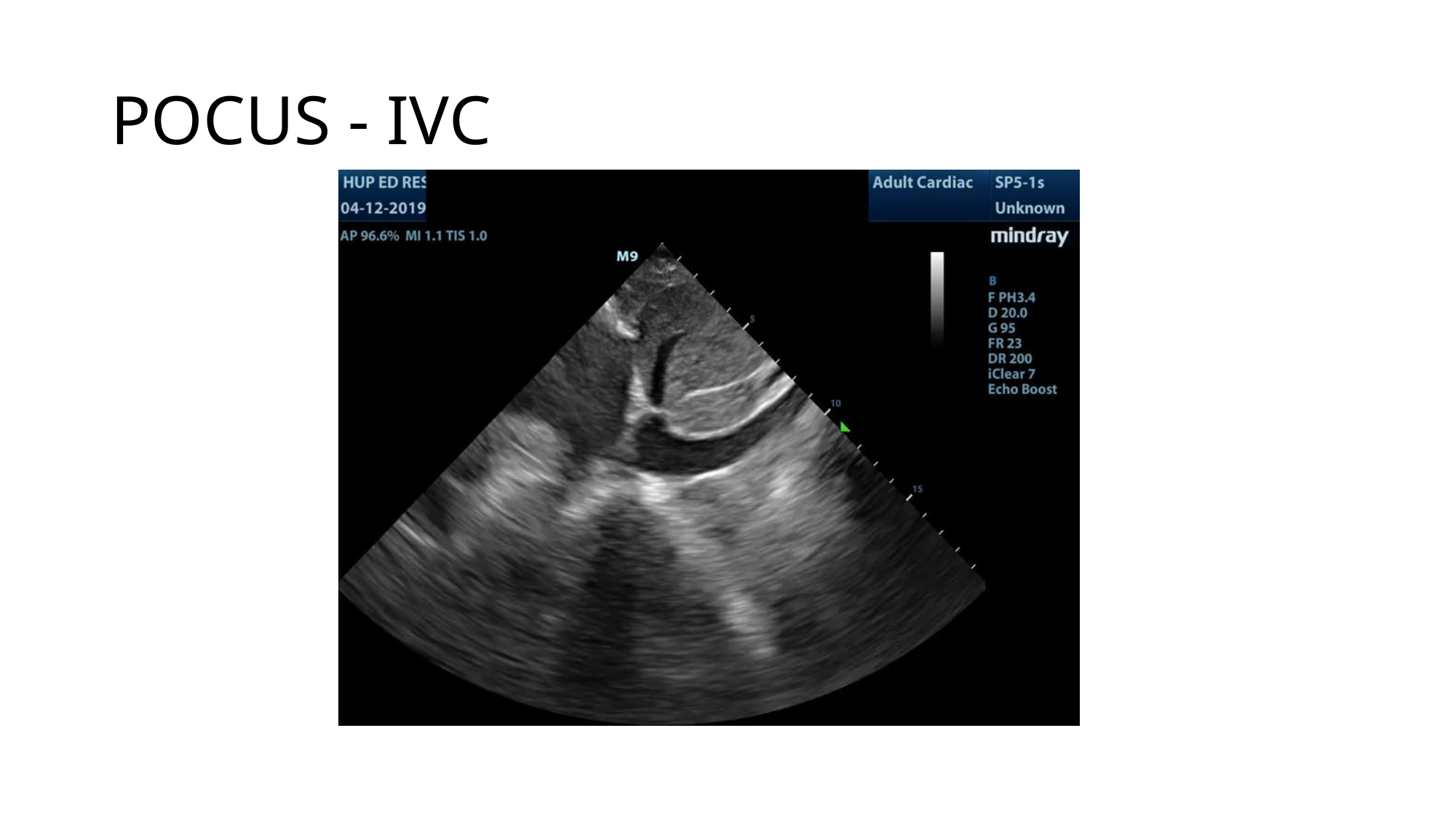

# POCUS - IVC

## Slide 6
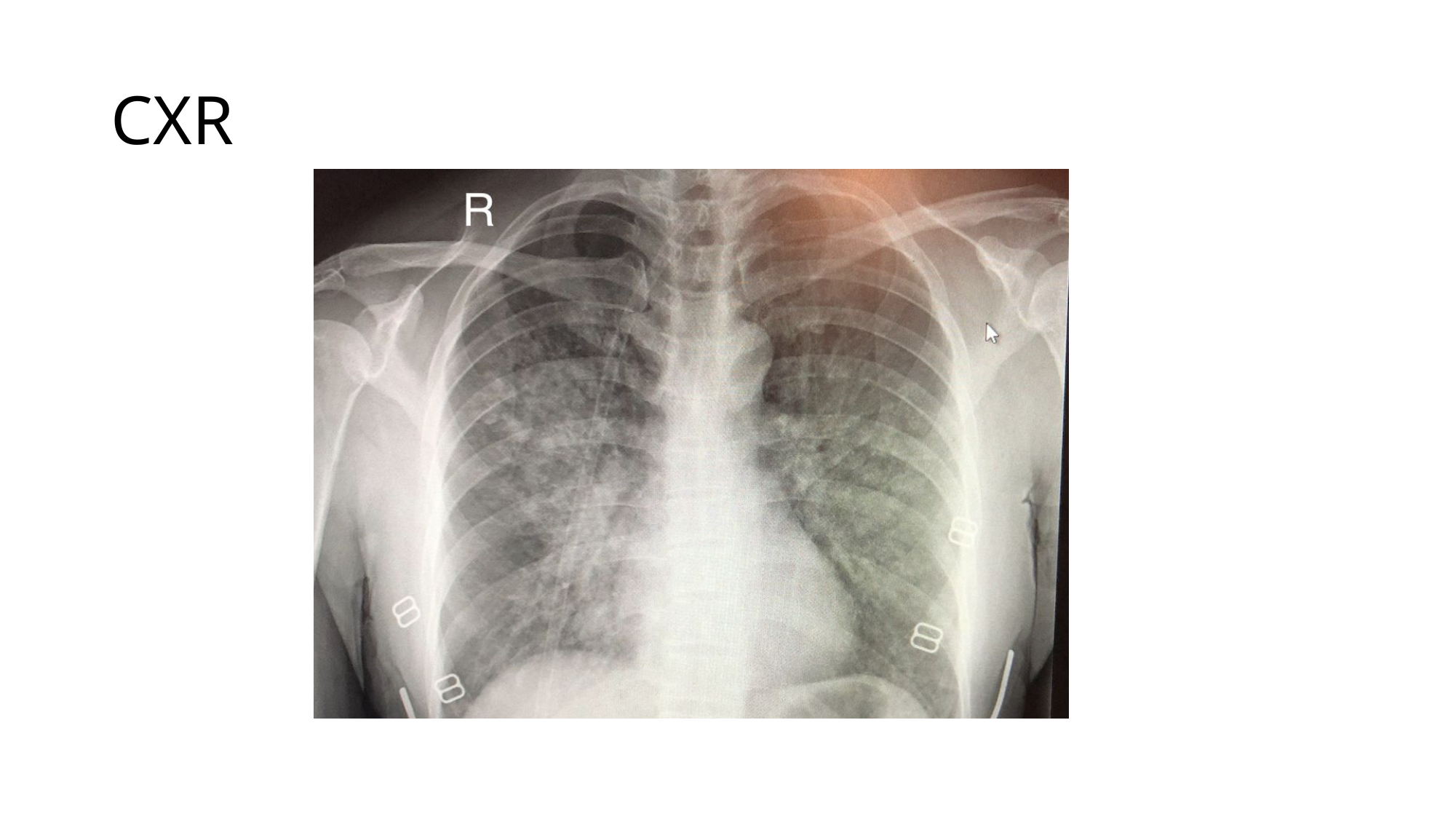

# CXR

## Slide 7
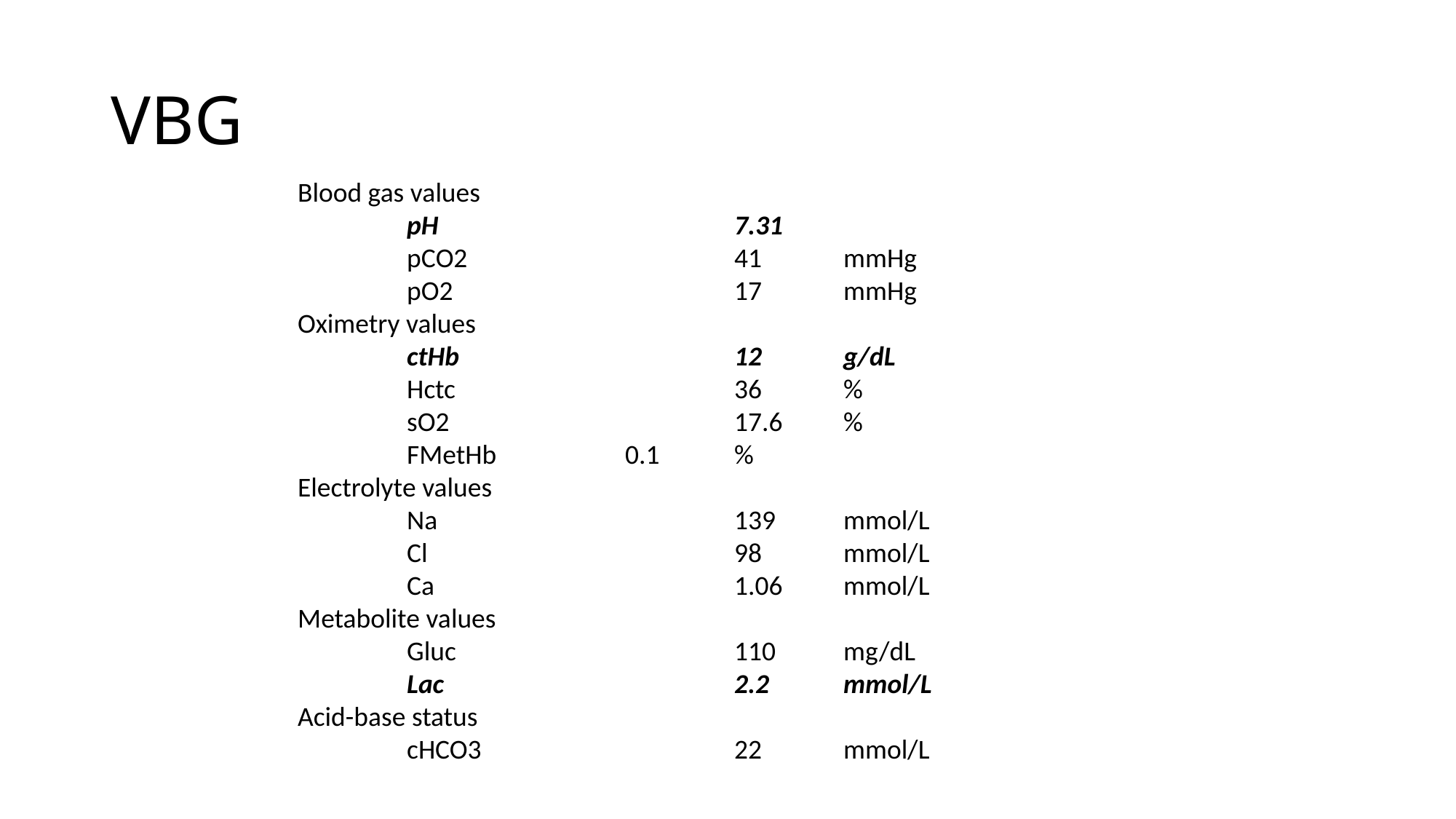

# VBG
Blood gas values
	pH			7.31
	pCO2			41	mmHg
	pO2			17	mmHg
Oximetry values
	ctHb			12	g/dL
	Hctc			36	%
	sO2			17.6	%
	FMetHb		0.1	%
Electrolyte values
	Na			139	mmol/L
	Cl			98	mmol/L
	Ca			1.06	mmol/L
Metabolite values
	Gluc			110	mg/dL
	Lac			2.2	mmol/L
Acid-base status
	cHCO3			22	mmol/L
