# Supplementary material for: Resuscitation Leadership Training: A Simulation Curriculum for Emergency Medicine Residents
Source: MedEdPORTAL. 2022 Oct 11;18:11278. doi: 10.15766/mep_2374-8265.11278 (PMC9550795; doi:10.15766/mep_2374-8265.11278)
Supplement: Supplementary file 1 — Sim Case - STEMI and VFib Arrest.docxCase Media and Labs - STEMI and VFib Arrest.pptxSim Case - Massive Pulmonary Embolism.docxCase Media and Labs - Massive PE.pptxSim Case - Wide Complex Tachycardia.docxCase Media and Labs - WCT.pptxSim Case - Missed Dialysis.docxCase Media and Labs - Missed Dialysis.pptxCAC - STEMI and VFib Arrest.docxCAC - Massive Pulmonary Embolism.docxCAC - Wide Complex Tachycardia.docxCAC - Missed Dialysis.docxCRM Presentation.pptxDebrief Handout.pdfSelect ACGME EM Milestones List.pptxOttawa GRS.docxResident Survey.docx [file mep_2374-8265.11278-s001.zip › A. Sim Case - STEMI and VFib Arrest.docx]

| **SIMULATION CASE TITLE:** ST-Elevation Myocardial Infarction and Ventricular Fibrillation Arrest  **AUTHORS:** Michael Abboud, MD, MSEd  **LEARNER AUDIENCE:** PGY-2 Emergency Medicine Residents | | | | | | | |  |
| --- | --- | --- | --- | --- | --- | --- | --- | --- |
| **PATIENT NAME:** John Smith  **PATIENT AGE:** 61 years old  **CHIEF COMPLAINT:** Chest pain  **PHYSICAL SETTING:** Emergency Department | | | | | | | |  |
|  | | | | | | | |  |
| **Brief narrative description of case** | | A 61-year-old male with a history of hypertension and diabetes presents with chest pain and is found to have ST elevations on EKG. Although initially awake and alert, he goes into ventricular fibrillation arrest during the case, requiring the participants to work together to effectively run ACLS, choose appropriate medications, and admit the patient to the cardiac catheterization lab. | | | | | |  |
| **Primary Learning Objectives** | | - Evaluate a patient with chest pain - Analyze the EKG and recognize ST-elevation myocardial infarction (STEMI) - Demonstrate knowledge of appropriate management of a patient with a STEMI - Demonstrate knowledge of appropriate management of ventricular fibrillation cardiac arrest (ACLS) - Apply team leadership and communication skills to direct the resuscitation of an unstable patient | | | | | |  |
| **Critical Actions** | | 1. Obtain an EKG 2. Place the patient on supplemental oxygen 3. Place defibrillation pads on the patient (before he arrests) 4. Give aspirin 5. Give a heparin bolus 6. Call the catheterization lab 7. Defibrillate at 200J when patient goes into ventricular fibrillation arrest 8. Intubate 9. Start an appropriate pressor 10. Give an appropriate antidysrhythmic medication | | | | | |  |
| **Learner Preparation or Prework** | | Learners should treat the mannequin and simulation as if it were a real patient scenario. | | | | | |  |
| Initial Presentation | | | | | | | |  |
| **Initial vital signs** | | BP 101/70, HR 88, T 98.9, RR 20, SpO2 97% on room air | | | | | |  |
| **Overall Setting and Appearance** | | The mannequin is lying in a stretcher in a hospital room. | | | | | |  |
| **Standardized Participants (and their roles in the room at case start**) | | None. | | | | | |  |
| **HPI** | | A 61-year-old male with history hypertension and diabetes presents with chest pain. He was in his usual state of health until 30 minutes prior to arrival when he was seated at work and developed left sided chest pain, pressure-like, now 10/10, constant, radiating to the jaw. He tried to get a drink of water but had worsening pain when he tried to walk to the water fountain. He has never had pain like this before. | | | | | |  |
| **Past Medical/Surgical History** | | **Medications** | | **Allergies** | | **Family History** | |  |
| Hypertension  Diabetes mellitus | | Amlodipine  Hydrochlorothiazide  Metformin | | Sulfa drugs | | Father had MI at age 59 | |  |
| **Physical Examination** | | | | | | | |  |
| **General** | | Distressed, diaphoretic, speaking full sentences | | | | | |  |
| **HEENT** | | PERRL, normocephalic/atraumatic, mucus membranes moist | | | | | |  |
| **Neck** | | Supple, no tracheal deviation. | | | | | |  |
| **Lungs** | | Clear to auscultation bilaterally, no respiratory distress | | | | | |  |
| **Cardiovascular** | | Regular rate and rhythm, no murmurs, +JVD | | | | | |  |
| **Abdomen** | | Soft, nontender, nondistended | | | | | |  |
| **Neurological** | | Alert, oriented x3, moving all extremities. Grossly non-focal neurologic exam. | | | | | |  |
| **Skin** | | Diaphoretic, warm/well-perfused, no edema | | | | | |  |
| **GU** | | Not done. | | | | | |  |
| **Psychiatric** | | Thought content normal, behavior appropriate. | | | | | |  |
| Instructor Notes - Changes and CASE Branch Points | | | | | | | | |
| **Intervention / Time point** | | | **Change in Case** | | **Additional Information** | | | |
| Give nitroglycerin | | | BP decreases to 95/60, chest pain improves slightly | | If give 2^nd^ nitro or nitro infusion, BP decreases again slightly to 92/55 and chest pain improves more | | | |
| After 4 minutes | | | Patient moans and goes into ventricular fibrillation | | Telemetry monitoring changes to ventricular fibrillation | | | |
| Shock at 200J | | | Telemetry monitoring changes to sinus with frequent PVCs (and short runs of NSVT), patient regains pulses | | HR 96, BP 61/49 | | | |
| Give antidysrhythmic medication (i.e. amiodarone, lidocaine) | | | NSVT stops (change on telemetry monitoring) | |  | | | |
| Start on pressors (can be norepinephrine, epinephrine, dopamine) | | | BP increases to 100/60 | |  | | | |
| Give IV fluids but not started on pressors | | | BP slightly increases to 70/53, SpO2 decreases to 89% on room air | |  | | | |

**Ideal Scenario Flow**

The learners enter the room to find a patient awake but in distress and complaining of chest pain. One learner designates himself or herself as the team leader and assigns roles to the other team members (one person for airway, one person to act as bedside nurse, one person to obtain history/exam). The bedside learner immediately places the patient on the monitor and obtains IV access. The team leader orders an EKG and labs while the history/exam learner obtains an appropriate history and performs a physical exam and relays pertinent information to the team leader. The airway learner places the patient on supplemental oxygen. The team recognizes that the EKG shows sinus rhythm with ST elevations in LAD distribution and immediately give aspirin and heparin, place pads on the patient, and call the cardiac catheterization lab. While waiting for the cath lab to be prepared, the patient becomes unresponsive and goes into cardiac arrest; the monitor changes to show ventricular fibrillation. CPR is started and the patient is given an antidysrhythmic medication and shocked at 200J with subsequent conversion and change in the telemetry monitor to sinus rhythm and return of pulses. The airway learner intubates the patient. The patient is hypotensive, so is started on pressors with improvement in blood pressure before transportation to the cath lab. All learners use closed-loop communication throughout the scenario. The team leader demonstrates situation awareness and clearly allocates resources and tasks throughout the scenario.

**Anticipated Management Mistakes**

1. Failure to place the pads on the patient before cardiac arrest: Some teams did not place pads on the patient immediately, leading to delay in defibrillation during arrest.
2. Uncertainty about which antidysrhythmic to use: We reviewed the different indications and dosages of antidysrhythmic medications that could be used in a case of ventricular fibrillation during the debrief.
3. Failure of the team leader to identify roles for the team members at the beginning of the case, used closed-loop communication, clearly allocate resources and tasks, and/or demonstrate situational awareness during the case: We reviewed the performance of the team leader and the team dynamics during the debrief after each case, including faculty observations regarding the application of CRM and TeamSTEPPS principles.
