# Supplementary material for: Resuscitation Leadership Training: A Simulation Curriculum for Emergency Medicine Residents
Source: MedEdPORTAL. 2022 Oct 11;18:11278. doi: 10.15766/mep_2374-8265.11278 (PMC9550795; doi:10.15766/mep_2374-8265.11278)
Supplement: Supplementary file 1 — Sim Case - STEMI and VFib Arrest.docxCase Media and Labs - STEMI and VFib Arrest.pptxSim Case - Massive Pulmonary Embolism.docxCase Media and Labs - Massive PE.pptxSim Case - Wide Complex Tachycardia.docxCase Media and Labs - WCT.pptxSim Case - Missed Dialysis.docxCase Media and Labs - Missed Dialysis.pptxCAC - STEMI and VFib Arrest.docxCAC - Massive Pulmonary Embolism.docxCAC - Wide Complex Tachycardia.docxCAC - Missed Dialysis.docxCRM Presentation.pptxDebrief Handout.pdfSelect ACGME EM Milestones List.pptxOttawa GRS.docxResident Survey.docx [file mep_2374-8265.11278-s001.zip › K. CAC - Wide Complex Tachycardia.docx]

**Critical Actions Checklist: Wide Complex Tachycardia**

Leader __________________

1. Y/N Recognize wide complex tachycardia on EKG
2. Y/N Place defibrillation pads on patient immediately
3. Y/N Give an appropriate antidysrhythmic medication (amiodarone, procainamide, lidocaine, or beta-blocker)
4. Y/N Give a second appropriate antidysrhythmic medication (amiodarone, procainamide, lidocaine, or beta-blocker)
5. Y/N Administer synchronized cardioversion when patient becomes unstable
6. Y/N Explain cardioversion to patient prior to shocking
7. Y/N Order labs including troponin and electrolytes
8. Y/N Monitor oxygen saturation and telemetry during cardioversion
9. Y/N Obtain a repeat EKG after cardioversion
10. Y/N Consult and admit to cardiology
