# Supplementary material for: Resuscitation Leadership Training: A Simulation Curriculum for Emergency Medicine Residents
Source: MedEdPORTAL. 2022 Oct 11;18:11278. doi: 10.15766/mep_2374-8265.11278 (PMC9550795; doi:10.15766/mep_2374-8265.11278)
Supplement: Supplementary file 1 — Sim Case - STEMI and VFib Arrest.docxCase Media and Labs - STEMI and VFib Arrest.pptxSim Case - Massive Pulmonary Embolism.docxCase Media and Labs - Massive PE.pptxSim Case - Wide Complex Tachycardia.docxCase Media and Labs - WCT.pptxSim Case - Missed Dialysis.docxCase Media and Labs - Missed Dialysis.pptxCAC - STEMI and VFib Arrest.docxCAC - Massive Pulmonary Embolism.docxCAC - Wide Complex Tachycardia.docxCAC - Missed Dialysis.docxCRM Presentation.pptxDebrief Handout.pdfSelect ACGME EM Milestones List.pptxOttawa GRS.docxResident Survey.docx [file mep_2374-8265.11278-s001.zip › L. CAC - Missed Dialysis.docx]

**Critical Actions Checklist: Missed Dialysis**

Leader __________________

1. Y/N Obtain an EKG
2. Y/N Place patient on positive pressure ventilation (BiPAP, CPAP, or intubation)
3. Y/N Give nitroglycerin (sublingual or infusion)
4. Y/N Obtain labs including VBG, K, troponin
5. Y/N Obtain pulmonary imaging (POCUS and/or CXR)
6. Y/N Give calcium gluconate IV
7. Y/N Give medications for temporizing hyperkalemia (insulin, albuterol, and/or bicarb)
8. Y/N Give medication to eliminate potassium (furosemide or kayexalate)
9. Y/N Call nephrology for emergent hemodialysis
10. Y/N Admit to ICU
