# Supplementary material for: Resuscitation Leadership Training: A Simulation Curriculum for Emergency Medicine Residents
Source: MedEdPORTAL. 2022 Oct 11;18:11278. doi: 10.15766/mep_2374-8265.11278 (PMC9550795; doi:10.15766/mep_2374-8265.11278)
Supplement: Supplementary file 1 — Sim Case - STEMI and VFib Arrest.docxCase Media and Labs - STEMI and VFib Arrest.pptxSim Case - Massive Pulmonary Embolism.docxCase Media and Labs - Massive PE.pptxSim Case - Wide Complex Tachycardia.docxCase Media and Labs - WCT.pptxSim Case - Missed Dialysis.docxCase Media and Labs - Missed Dialysis.pptxCAC - STEMI and VFib Arrest.docxCAC - Massive Pulmonary Embolism.docxCAC - Wide Complex Tachycardia.docxCAC - Missed Dialysis.docxCRM Presentation.pptxDebrief Handout.pdfSelect ACGME EM Milestones List.pptxOttawa GRS.docxResident Survey.docx [file mep_2374-8265.11278-s001.zip › M. CRM Presentation.pptx]

## Slide 1
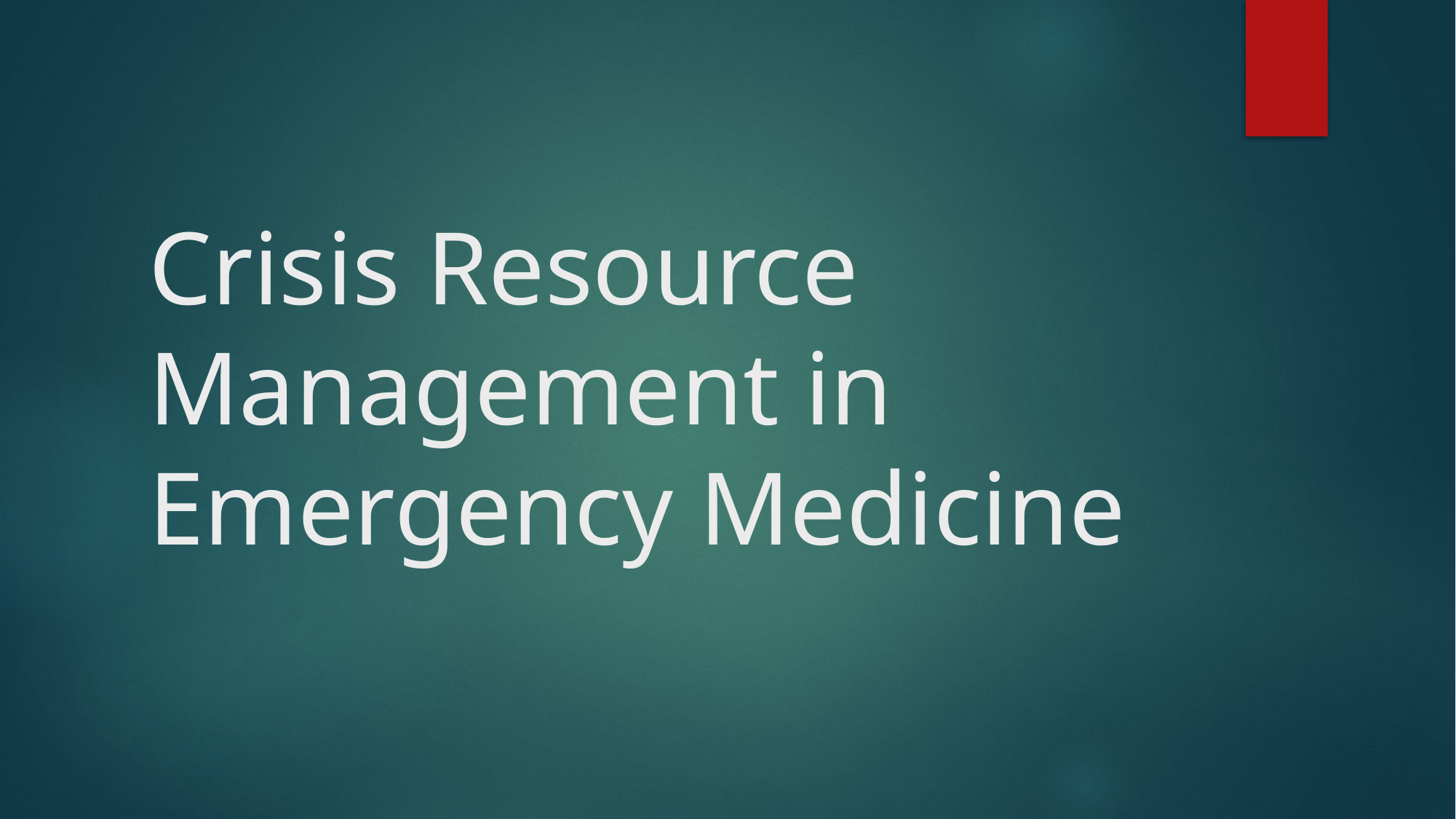

# Crisis Resource Management in Emergency Medicine

## Slide 2
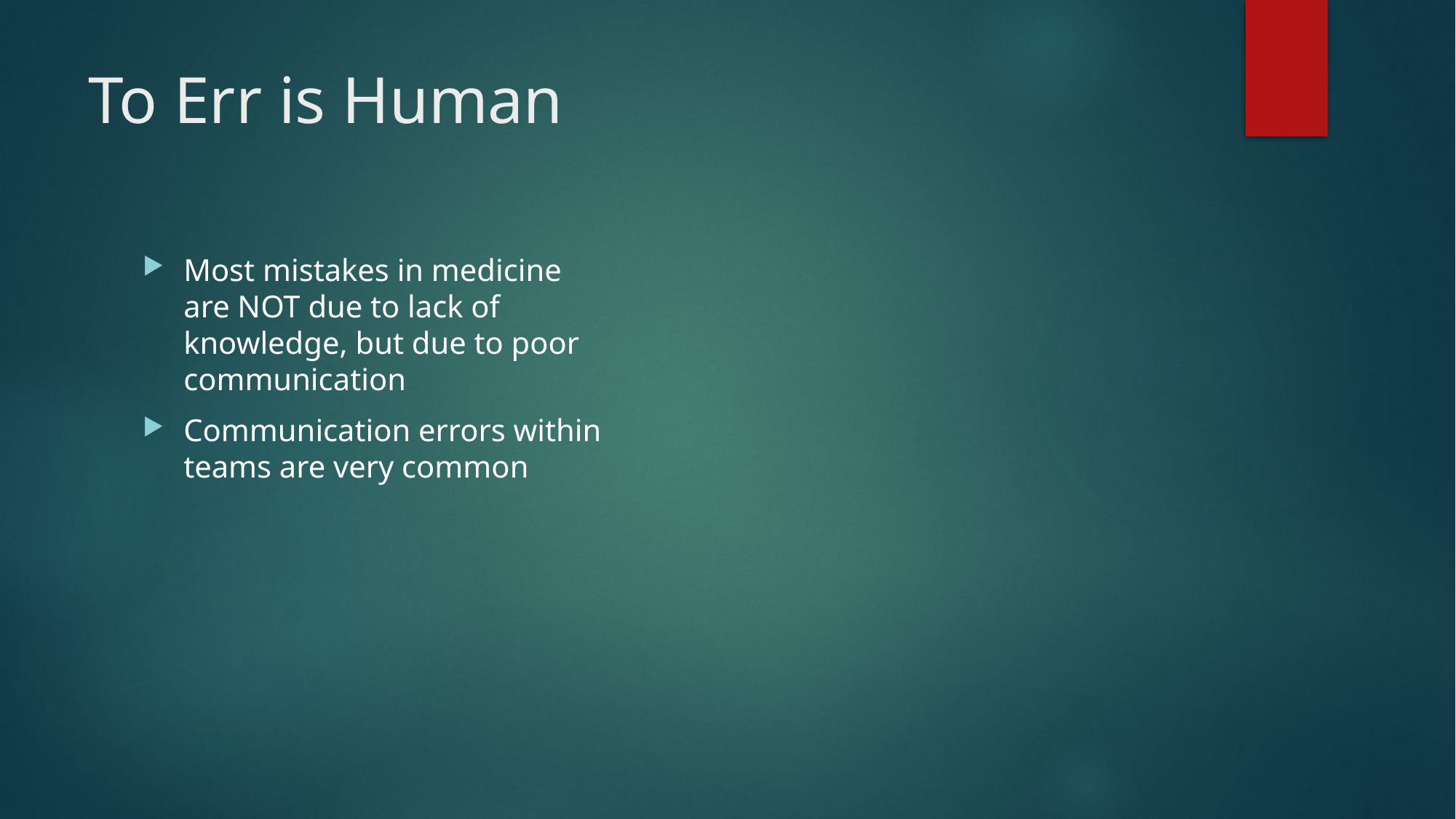

# To Err is Human
Most mistakes in medicine are NOT due to lack of knowledge, but due to poor communication
Communication errors within teams are very common

## Slide 3
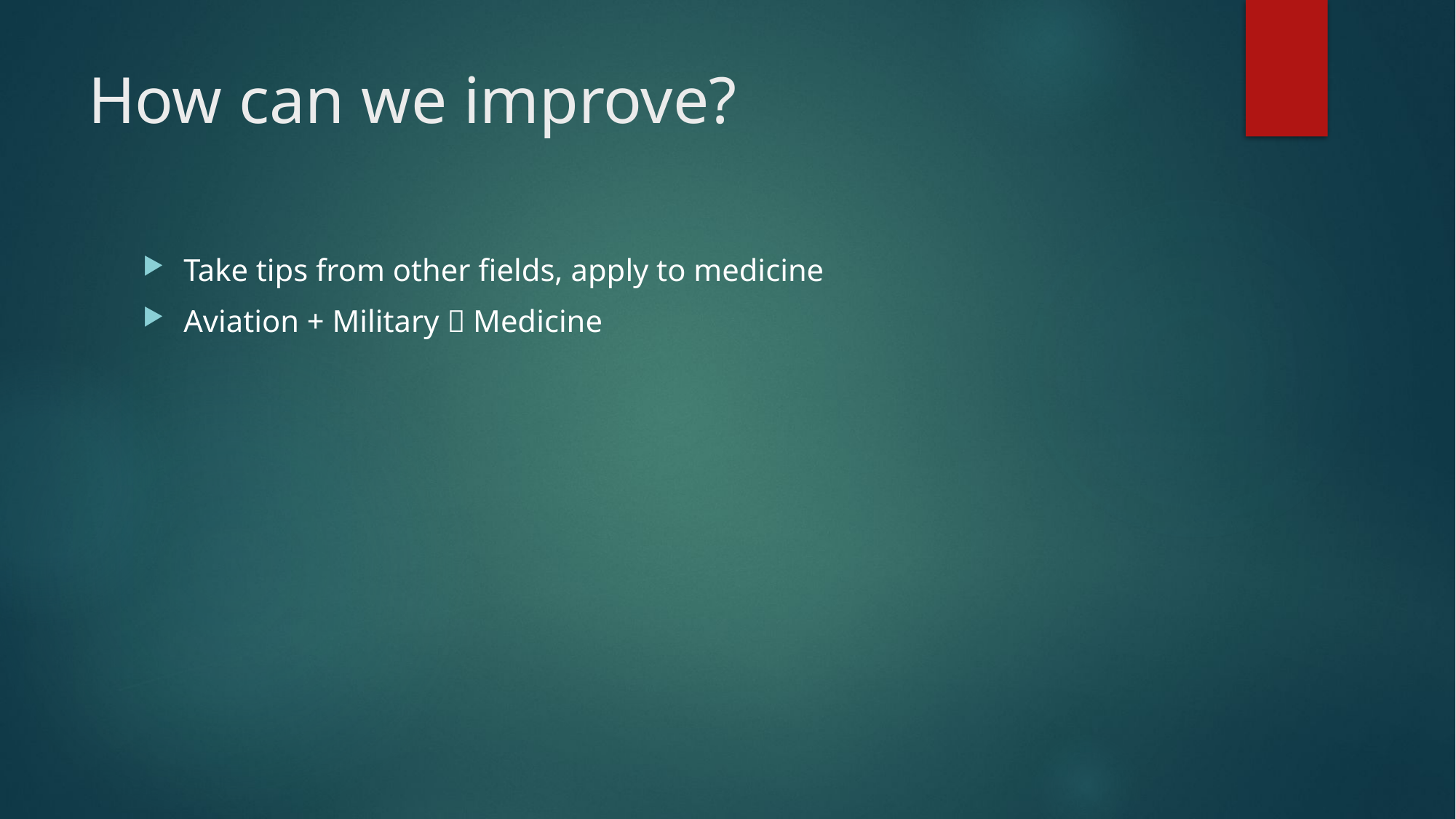

# How can we improve?
Take tips from other fields, apply to medicine
Aviation + Military  Medicine

## Slide 4
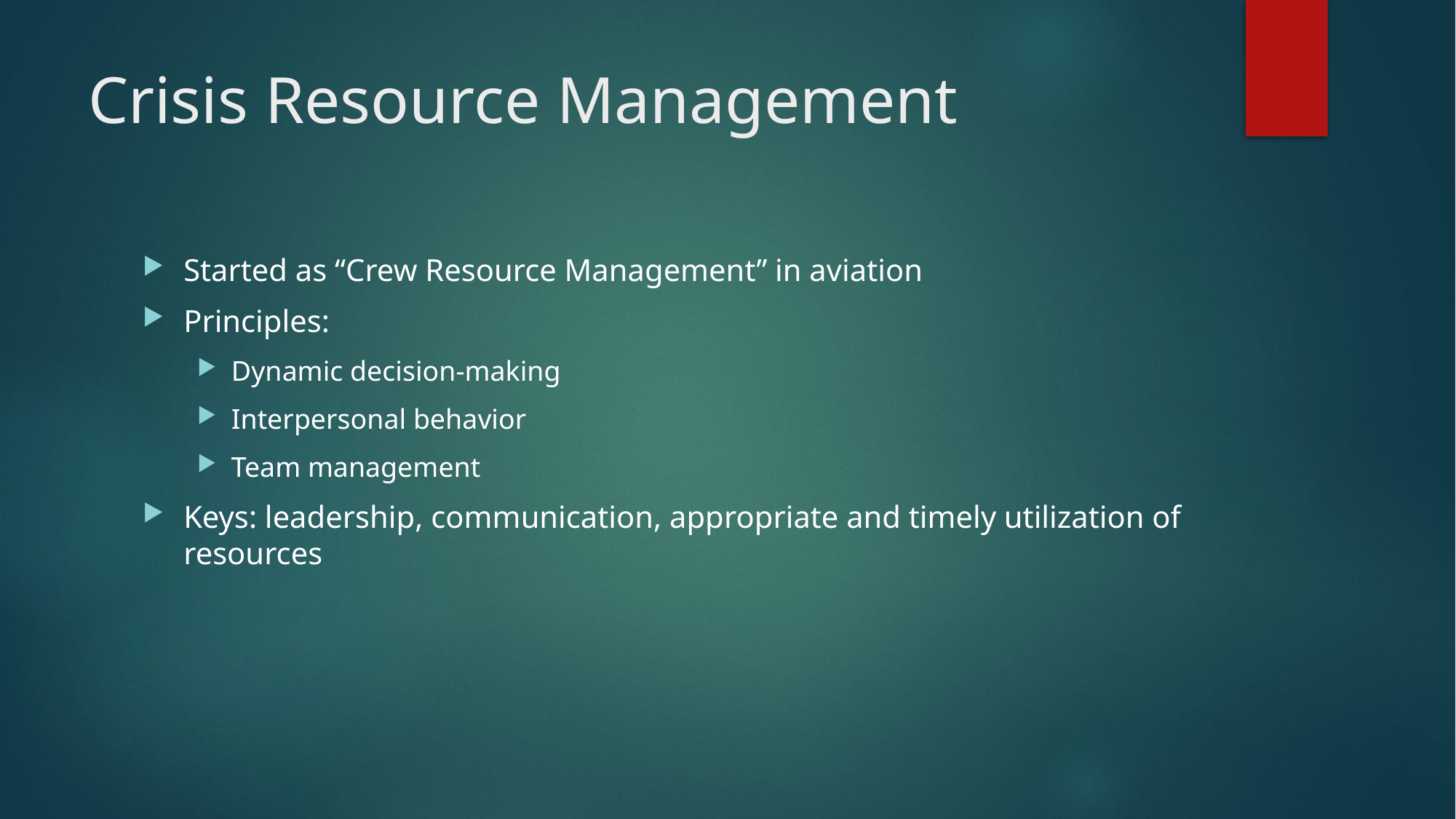

# Crisis Resource Management
Started as “Crew Resource Management” in aviation
Principles:
Dynamic decision-making
Interpersonal behavior
Team management
Keys: leadership, communication, appropriate and timely utilization of resources

## Slide 5
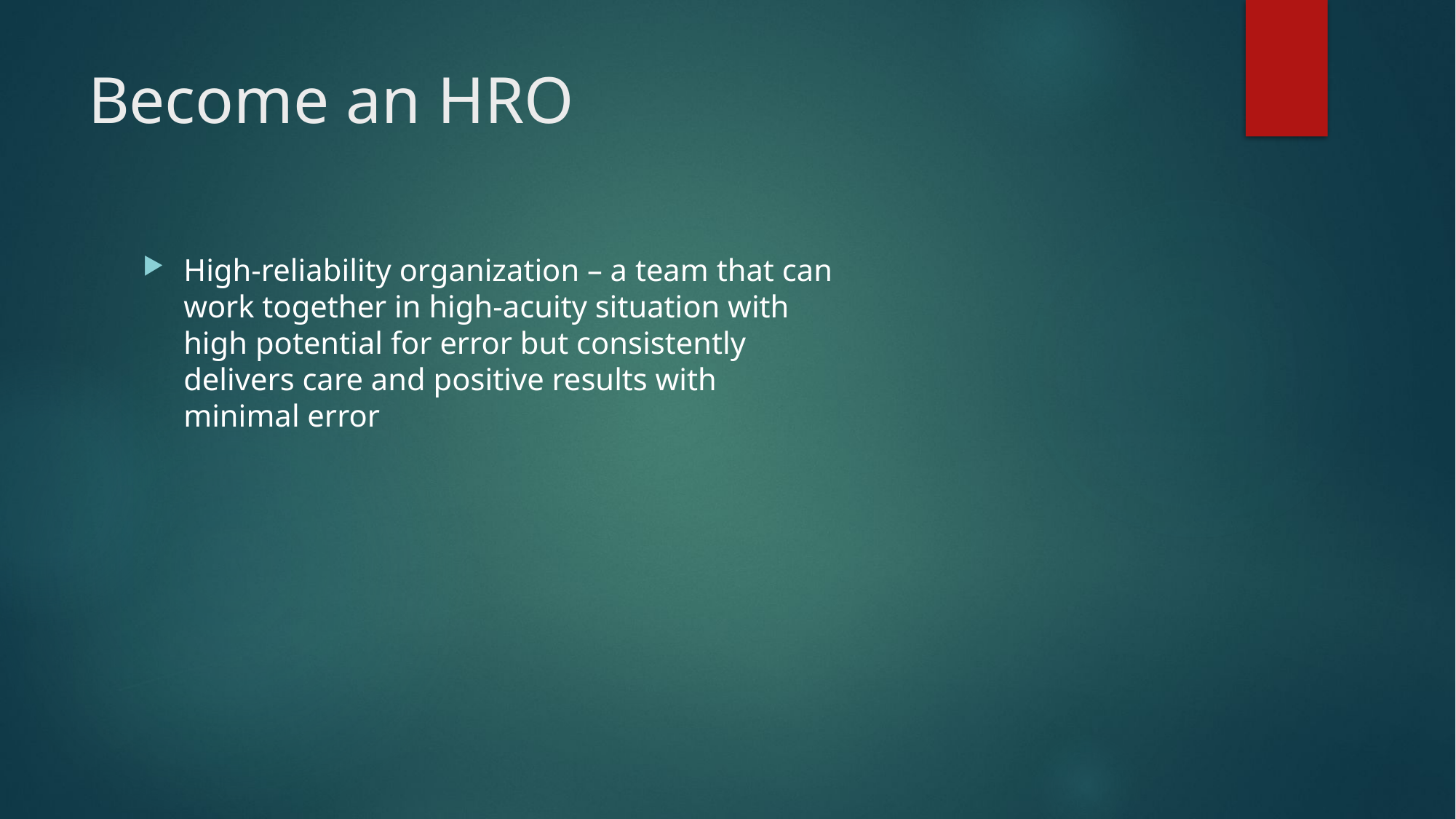

# Become an HRO
High-reliability organization – a team that can work together in high-acuity situation with high potential for error but consistently delivers care and positive results with minimal error

## Slide 6
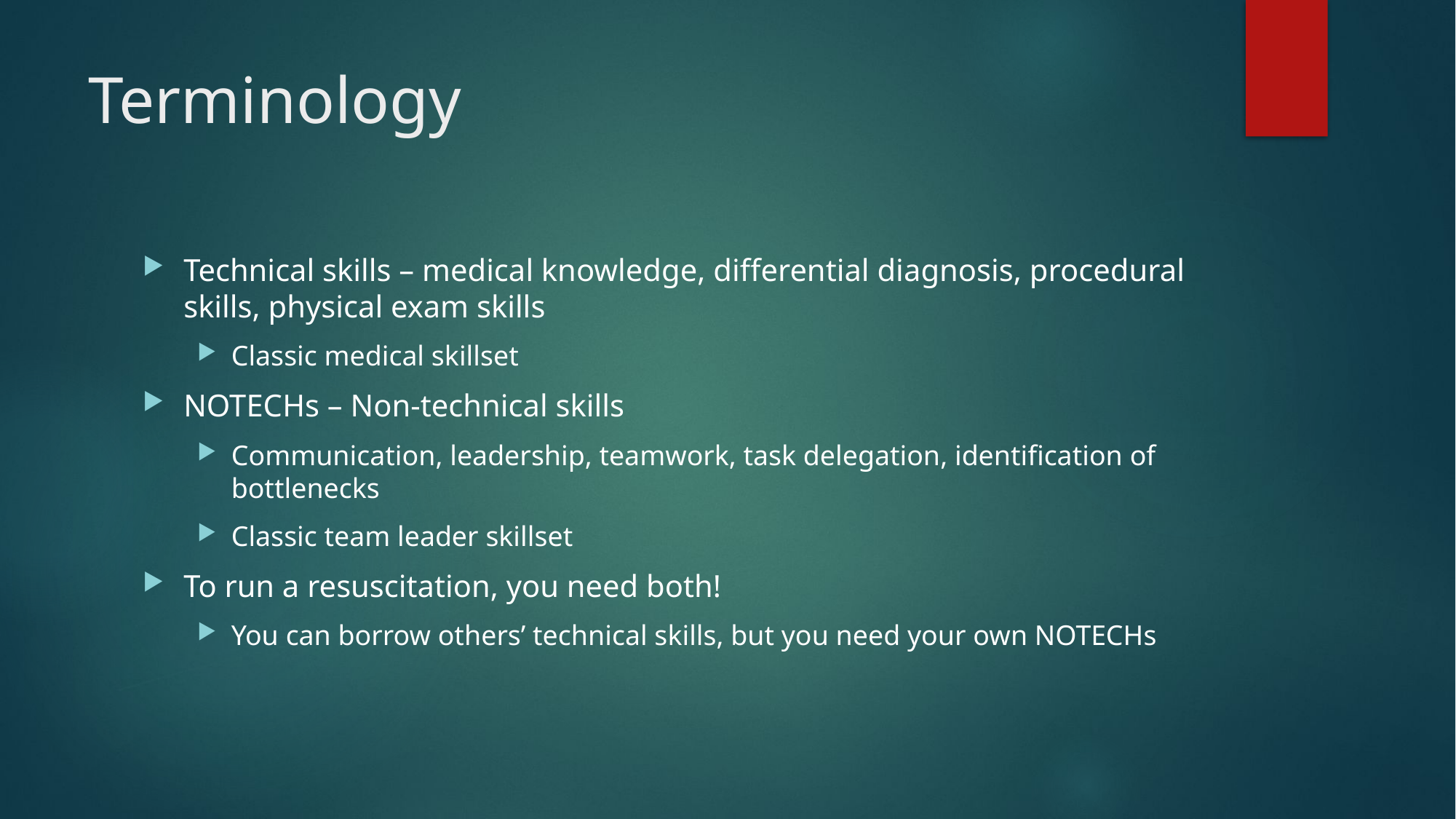

# Terminology
Technical skills – medical knowledge, differential diagnosis, procedural skills, physical exam skills
Classic medical skillset
NOTECHs – Non-technical skills
Communication, leadership, teamwork, task delegation, identification of bottlenecks
Classic team leader skillset
To run a resuscitation, you need both!
You can borrow others’ technical skills, but you need your own NOTECHs

## Slide 7
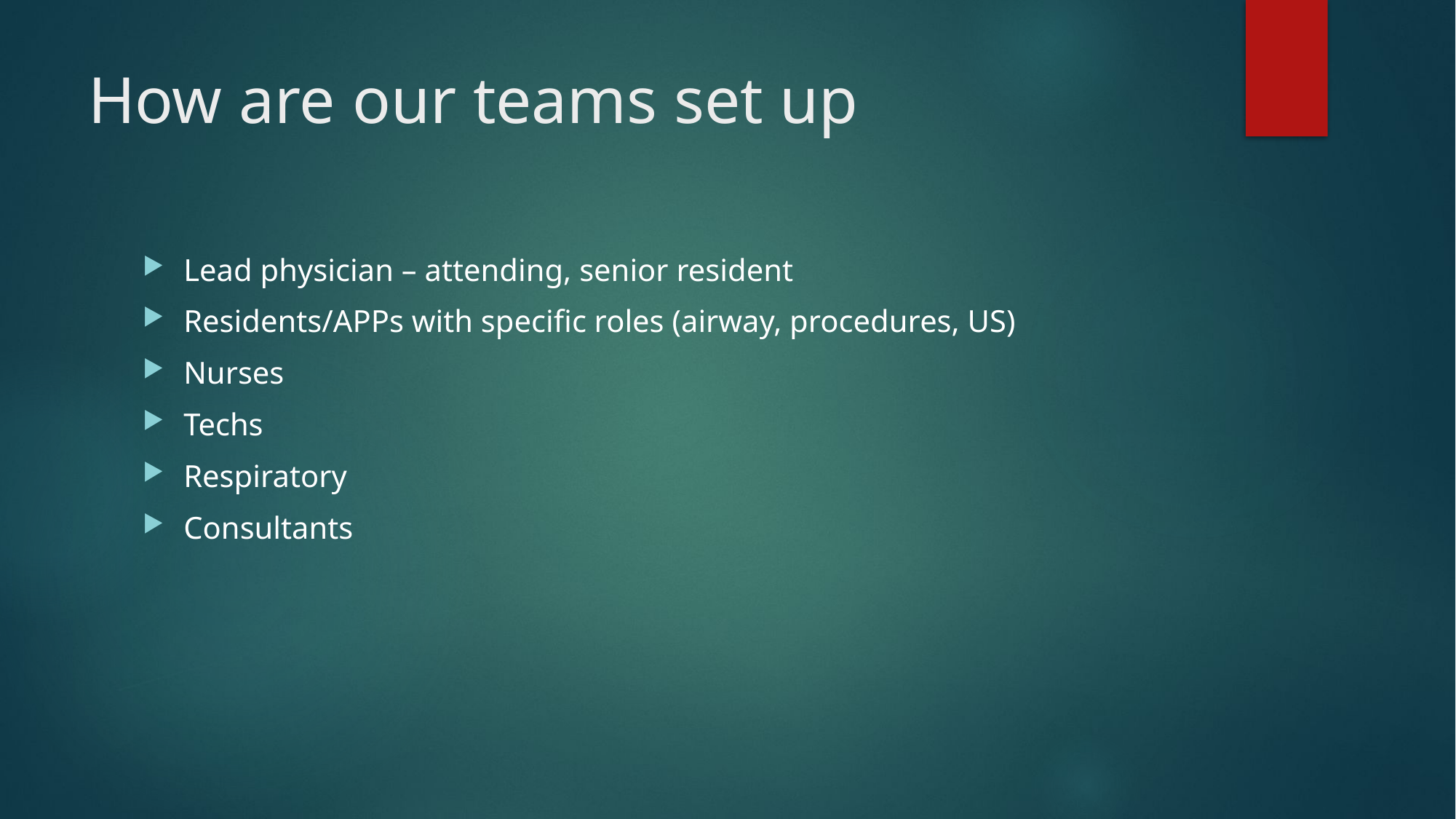

# How are our teams set up
Lead physician – attending, senior resident
Residents/APPs with specific roles (airway, procedures, US)
Nurses
Techs
Respiratory
Consultants

## Slide 8
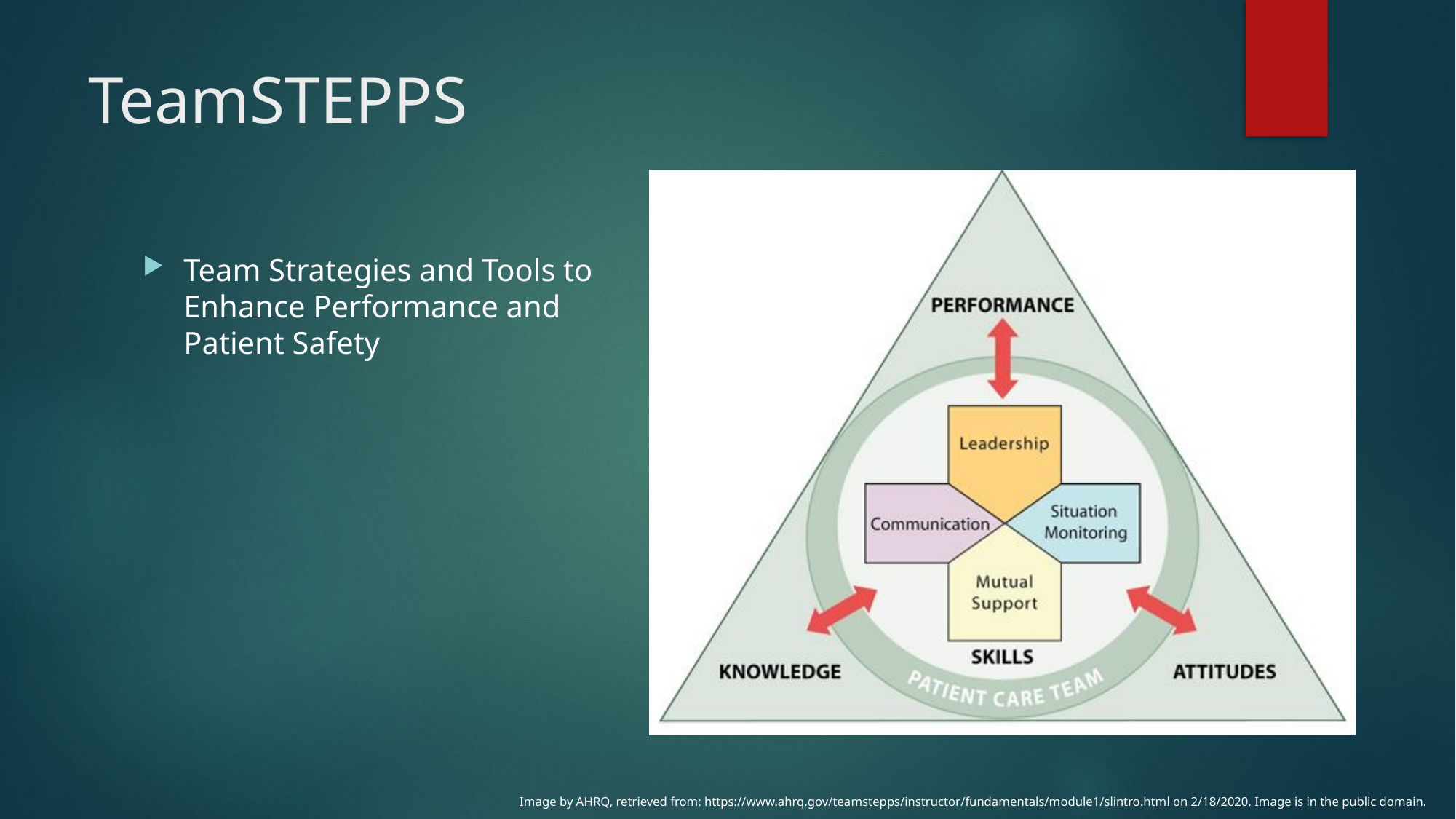

# TeamSTEPPS
Team Strategies and Tools to Enhance Performance and Patient Safety
Image by AHRQ, retrieved from: https://www.ahrq.gov/teamstepps/instructor/fundamentals/module1/slintro.html on 2/18/2020. Image is in the public domain.

## Slide 9
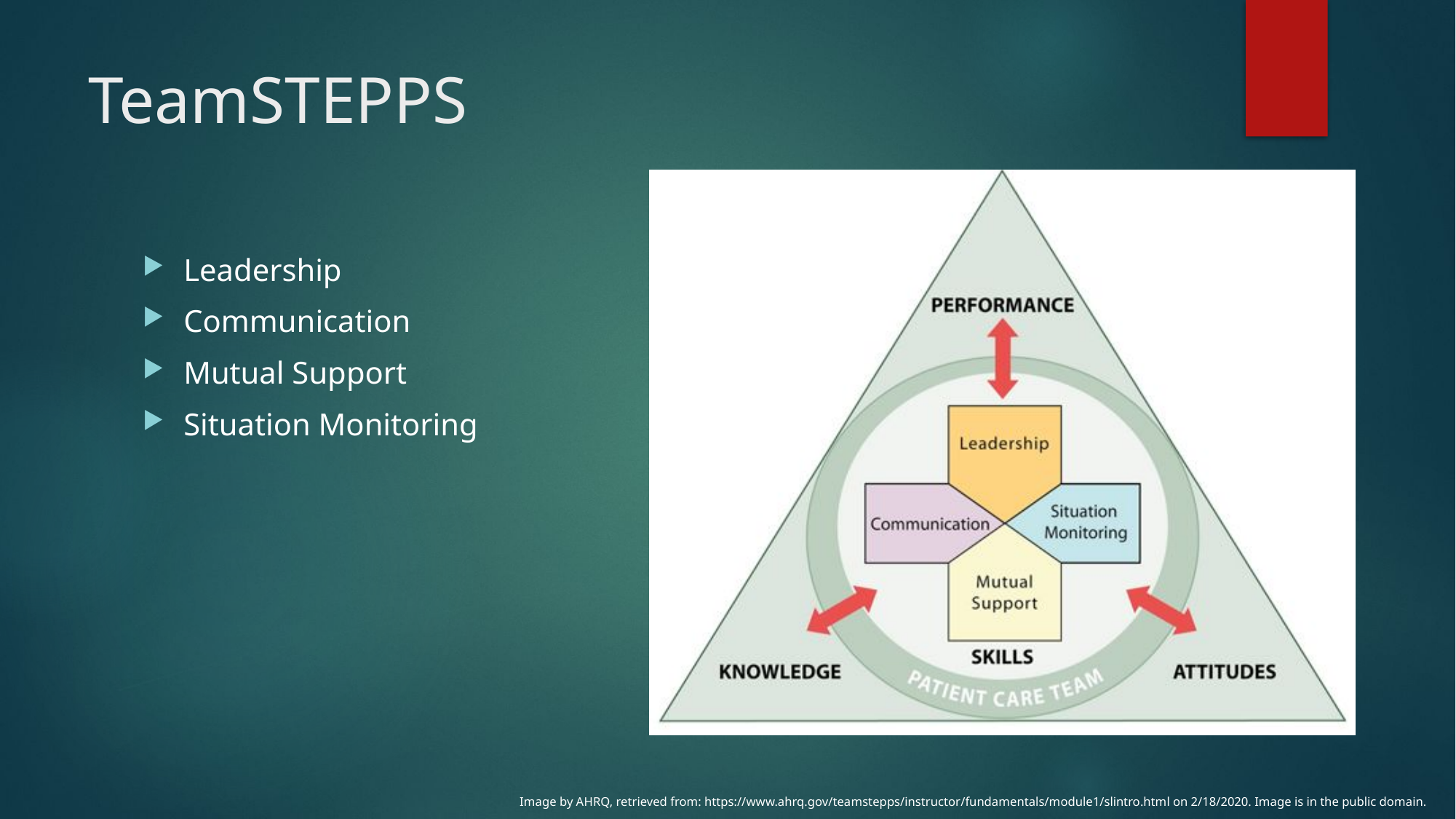

# TeamSTEPPS
Leadership
Communication
Mutual Support
Situation Monitoring
Image by AHRQ, retrieved from: https://www.ahrq.gov/teamstepps/instructor/fundamentals/module1/slintro.html on 2/18/2020. Image is in the public domain.

## Slide 10
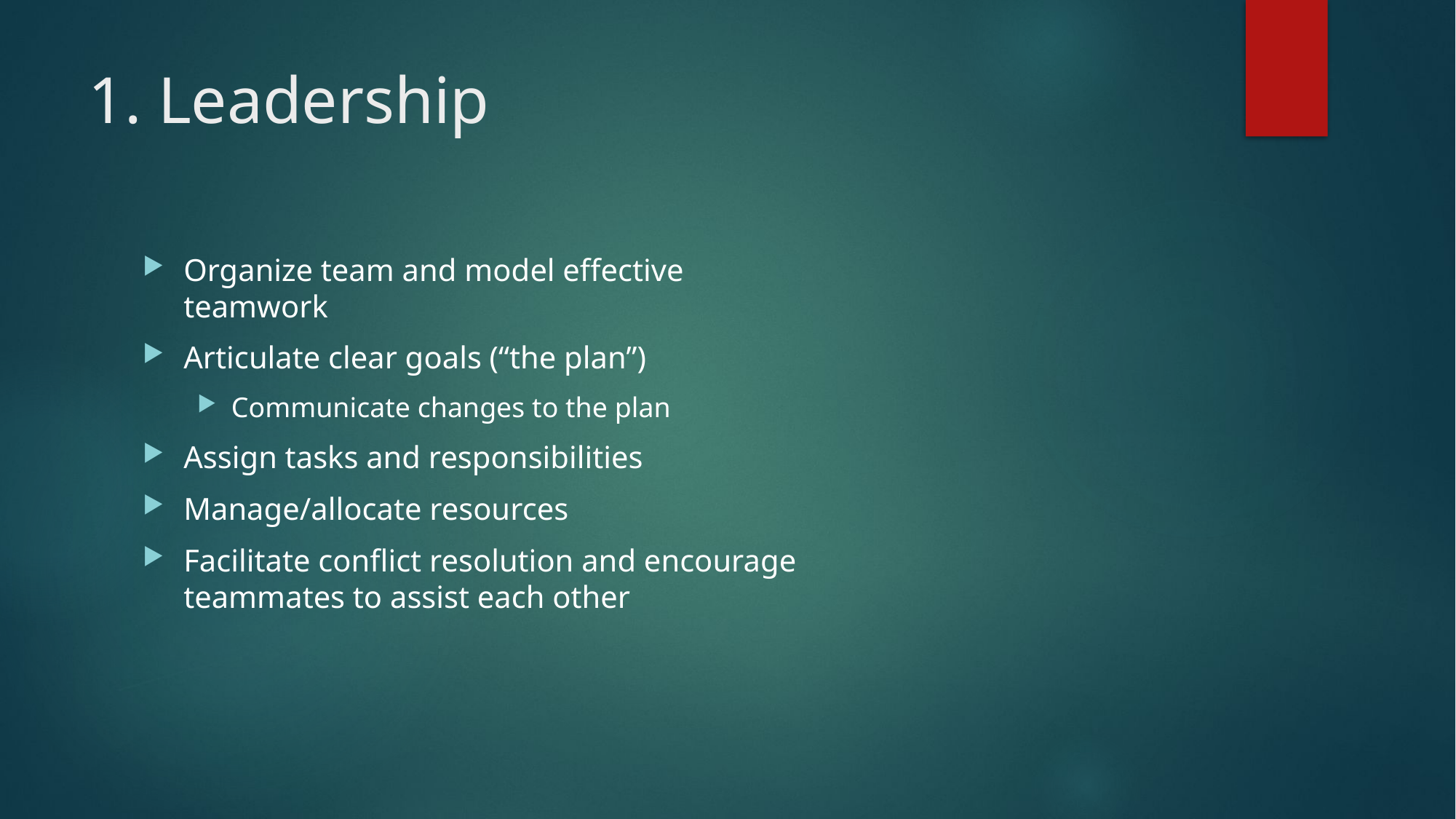

# 1. Leadership
Organize team and model effective teamwork
Articulate clear goals (“the plan”)
Communicate changes to the plan
Assign tasks and responsibilities
Manage/allocate resources
Facilitate conflict resolution and encourage teammates to assist each other

## Slide 11
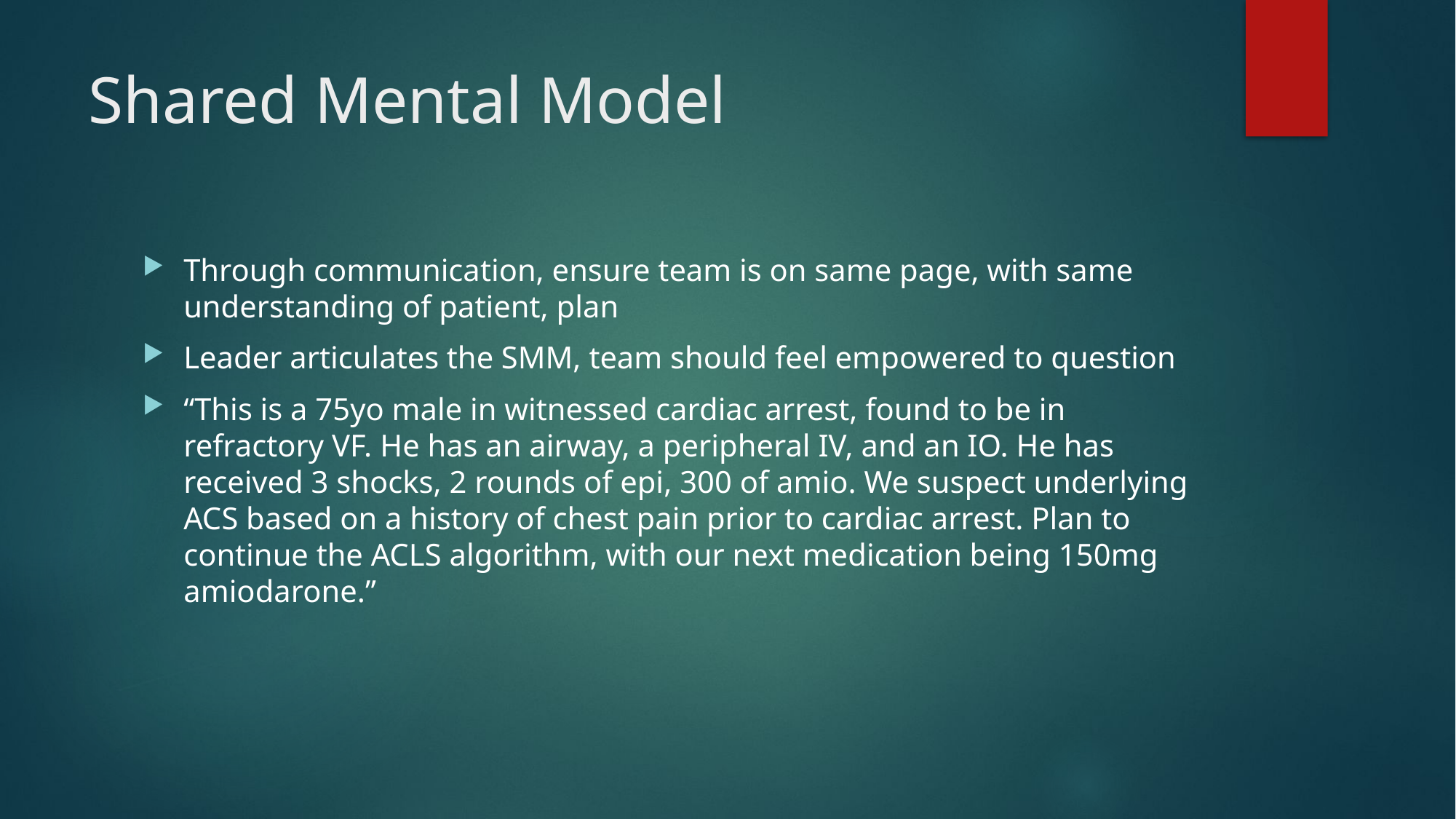

# Shared Mental Model
Through communication, ensure team is on same page, with same understanding of patient, plan
Leader articulates the SMM, team should feel empowered to question
“This is a 75yo male in witnessed cardiac arrest, found to be in refractory VF. He has an airway, a peripheral IV, and an IO. He has received 3 shocks, 2 rounds of epi, 300 of amio. We suspect underlying ACS based on a history of chest pain prior to cardiac arrest. Plan to continue the ACLS algorithm, with our next medication being 150mg amiodarone.”

## Slide 12
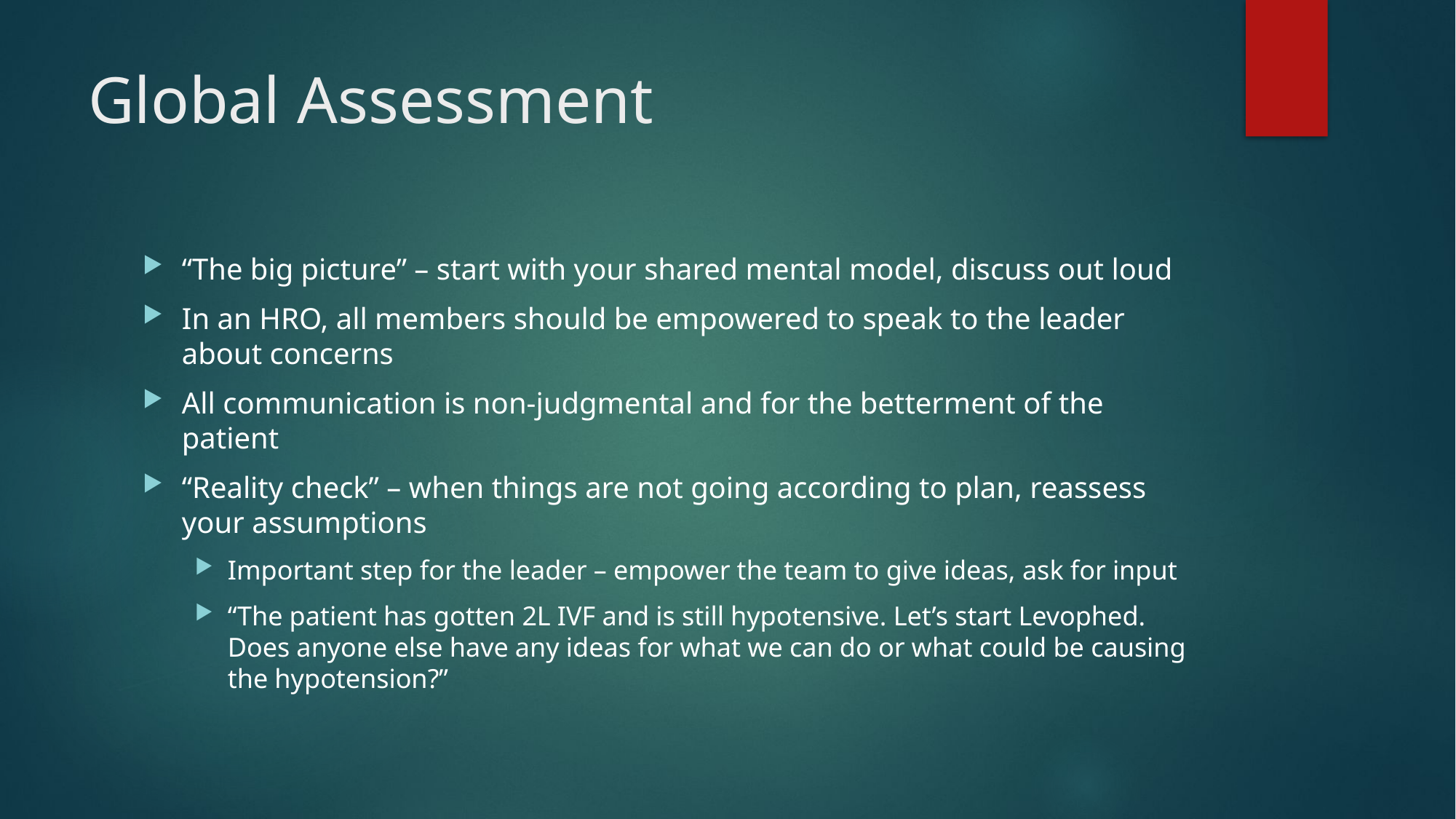

# Global Assessment
“The big picture” – start with your shared mental model, discuss out loud
In an HRO, all members should be empowered to speak to the leader about concerns
All communication is non-judgmental and for the betterment of the patient
“Reality check” – when things are not going according to plan, reassess your assumptions
Important step for the leader – empower the team to give ideas, ask for input
“The patient has gotten 2L IVF and is still hypotensive. Let’s start Levophed. Does anyone else have any ideas for what we can do or what could be causing the hypotension?”

## Slide 13
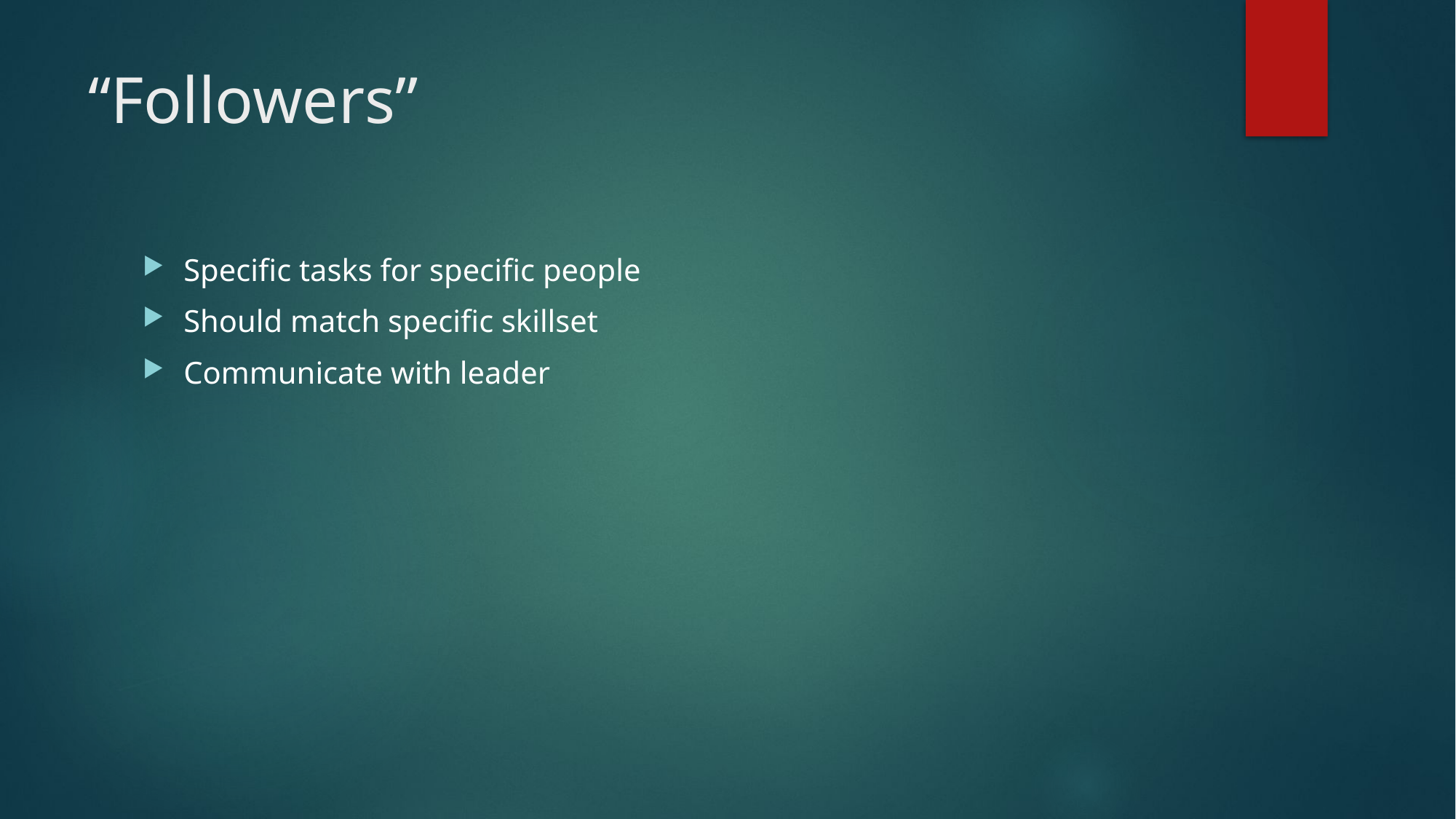

# “Followers”
Specific tasks for specific people
Should match specific skillset
Communicate with leader

## Slide 14
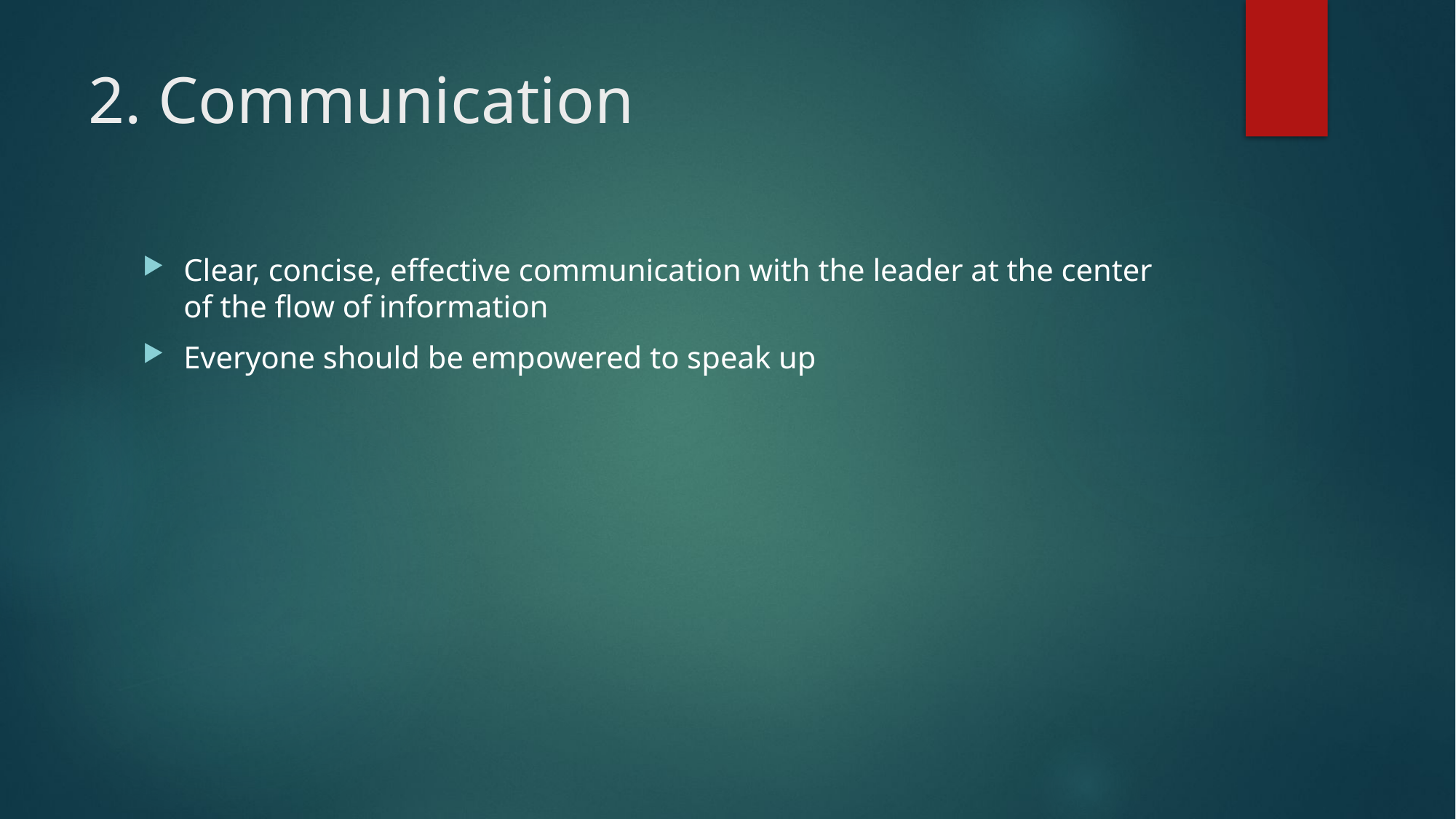

# 2. Communication
Clear, concise, effective communication with the leader at the center of the flow of information
Everyone should be empowered to speak up

## Slide 15
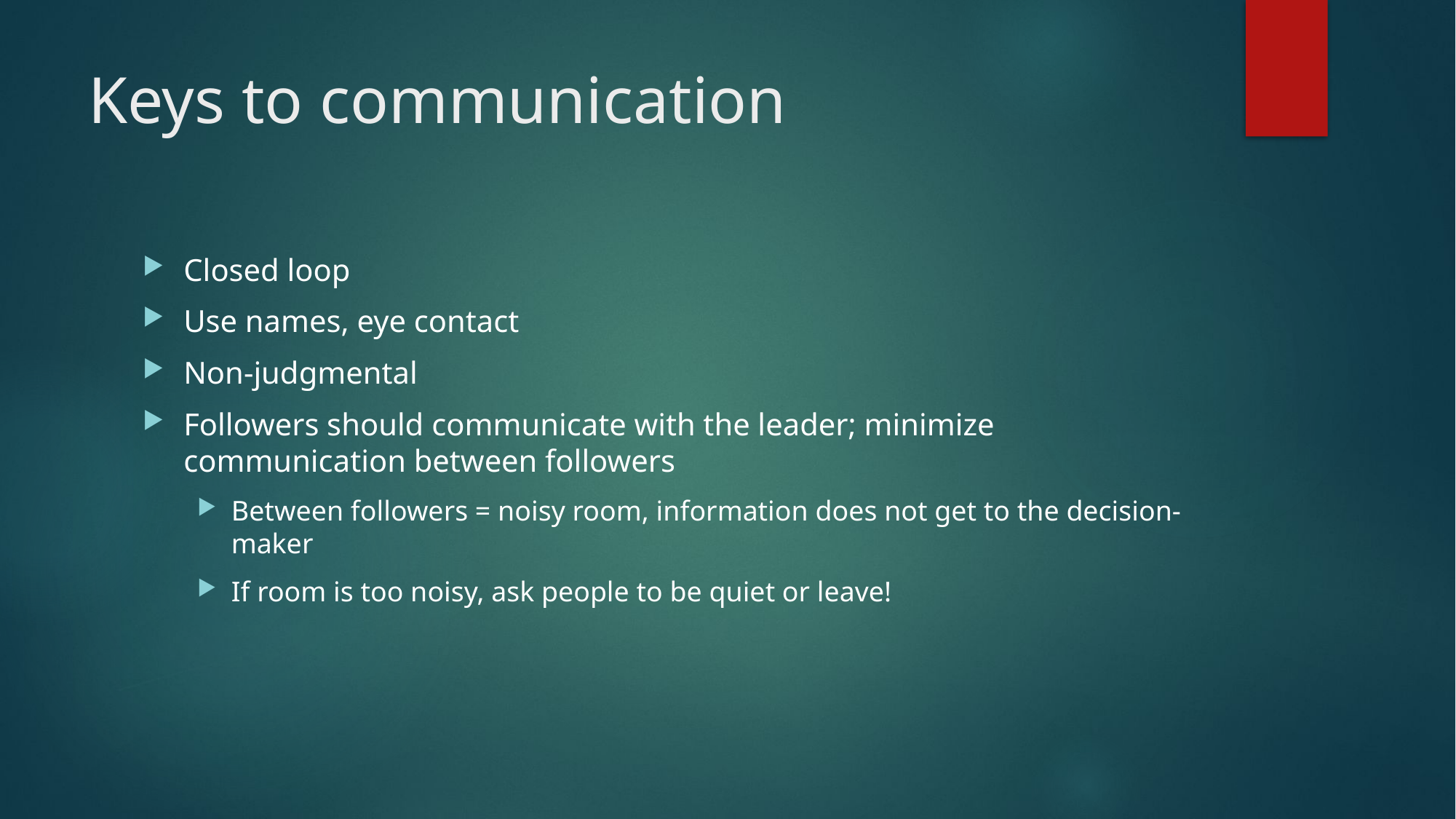

# Keys to communication
Closed loop
Use names, eye contact
Non-judgmental
Followers should communicate with the leader; minimize communication between followers
Between followers = noisy room, information does not get to the decision-maker
If room is too noisy, ask people to be quiet or leave!

## Slide 16
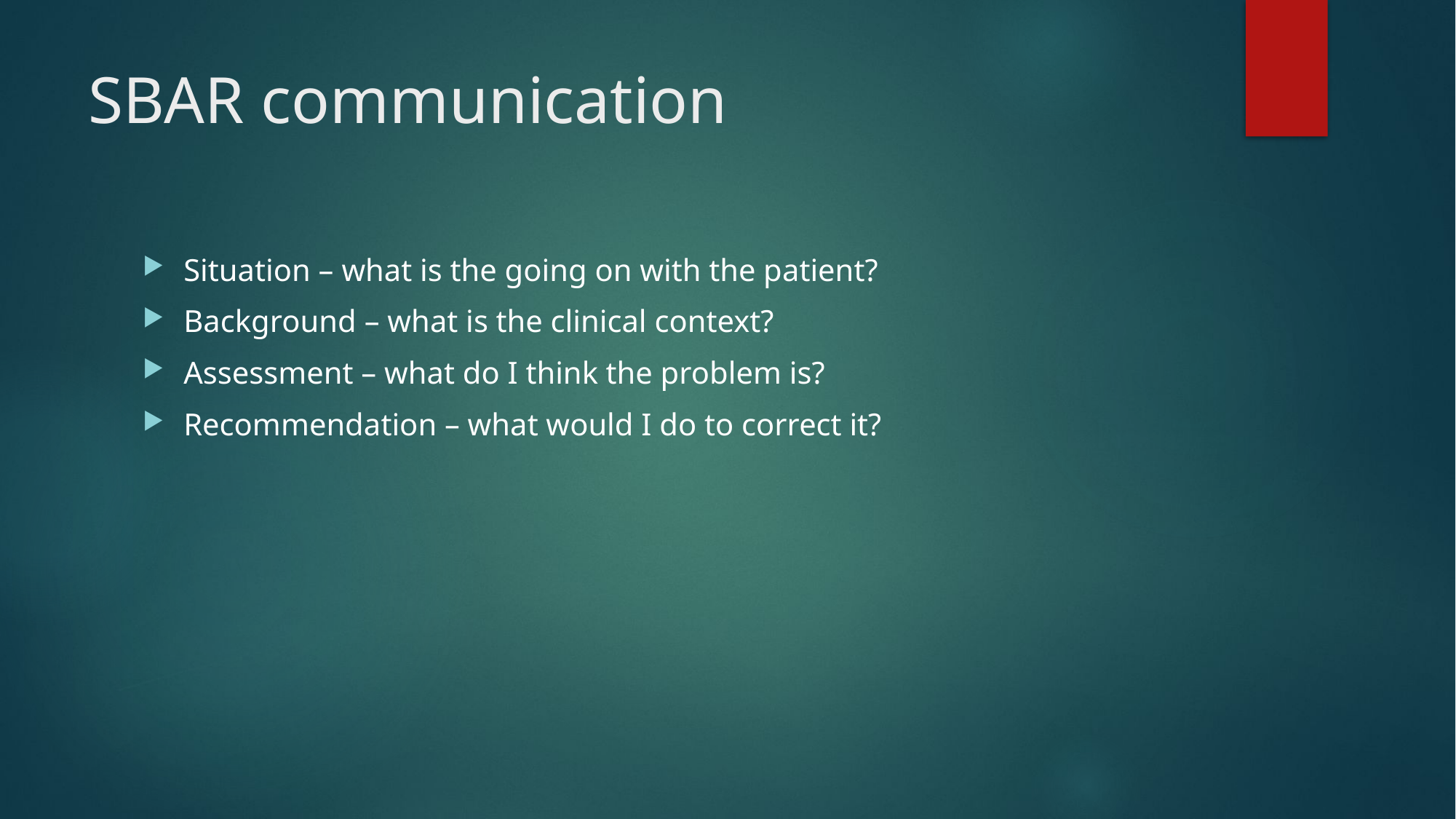

# SBAR communication
Situation – what is the going on with the patient?
Background – what is the clinical context?
Assessment – what do I think the problem is?
Recommendation – what would I do to correct it?

## Slide 17
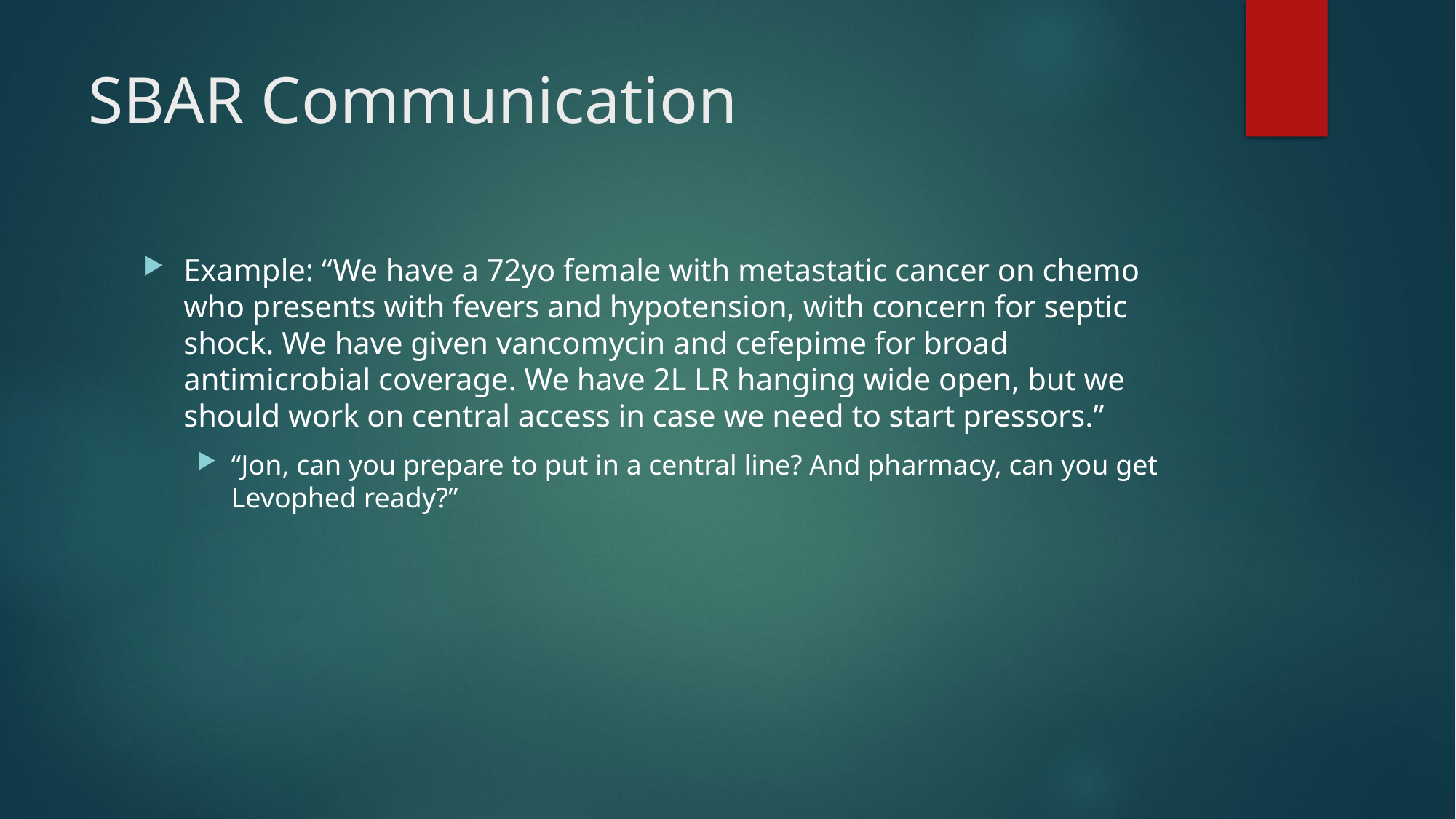

# SBAR Communication
Example: “We have a 72yo female with metastatic cancer on chemo who presents with fevers and hypotension, with concern for septic shock. We have given vancomycin and cefepime for broad antimicrobial coverage. We have 2L LR hanging wide open, but we should work on central access in case we need to start pressors.”
“Jon, can you prepare to put in a central line? And pharmacy, can you get Levophed ready?”

## Slide 18
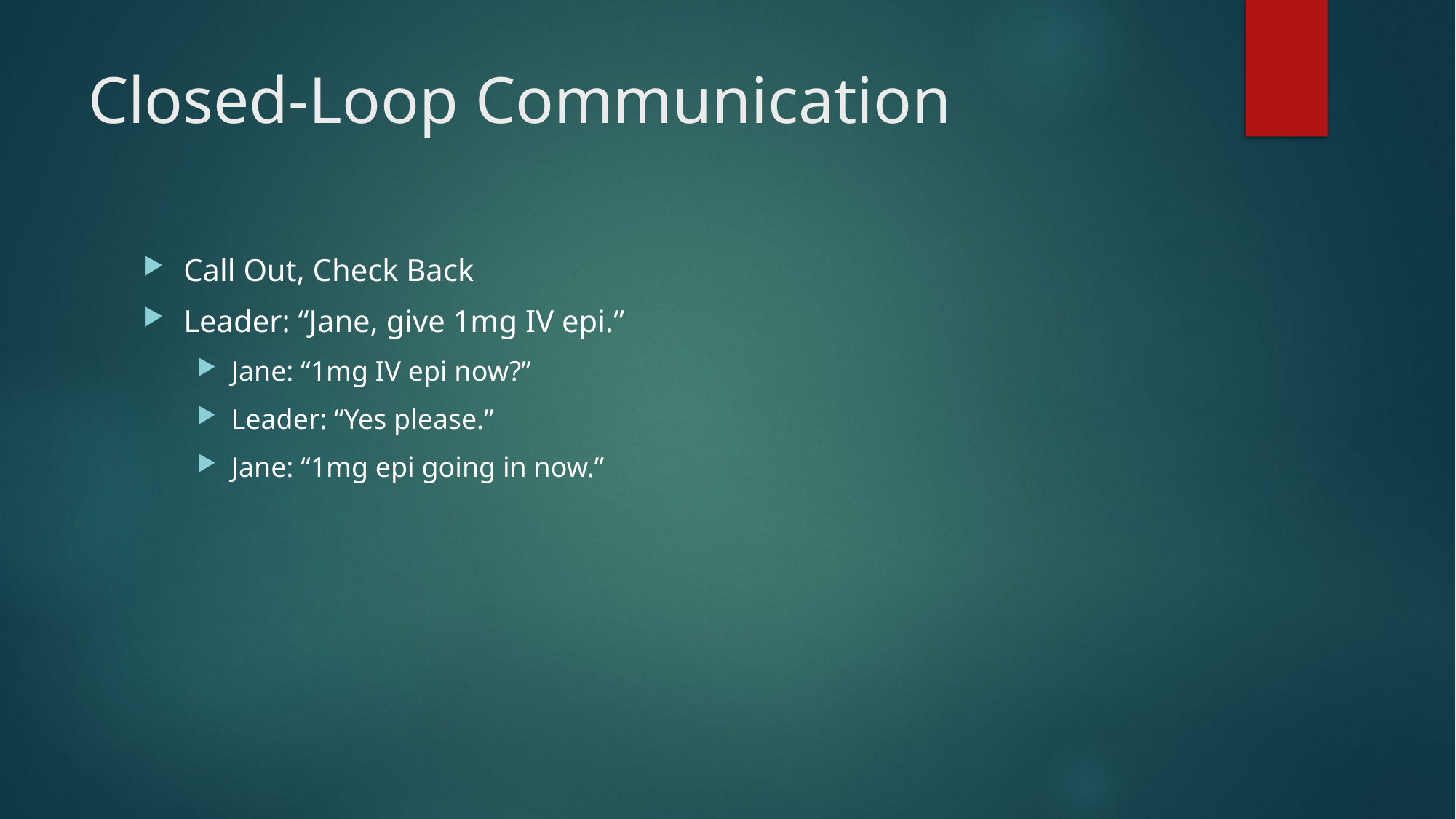

# Closed-Loop Communication
Call Out, Check Back
Leader: “Jane, give 1mg IV epi.”
Jane: “1mg IV epi now?”
Leader: “Yes please.”
Jane: “1mg epi going in now.”

## Slide 19
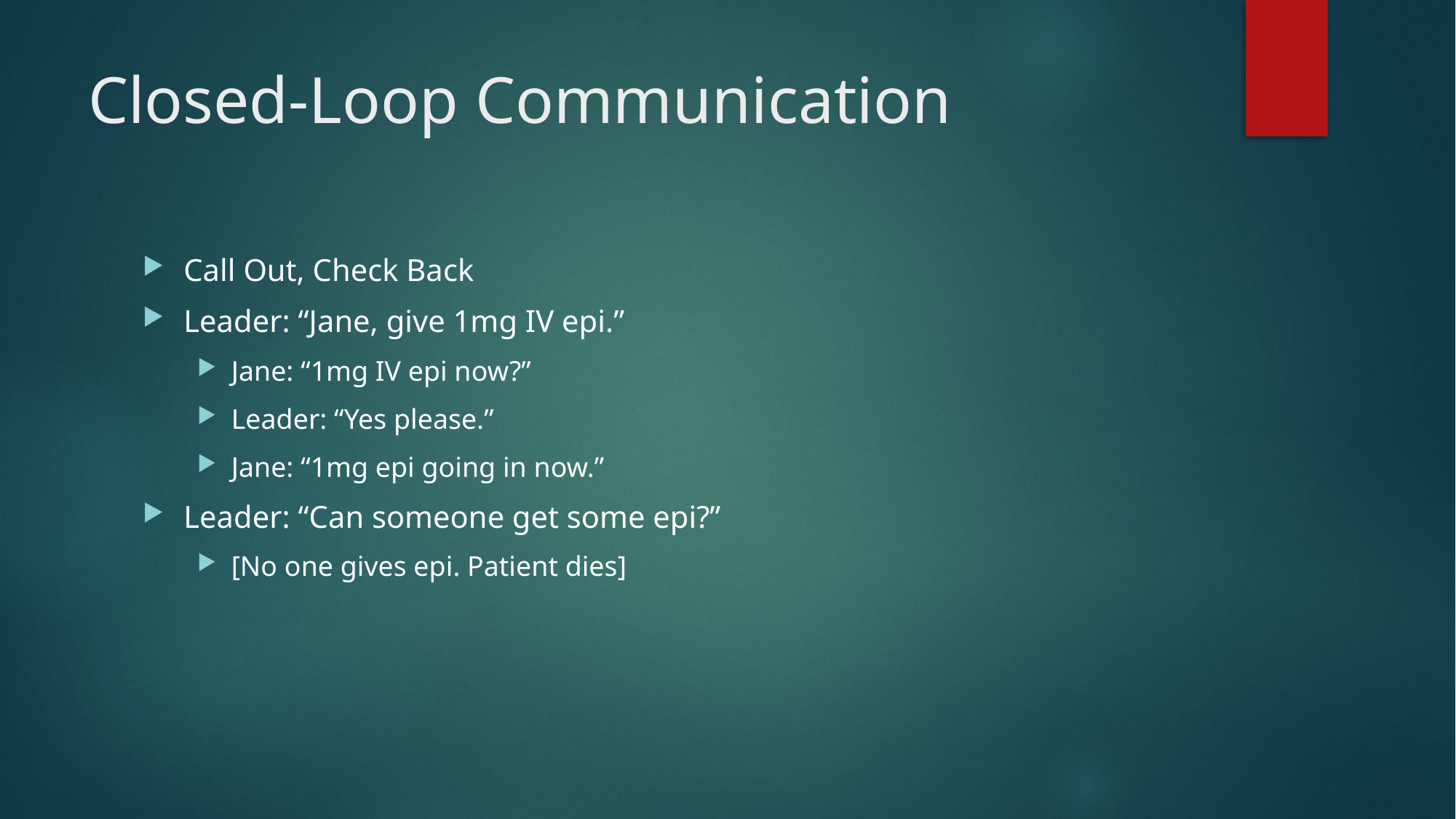

# Closed-Loop Communication
Call Out, Check Back
Leader: “Jane, give 1mg IV epi.”
Jane: “1mg IV epi now?”
Leader: “Yes please.”
Jane: “1mg epi going in now.”
Leader: “Can someone get some epi?”
[No one gives epi. Patient dies]

## Slide 20
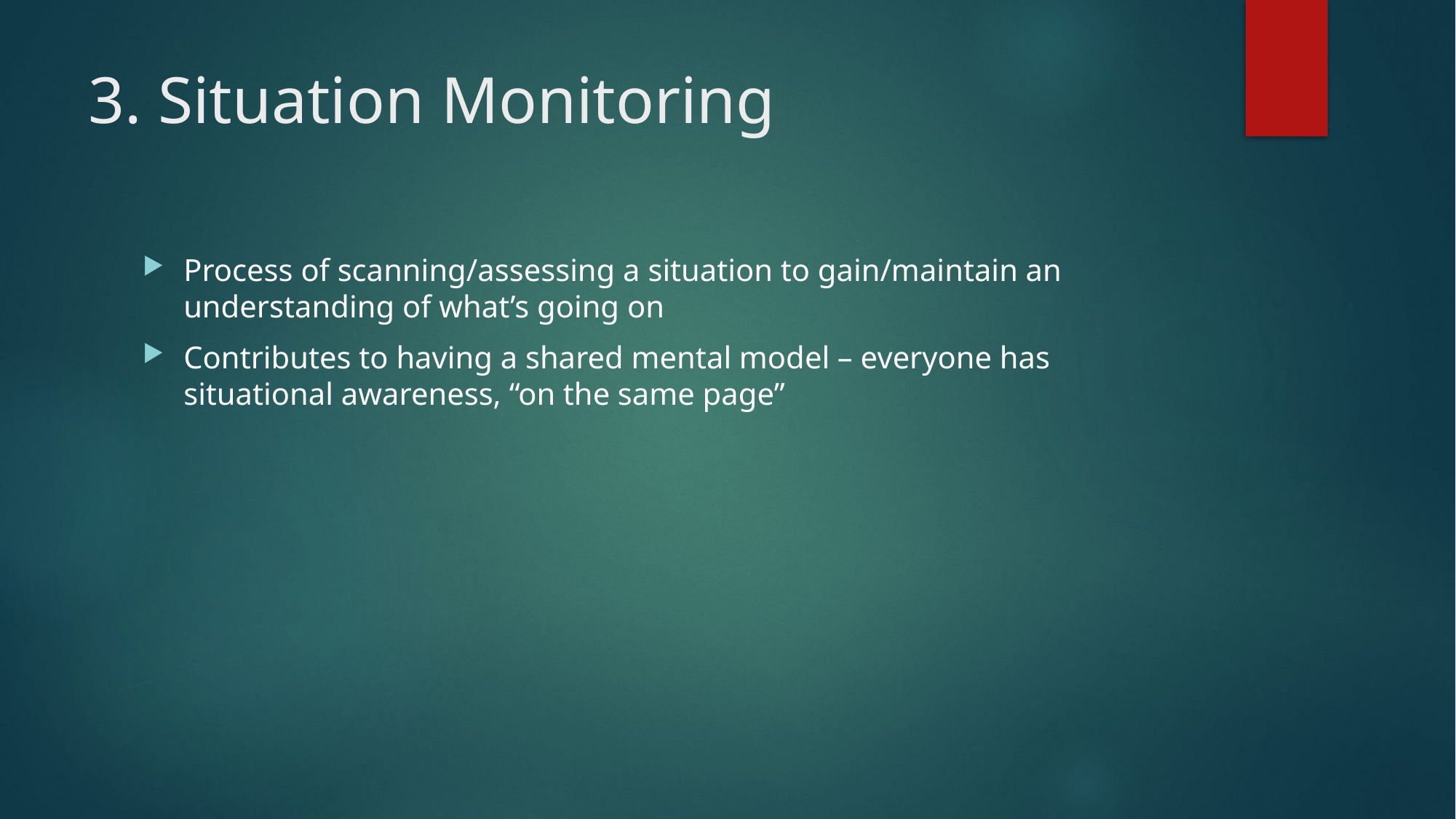

# 3. Situation Monitoring
Process of scanning/assessing a situation to gain/maintain an understanding of what’s going on
Contributes to having a shared mental model – everyone has situational awareness, “on the same page”

## Slide 21
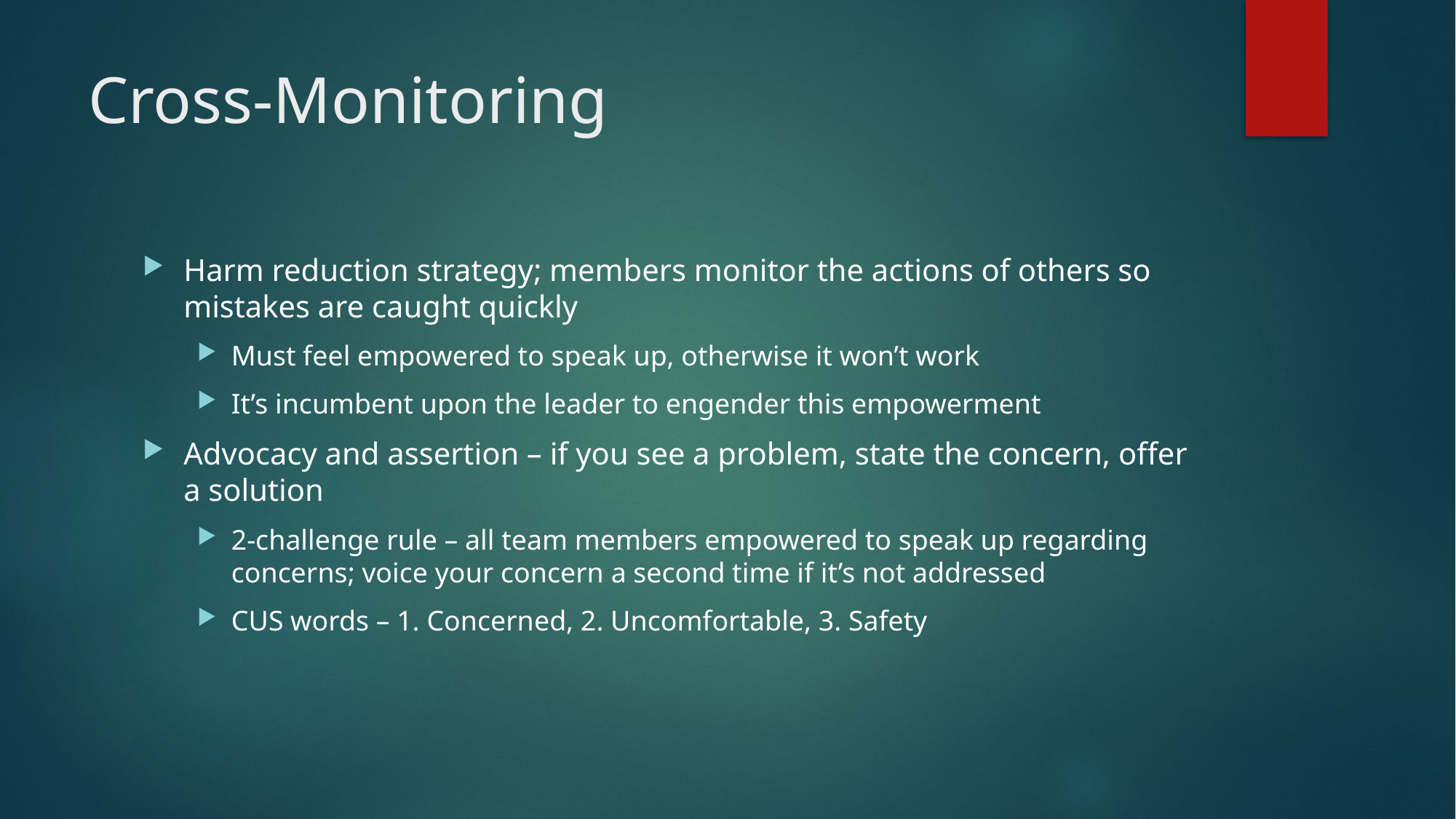

# Cross-Monitoring
Harm reduction strategy; members monitor the actions of others so mistakes are caught quickly
Must feel empowered to speak up, otherwise it won’t work
It’s incumbent upon the leader to engender this empowerment
Advocacy and assertion – if you see a problem, state the concern, offer a solution
2-challenge rule – all team members empowered to speak up regarding concerns; voice your concern a second time if it’s not addressed
CUS words – 1. Concerned, 2. Uncomfortable, 3. Safety

## Slide 22
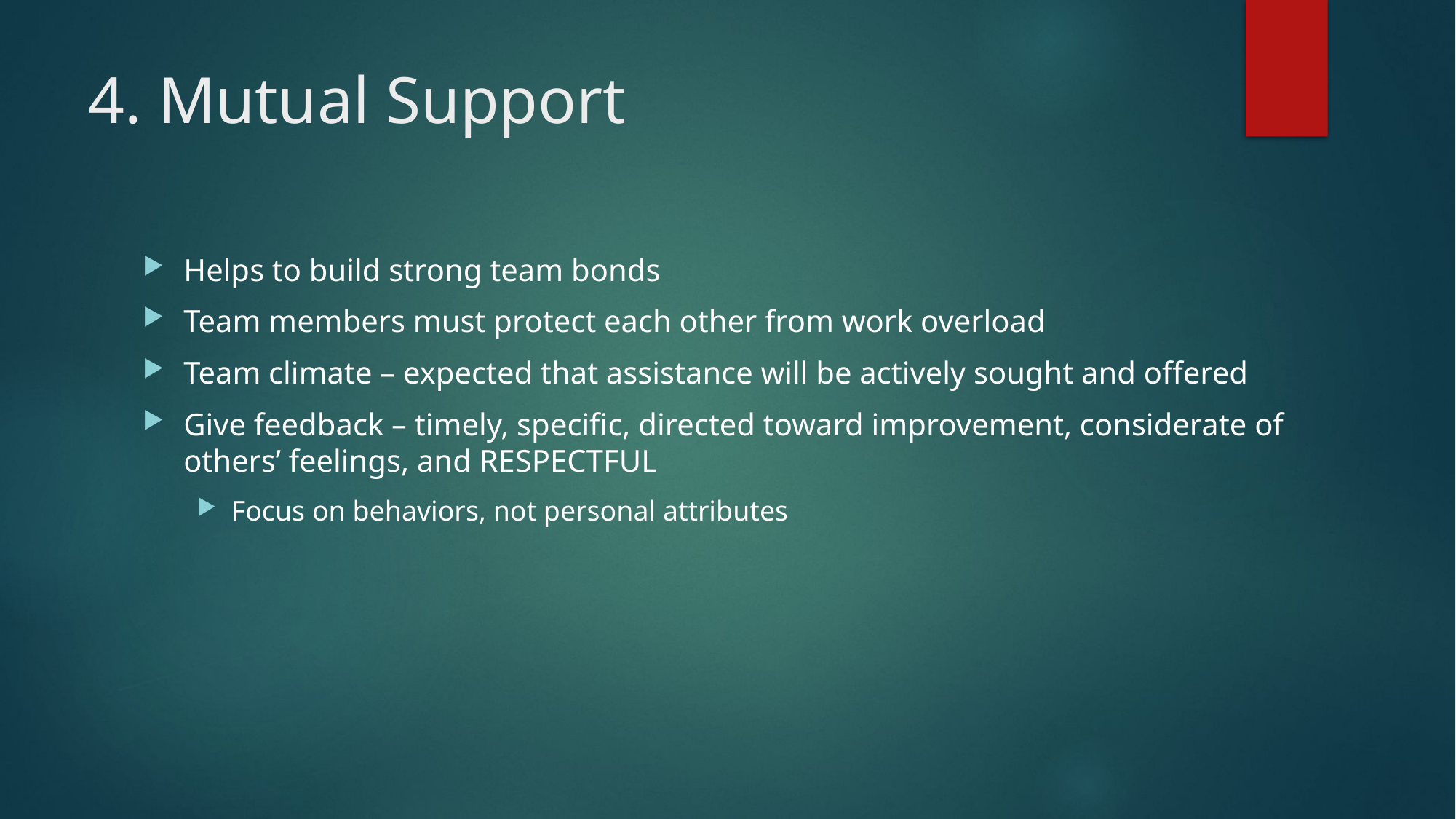

# 4. Mutual Support
Helps to build strong team bonds
Team members must protect each other from work overload
Team climate – expected that assistance will be actively sought and offered
Give feedback – timely, specific, directed toward improvement, considerate of others’ feelings, and RESPECTFUL
Focus on behaviors, not personal attributes

## Slide 23
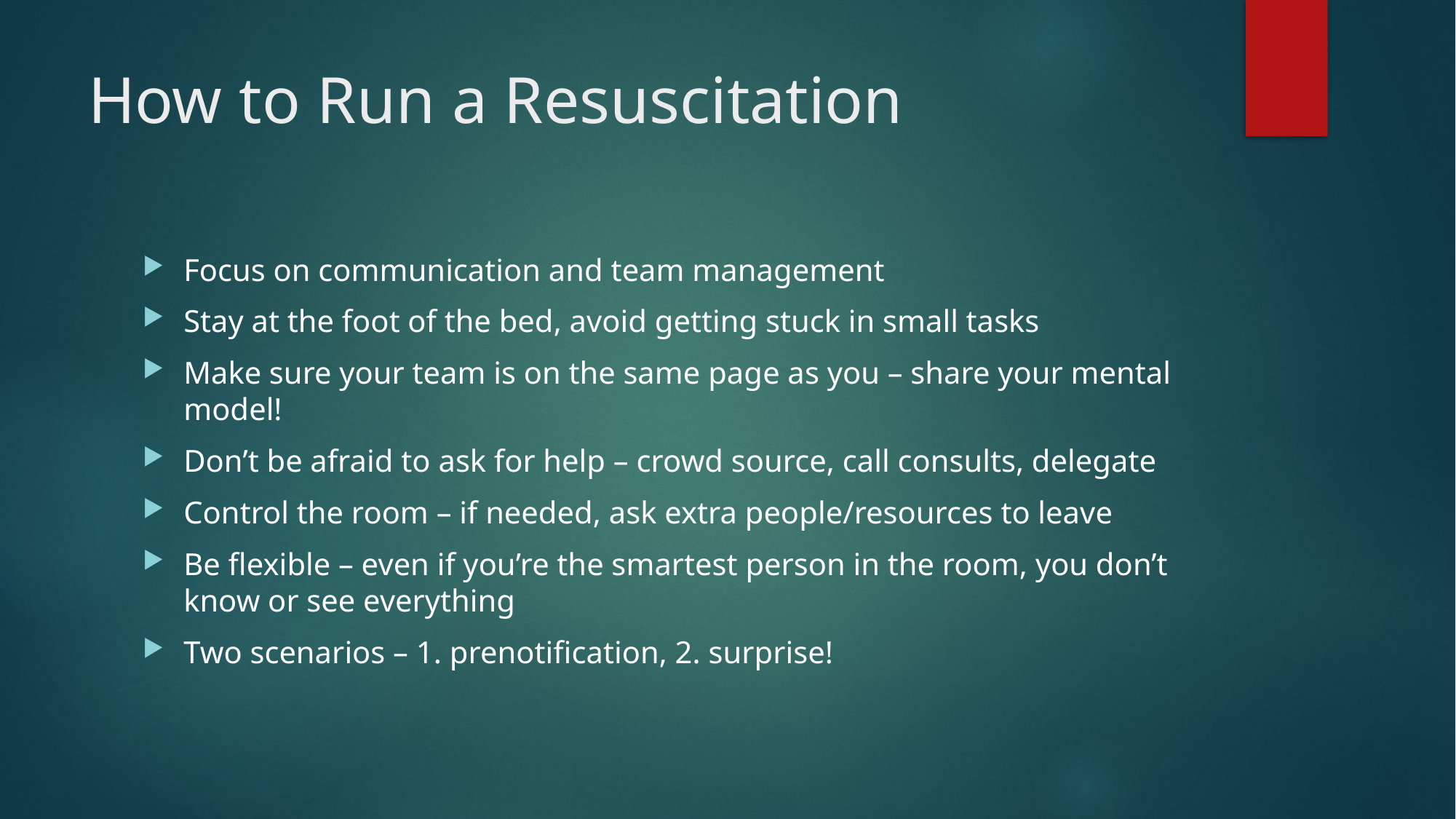

# How to Run a Resuscitation
Focus on communication and team management
Stay at the foot of the bed, avoid getting stuck in small tasks
Make sure your team is on the same page as you – share your mental model!
Don’t be afraid to ask for help – crowd source, call consults, delegate
Control the room – if needed, ask extra people/resources to leave
Be flexible – even if you’re the smartest person in the room, you don’t know or see everything
Two scenarios – 1. prenotification, 2. surprise!

## Slide 24
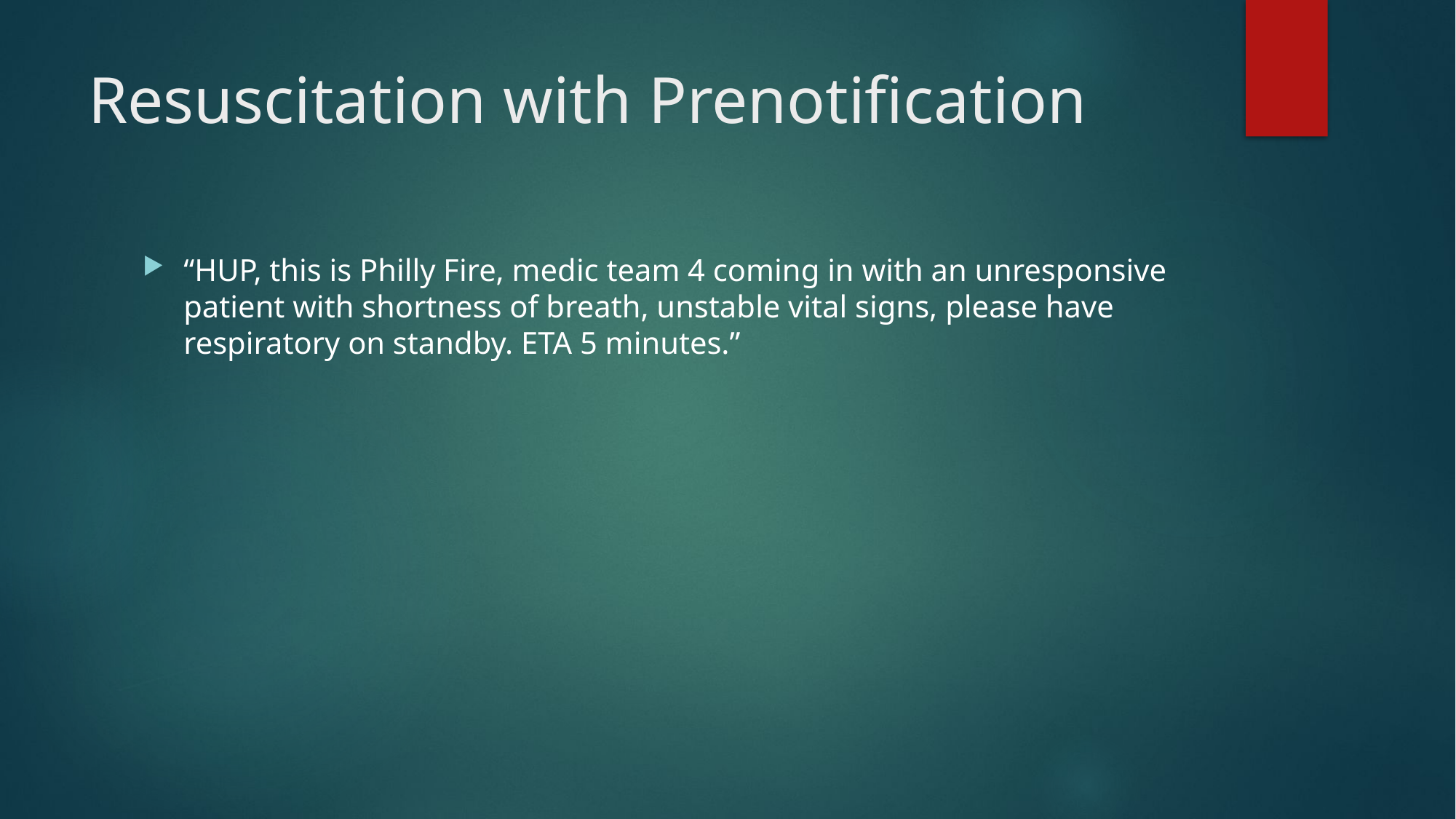

# Resuscitation with Prenotification
“HUP, this is Philly Fire, medic team 4 coming in with an unresponsive patient with shortness of breath, unstable vital signs, please have respiratory on standby. ETA 5 minutes.”

## Slide 25
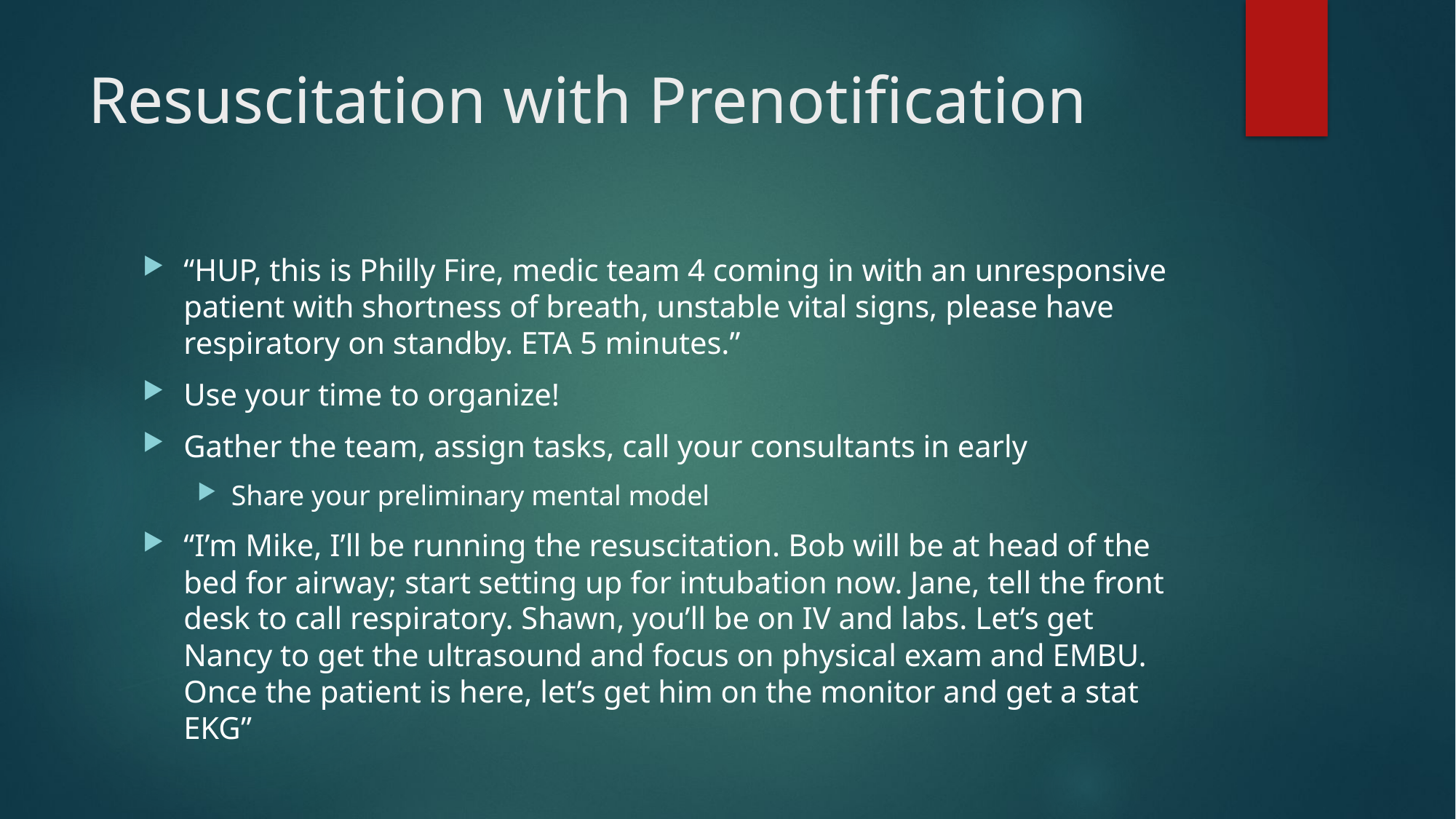

# Resuscitation with Prenotification
“HUP, this is Philly Fire, medic team 4 coming in with an unresponsive patient with shortness of breath, unstable vital signs, please have respiratory on standby. ETA 5 minutes.”
Use your time to organize!
Gather the team, assign tasks, call your consultants in early
Share your preliminary mental model
“I’m Mike, I’ll be running the resuscitation. Bob will be at head of the bed for airway; start setting up for intubation now. Jane, tell the front desk to call respiratory. Shawn, you’ll be on IV and labs. Let’s get Nancy to get the ultrasound and focus on physical exam and EMBU. Once the patient is here, let’s get him on the monitor and get a stat EKG”

## Slide 26
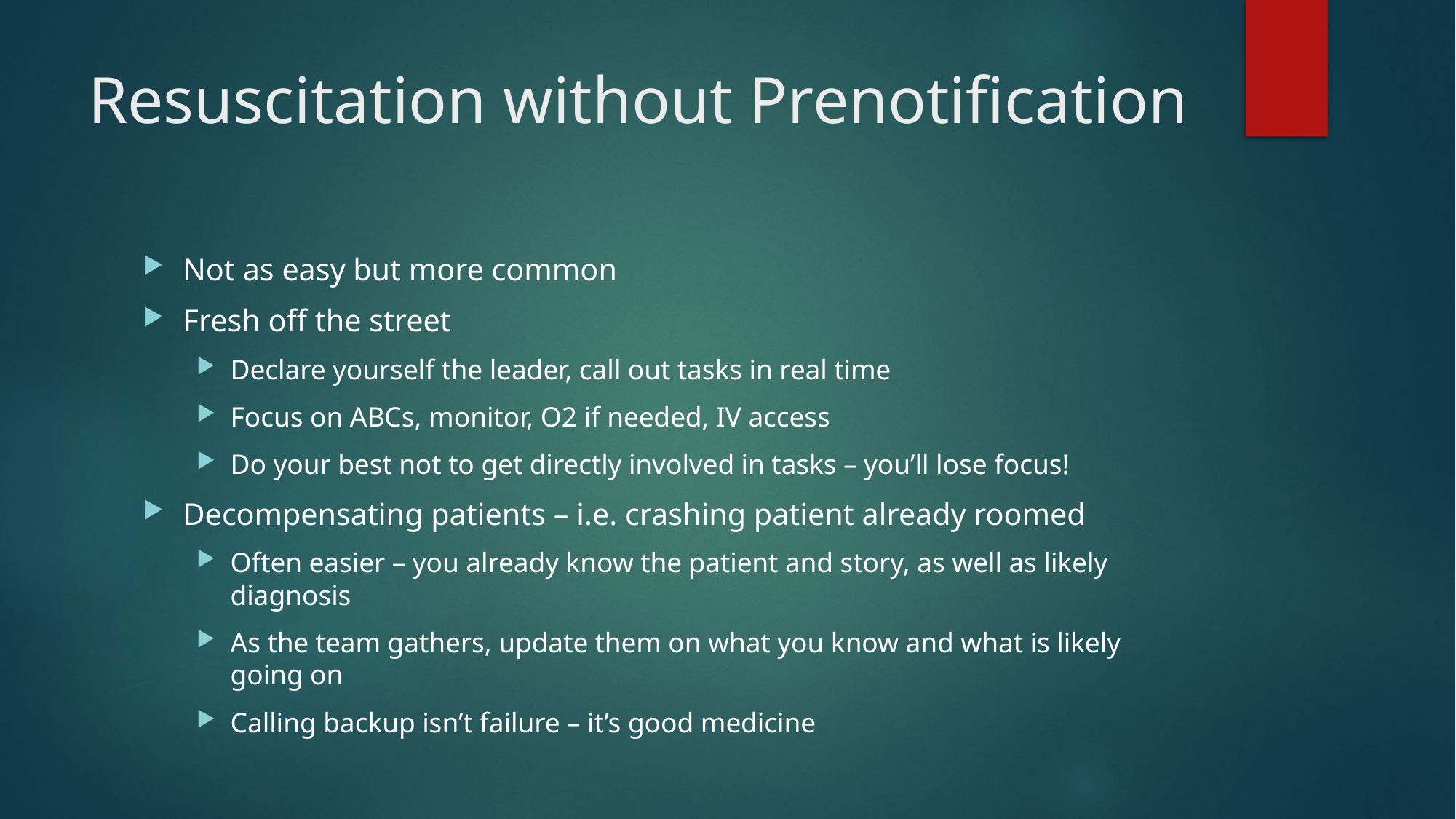

# Resuscitation without Prenotification
Not as easy but more common
Fresh off the street
Declare yourself the leader, call out tasks in real time
Focus on ABCs, monitor, O2 if needed, IV access
Do your best not to get directly involved in tasks – you’ll lose focus!
Decompensating patients – i.e. crashing patient already roomed
Often easier – you already know the patient and story, as well as likely diagnosis
As the team gathers, update them on what you know and what is likely going on
Calling backup isn’t failure – it’s good medicine

## Slide 27
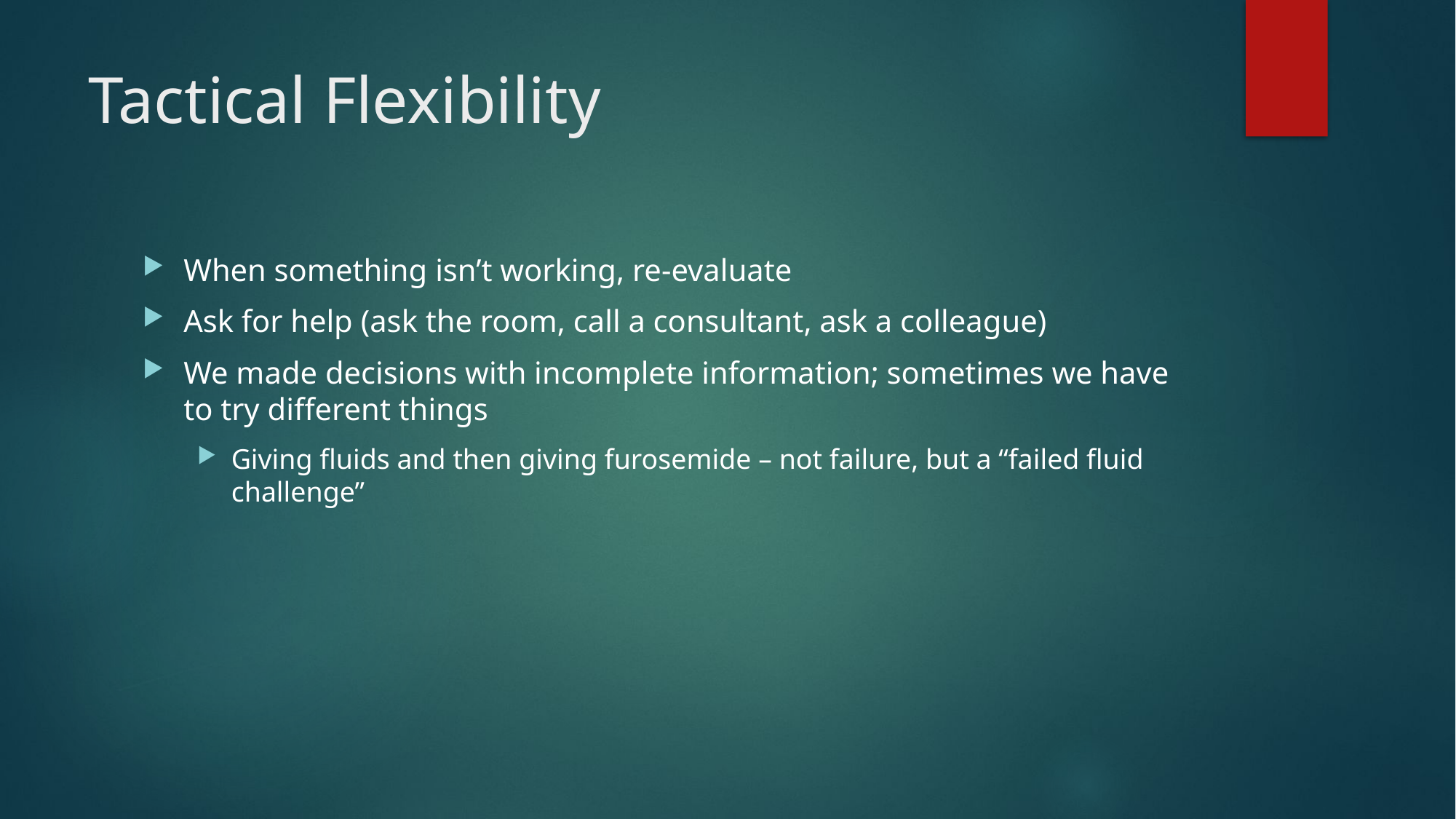

# Tactical Flexibility
When something isn’t working, re-evaluate
Ask for help (ask the room, call a consultant, ask a colleague)
We made decisions with incomplete information; sometimes we have to try different things
Giving fluids and then giving furosemide – not failure, but a “failed fluid challenge”

## Slide 28
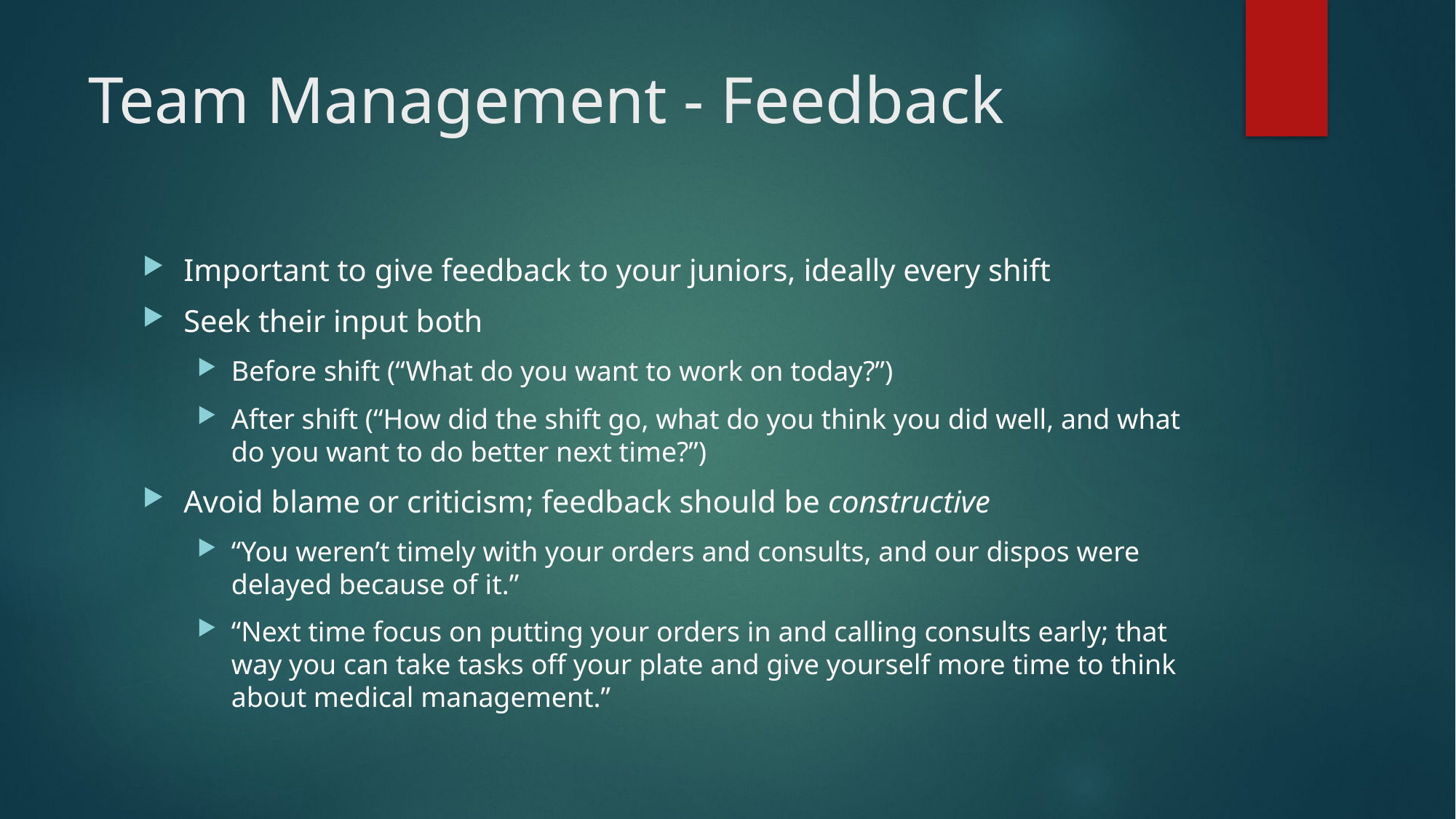

# Team Management - Feedback
Important to give feedback to your juniors, ideally every shift
Seek their input both
Before shift (“What do you want to work on today?”)
After shift (“How did the shift go, what do you think you did well, and what do you want to do better next time?”)
Avoid blame or criticism; feedback should be constructive
“You weren’t timely with your orders and consults, and our dispos were delayed because of it.”
“Next time focus on putting your orders in and calling consults early; that way you can take tasks off your plate and give yourself more time to think about medical management.”

## Slide 29
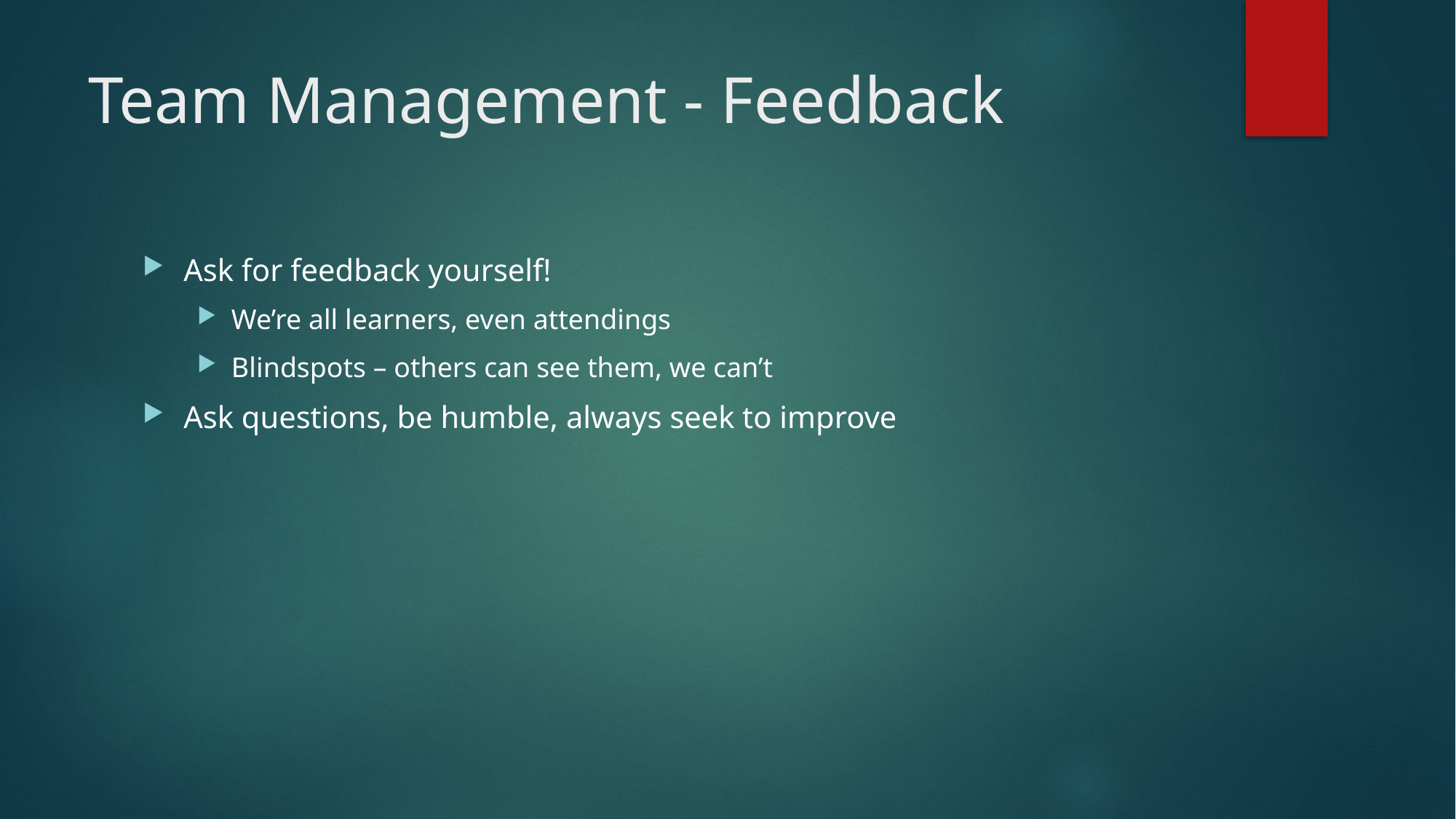

# Team Management - Feedback
Ask for feedback yourself!
We’re all learners, even attendings
Blindspots – others can see them, we can’t
Ask questions, be humble, always seek to improve

## Slide 30
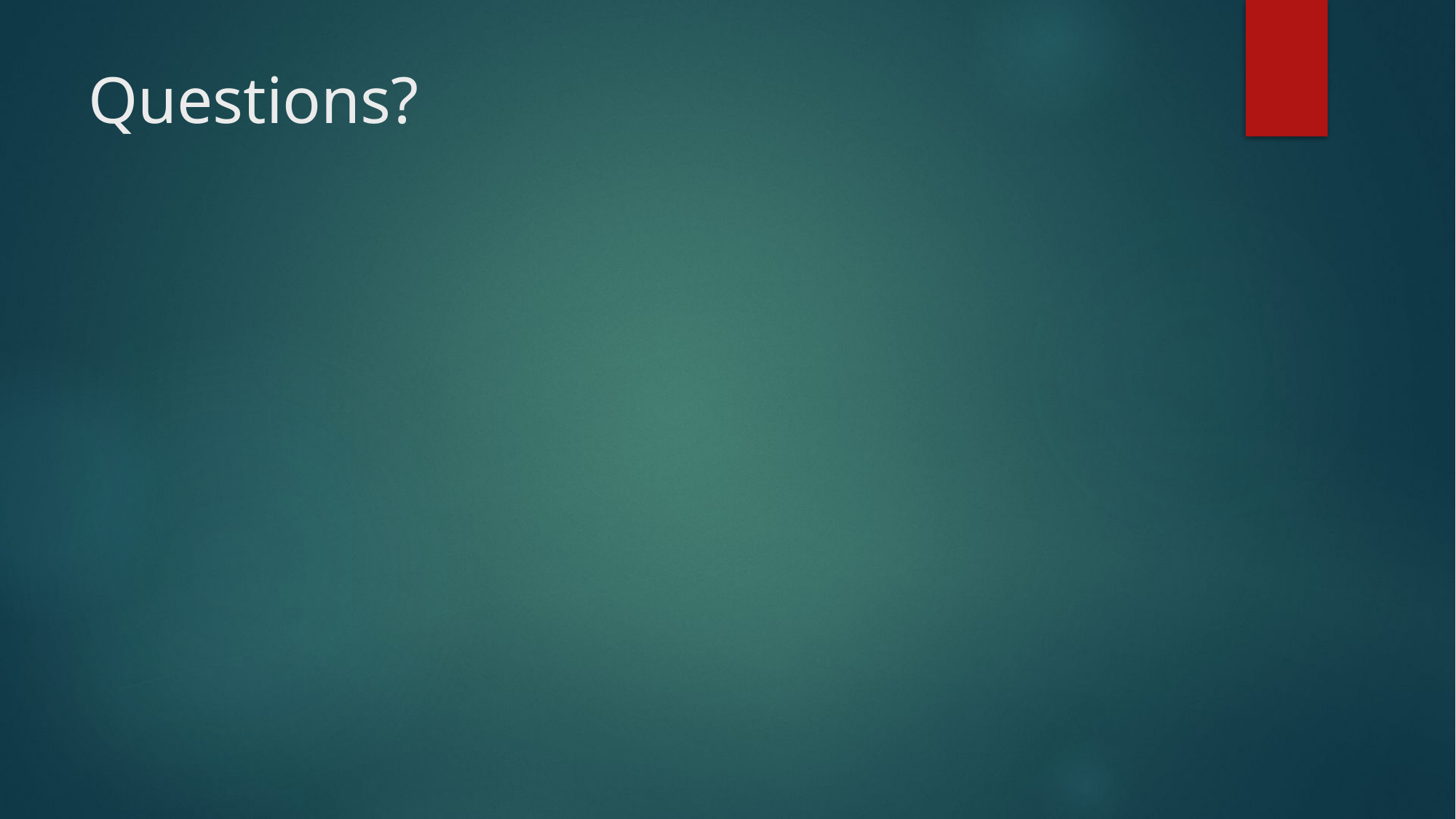

# Questions?

## Slide 31
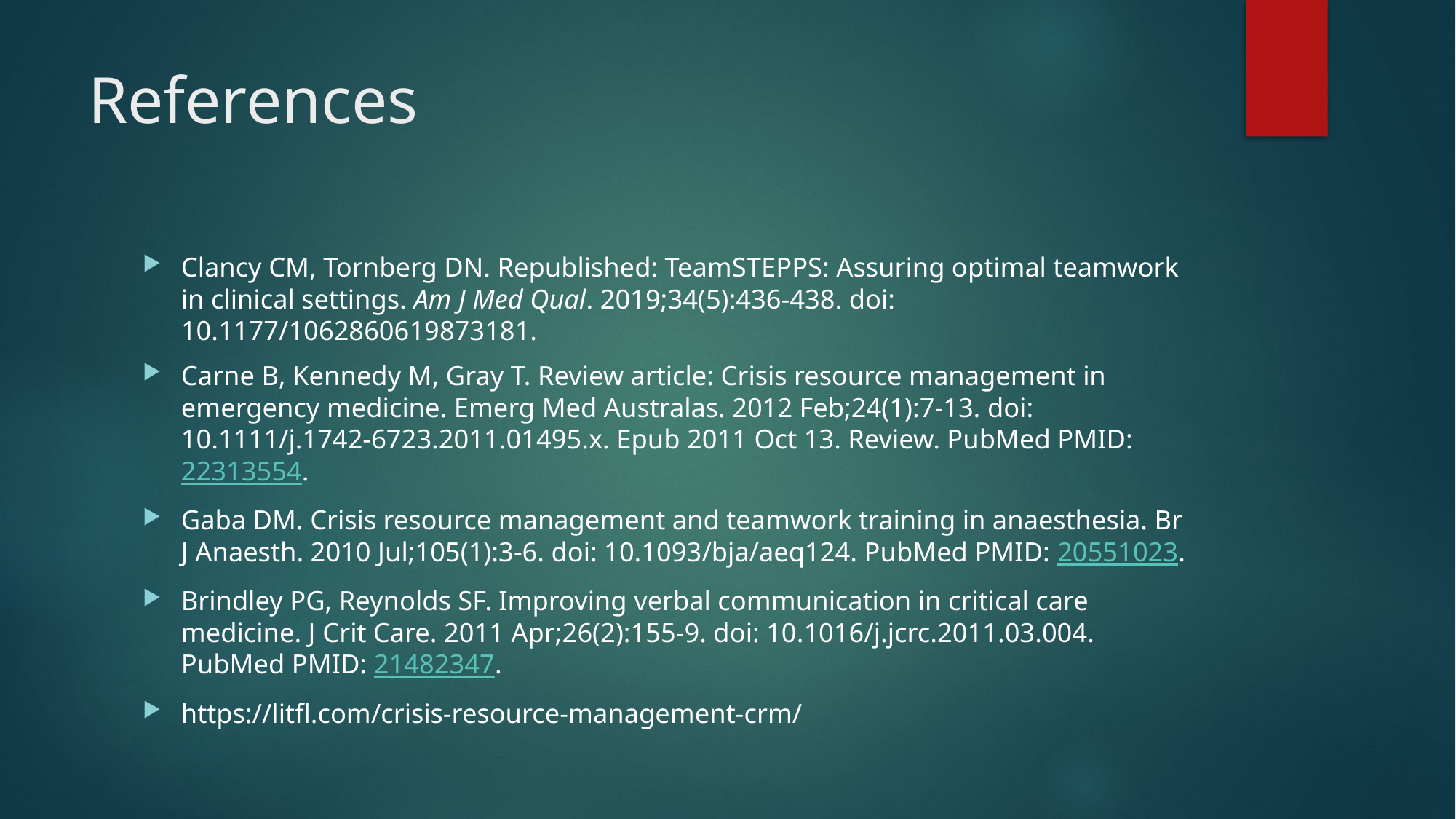

# References
Clancy CM, Tornberg DN. Republished: TeamSTEPPS: Assuring optimal teamwork in clinical settings. Am J Med Qual. 2019;34(5):436-438. doi: 10.1177/1062860619873181.
Carne B, Kennedy M, Gray T. Review article: Crisis resource management in emergency medicine. Emerg Med Australas. 2012 Feb;24(1):7-13. doi: 10.1111/j.1742-6723.2011.01495.x. Epub 2011 Oct 13. Review. PubMed PMID: 22313554.
Gaba DM. Crisis resource management and teamwork training in anaesthesia. Br J Anaesth. 2010 Jul;105(1):3-6. doi: 10.1093/bja/aeq124. PubMed PMID: 20551023.
Brindley PG, Reynolds SF. Improving verbal communication in critical care medicine. J Crit Care. 2011 Apr;26(2):155-9. doi: 10.1016/j.jcrc.2011.03.004. PubMed PMID: 21482347.
https://litfl.com/crisis-resource-management-crm/
