# Supplementary material for: Resuscitation Leadership Training: A Simulation Curriculum for Emergency Medicine Residents
Source: MedEdPORTAL. 2022 Oct 11;18:11278. doi: 10.15766/mep_2374-8265.11278 (PMC9550795; doi:10.15766/mep_2374-8265.11278)
Supplement: Supplementary file 1 — Sim Case - STEMI and VFib Arrest.docxCase Media and Labs - STEMI and VFib Arrest.pptxSim Case - Massive Pulmonary Embolism.docxCase Media and Labs - Massive PE.pptxSim Case - Wide Complex Tachycardia.docxCase Media and Labs - WCT.pptxSim Case - Missed Dialysis.docxCase Media and Labs - Missed Dialysis.pptxCAC - STEMI and VFib Arrest.docxCAC - Massive Pulmonary Embolism.docxCAC - Wide Complex Tachycardia.docxCAC - Missed Dialysis.docxCRM Presentation.pptxDebrief Handout.pdfSelect ACGME EM Milestones List.pptxOttawa GRS.docxResident Survey.docx [file mep_2374-8265.11278-s001.zip › I. CAC - STEMI and VFib Arrest.docx]

**Critical Actions Checklist: ST-Elevation Myocardial Infarction and Ventricular Fibrillation Arrest**

Leader __________________

1. Y/N Obtain an EKG
2. Y/N Place patient on supplemental oxygen
3. Y/N Place defibrillation pads on patient (before arrest)
4. Y/N Give aspirin
5. Y/N Give heparin bolus
6. Y/N Call catheterization lab
7. Y/N Defibrillate at 200J when goes into ventricular fibrillation
8. Y/N Intubate
9. Y/N Start an appropriate pressor (epinephrine, norepinephrine, or dopamine)
10. Y/N Give appropriate antidysrhythmic medication (amiodarone or lidocaine)
