# Supplementary material for: Resuscitation Leadership Training: A Simulation Curriculum for Emergency Medicine Residents
Source: MedEdPORTAL. 2022 Oct 11;18:11278. doi: 10.15766/mep_2374-8265.11278 (PMC9550795; doi:10.15766/mep_2374-8265.11278)
Supplement: Supplementary file 1 — Sim Case - STEMI and VFib Arrest.docxCase Media and Labs - STEMI and VFib Arrest.pptxSim Case - Massive Pulmonary Embolism.docxCase Media and Labs - Massive PE.pptxSim Case - Wide Complex Tachycardia.docxCase Media and Labs - WCT.pptxSim Case - Missed Dialysis.docxCase Media and Labs - Missed Dialysis.pptxCAC - STEMI and VFib Arrest.docxCAC - Massive Pulmonary Embolism.docxCAC - Wide Complex Tachycardia.docxCAC - Missed Dialysis.docxCRM Presentation.pptxDebrief Handout.pdfSelect ACGME EM Milestones List.pptxOttawa GRS.docxResident Survey.docx [file mep_2374-8265.11278-s001.zip › C. Sim Case - Massive Pulmonary Embolism.docx]

| **SIMULATION CASE TITLE:** Massive Pulmonary Embolism  **AUTHORS:** Michael Abboud, MD, MSEd  **LEARNER AUDIENCE:** PGY-2 Emergency Medicine Residents | | | | | | | |  |
| --- | --- | --- | --- | --- | --- | --- | --- | --- |
| **PATIENT NAME:** Sarah Smith  **PATIENT AGE:** 67 years old  **CHIEF COMPLAINT:** Syncope and chest pain  **PHYSICAL SETTING:** Emergency Department | | | | | | | |  |
|  | | | | | | | |  |
| **Brief narrative description of case** | | A 67-year-old female with a history of hypertension and hyperlipidemia presents with syncope and chest pain. She is tachycardic, hypotensive, and hypoxic upon arrival to the Emergency Department with exam and POCUS findings that the participants must recognize as consistent with a massive pulmonary embolism (PE). Participants must work together to effectively resuscitate this patient and administer thrombolytics before admitting her to the ICU. | | | | | |  |
| **Primary Learning Objectives** | | - Evaluate a patient with chest pain and syncope - Recognize signs of a massive PE on exam and POCUS - Demonstrate knowledge of appropriate management of a patient with a massive PE including hemodynamic support and administration of thrombolytics - Apply team leadership and communication skills to direct the resuscitation of an unstable patient | | | | | |  |
| **Critical Actions** | | 1. Obtain a venous blood gas and troponin 2. Obtain an EKG 3. Obtain a bedside echocardiogram 4. Call the Pulmonary Embolism Response Team* 5. Give IV fluids 6. Place the patient on supplemental oxygen 7. Go through the tPA checklist prior to giving tPA 8. Administer thrombolytics 9. Start an appropriate pressor 10. Admit to the ICU   *if implementing this simulation at a facility that does not have a PE Response Team or similar massive PE protocol, this critical action could instead involve consultation with Pulmonary and/or Critical Care for further guidance on massive PE management. | | | | | |  |
| **Learner Preparation or Prework** | | Learners should treat the mannequin and simulation as if it were a real patient scenario. | | | | | |  |
| Initial Presentation | | | | | | | |  |
| **Initial vital signs** | | BP 71/50, HR 137, T 97.9, RR 32, SpO2 81% on room air | | | | | |  |
| **Overall Setting and Appearance** | | The mannequin is lying in a stretcher in a hospital room. | | | | | |  |
| **Standardized Participants (and their roles in the room at case start**) | | A faculty member is present at the beginning of the case acting as the EMT. He or she gives report to the team, stating, “This is a 67-year-old woman who passed out at home then developed chest pain. She lost consciousness again in the ambulance. She had a thready pulse so we placed an IV and started fluids.”  A faculty member is present acting as the nurse, if the team requires prompting as listed below in “Instructor Notes.” | | | | | |  |
| **HPI** | | A 67-year-old female with history of hypertension and hyperlipidemia presents with syncope and chest pain. She was in her usual state of health when she suddenly felt lightheaded, sweaty, and developed chest pain that was described as sudden and crushing in the center of her chest. She lost consciousness, caught by her husband and lowered to the ground. May have twitched a couple times but no frank seizure activity. In the ambulance the patient lost consciousness again. EMS found a thready pulse and started fluids, thus far 200cc.  If asked about recent procedures or about leg swelling, patient then states that she had surgery on her knee last week.  When asked more about her symptoms, patient says her chest pain worsens with inspiration and she is also feeling short of breath and lightheaded. | | | | | |  |
| **Past Medical/Surgical History** | | **Medications** | | **Allergies** | | **Family History** | |  |
| Hypertension  Hyperlipidemia  Recent left knee arthroscopy for meniscal injury | | Lovastatin  Hydrochlorothiazide  Naproxen | | Penicillin | | Father had MI at age 65 | |  |
| **Physical Examination** | | | | | | | |  |
| **General** | | Distressed, diaphoretic, speaking 3-4 words at a time | | | | | |  |
| **HEENT** | | PERRL, normocephalic/atraumatic, mucus membranes moist | | | | | |  |
| **Neck** | | Supple, no tracheal deviation. | | | | | |  |
| **Lungs** | | Clear to auscultation bilaterally, moderate respiratory distress, tachypneic | | | | | |  |
| **Cardiovascular** | | Regular rhythm, tachycardic, +JVD | | | | | |  |
| **Abdomen** | | Soft, nontender, nondistended | | | | | |  |
| **Neurological** | | Alert, oriented x3, moving all extremities. Grossly non-focal neurologic exam. | | | | | |  |
| **Skin** | | Diaphoretic, warm/well-perfused. Left lower extremity has 1+ edema. | | | | | |  |
| **GU** | | Not done. | | | | | |  |
| **Psychiatric** | | Thought content normal, behavior appropriate. | | | | | |  |
| Instructor Notes - Changes and CASE Branch Points | | | | | | | | |
| **Intervention / Time point** | | | **Change in Case** | | **Additional Information** | | | |
| Give IV fluids | | | HR decreases to 133, BP stays about the same at 73/51 | |  | | | |
| Start pressor (besides phenylephrine) | | | BP increases to 89/51 | | If norepinephrine, epinephrine, or dobutamine, HR increases to 139. If vasopressin, HR 133. | | | |
| Start phenylephrine | | | Patient loses consciousness, HR 125, BP 65/49 | | Nurse calls attention to change in mental status by saying, “I think the patient just passed out.” | | | |
| Give supplemental oxygen | | | SpO2 increases | | If nasal canula, SpO2 87%. If nonrebreather mask, SpO2 93%. | | | |
| Intubate patient | | | SpO2 96% but BP decreases | |  | | | |
| Call Pulmonary Embolism Response Team | | | “Wow, this patient sounds sick. You should probably give thrombolytics. You can give fluids for BP support in the meantime. Please continue to stabilize the patient, and book her an ICU bed when more stable. We will see her in the ICU.” | |  | | | |
| Give tPA | | | BP increases to 97/60, HR decreases to 117 over next minute | |  | | | |

**Ideal Scenario Flow**

The learners enter the room to find a patient awake, in distress, and complaining of chest pain. One learner designates himself or herself as the team leader and assigns roles to the other team members (one person for airway, one person to act as bedside nurse, one person to obtain history/exam). The bedside learner immediately places the patient on the monitor, obtains IV access, and starts fluids. The history/exam learner obtains an appropriate history and performs a physical exam and relays pertinent information to the team leader, including the patient’s recent surgery. The airway learner places the patient on supplemental oxygen. The patient continues to be hypotensive, so the team starts pressors. The team also recognizes RV dilation on POCUS, and given the patient’s abnormal vital signs, recent surgery, and unilateral leg swelling, concludes that this is most likely a massive pulmonary embolism. Despite continued low SpO2 and respiratory distress, the team does not intubate the patient, as further increasing pulmonary pressures may cause worsening of the patient’s hemodynamics, which is communicated to the airway person. The team leader calls the Pulmonary Embolism Response Team, who recommend thrombolytics. Prior to administering tPA, the team goes through the tPA checklist with the patient. After administering tPA, the patient’s hemodynamics improve and she is admitted to the ICU. All learners use closed-loop communication throughout the scenario. The team leader demonstrates situation awareness and clearly allocates resources and tasks throughout the scenario.

**Anticipated Management Mistakes**

1. Uncertainty about indications for thrombolytics in massive pulmonary embolism: We encouraged the learners to give thrombolytics by having the consulting services suggest it, if the learners had not already discussed it. We reviewed the indications for thrombolytics and the definitions of massive vs. submassive PE during the debrief.
2. Uncertainty about vasopressor choice in acute pulmonary hypertension: We reviewed the indications and contraindications for common vasopressors in the setting of massive PE.
3. Intubating the patient and causing further hypotension: We reviewed the effects of positive-pressure intubation in patients who are preload-dependent during the debrief.
4. Failure of the team leader to identify roles for the team members at the beginning of the case, used closed-loop communication, clearly allocate resources and tasks, and/or demonstrate situational awareness during the case: We reviewed the performance of the team leader and the team dynamics during the debrief after each case, including faculty observations regarding the application of CRM and TeamSTEPPS principles.
