# Supplementary material for: Resuscitation Leadership Training: A Simulation Curriculum for Emergency Medicine Residents
Source: MedEdPORTAL. 2022 Oct 11;18:11278. doi: 10.15766/mep_2374-8265.11278 (PMC9550795; doi:10.15766/mep_2374-8265.11278)
Supplement: Supplementary file 1 — Sim Case - STEMI and VFib Arrest.docxCase Media and Labs - STEMI and VFib Arrest.pptxSim Case - Massive Pulmonary Embolism.docxCase Media and Labs - Massive PE.pptxSim Case - Wide Complex Tachycardia.docxCase Media and Labs - WCT.pptxSim Case - Missed Dialysis.docxCase Media and Labs - Missed Dialysis.pptxCAC - STEMI and VFib Arrest.docxCAC - Massive Pulmonary Embolism.docxCAC - Wide Complex Tachycardia.docxCAC - Missed Dialysis.docxCRM Presentation.pptxDebrief Handout.pdfSelect ACGME EM Milestones List.pptxOttawa GRS.docxResident Survey.docx [file mep_2374-8265.11278-s001.zip › E. Sim Case - Wide Complex Tachycardia.docx]

| **SIMULATION CASE TITLE:** Wide Complex Tachycardia  **AUTHORS:** Michael Abboud, MD, MSEd  **LEARNER AUDIENCE:** PGY-2 Emergency Medicine Residents | | | | | | | |  |
| --- | --- | --- | --- | --- | --- | --- | --- | --- |
| **PATIENT NAME:** Fred Smith  **PATIENT AGE:** 58 years old  **CHIEF COMPLAINT:** Lightheadedness and nausea  **PHYSICAL SETTING:** Emergency Department | | | | | | | |  |
|  | | | | | | | |  |
| **Brief narrative description of case** | | A 58-year-old male with a history of hypertension, hyperlipidemia, coronary artery disease s/p LAD stent, congestive heart failure with an EF of 55% presents with lightheadedness and nausea. He is found to have a wide complex tachycardia but initial blood pressure is normal, so the team must give an antidysrhythmic medication. His rhythm does not change with the first medication, but he becomes hypotensive and more symptomatic, requiring synchronized cardioversion to sinus rhythm. He continues to have runs of non-sustained ventricular tachycardia, necessitating administration of a second antidysrhythmic medication. Participants must work together to effectively resuscitate this patient and administer appropriate medications before admitting him to the ICU. | | | | | |  |
| **Primary Learning Objectives** | | - Evaluate a patient with an arrhythmia - Identify a wide complex tachycardia - Demonstrate knowledge of management of a stable vs. unstable patient with wide complex tachycardia - Apply pharmacologic knowledge of antidysrhythmic medications to this patient case - Apply team leadership and communication skills to direct the resuscitation of an unstable patient | | | | | |  |
| **Critical Actions** | | 1. Recognize a wide complex tachycardia on EKG 2. Place pads on the patient immediately 3. Give an appropriate antidysrhythmic medication 4. Give a second antidysrhythmic medication 5. Administer synchronized cardioversion when the patient becomes unstable 6. Explain the cardioversion to the patient prior to shocking 7. Send labs including troponin and electrolytes 8. Monitor appropriately during cardioversion 9. Repeat EKG after cardioversion 10. Call and admit to cardiology | | | | | |  |
| **Learner Preparation or Prework** | | Learners should treat the mannequin and simulation as if it were a real patient scenario. | | | | | |  |
| Initial Presentation | | | | | | | |  |
| **Initial vital signs** | | BP 122/78, HR 164, T 98.8, RR 18, SpO2 98% on room air | | | | | |  |
| **Overall Setting and Appearance** | | The mannequin is lying in a stretcher in a hospital room. | | | | | |  |
| **Standardized Participants (and their roles in the room at case start**) | | None. | | | | | |  |
| **HPI** | | A 58-year-old male with history of hypertension, hyperlipidemia, coronary artery disease s/p PCI to LAD 2018, CHF with EF 55%, presents to the ED with nausea and lightheadedness for 45 minutes. He was at home watching television when he started to feel nauseated and lightheaded. He took some Tums with minimal relief. Currently he denies any chest pain or shortness of breath. He has never had symptoms like this before. | | | | | |  |
| **Past Medical/Surgical History** | | **Medications** | | **Allergies** | | **Family History** | |  |
| Hypertension  Hyperlipidemia  Congestive heart failure  Coronary artery disease s/p PCI to LAD | | Aspirin  Plavix  Metoprolol  Simvastatin  Lisinopril | | None | | Multiple family members with heart disease | |  |
| **Physical Examination** | | | | | | | |  |
| **General** | | Anxious but alert, no acute distress | | | | | |  |
| **HEENT** | | PERRL, normocephalic/atraumatic, mucus membranes moist | | | | | |  |
| **Neck** | | Supple, no tracheal deviation. | | | | | |  |
| **Lungs** | | Clear to auscultation bilaterally, moderate respiratory distress, tachypneic | | | | | |  |
| **Cardiovascular** | | Regular rhythm, tachycardic, no JVD | | | | | |  |
| **Abdomen** | | Soft, nontender, nondistended | | | | | |  |
| **Neurological** | | Alert, oriented x3, moving all extremities. Grossly non-focal neurologic exam. | | | | | |  |
| **Skin** | | Diaphoretic, warm/well-perfused. | | | | | |  |
| **GU** | | Not done. | | | | | |  |
| **Psychiatric** | | Thought content normal, behavior appropriate. | | | | | |  |
| Instructor Notes - Changes and CASE Branch Points | | | | | | | | |
| **Intervention / Time point** | | | **Change in Case** | | **Additional Information** | | | |
| Attempt to cardiovert before trying medications | | | “Please don’t shock me! Can’t you do anything else?” | | Patient continues to refuse cardioversion initially until team tries medications | | | |
| Give IV fluids | | | No change | |  | | | |
| Give antidysrhythmic medication (amiodarone, procainamide, lidocaine, or beta-blockers) | | | HR slows to 155, BP unchanged | |  | | | |
| After 1 minute | | | Patient complains of lightheadedness and chest discomfort, BP decreases to 91/51 | |  | | | |
| Cardiovert with >100J | | | HR 85 in sinus rhythm, BP 131/84, has frequent runs of NSVT | | If not synchronized, patient goes into ventricular fibrillation; when shocked again, goes back to sinus rhythm. If shock < 100J, no rhythm change. | | | |
| If giving sedation meds for cardioversion but no supplemental oxygen | | | SpO2 drops to 88%, improves after giving supplemental oxygen | | If no sedation meds, patient screams when shocked. | | | |
| Give second antidysrhythmic medication for continued runs of NSVT (amiodarone, procainamide, lidocaine, or beta-blockers) | | | Less frequent runs of NSVT, HR otherwise still in 80s and sinus rhythm, BP stable | |  | | | |
| Call cardiology | | | “You can try antidysrhythmic medications, but if the patient is unstable, you should cardiovert.” | |  | | | |

**Ideal Scenario Flow**

The learners enter the room to find a patient awake and complaining of lightheadedness and nausea. One learner designates himself or herself as the team leader and assigns roles to the other team members (one person for airway, one person to act as bedside nurse, one person to obtain history/exam). The bedside learner immediately places the patient on the monitor and obtains IV access while the team leader asks for labs and an EKG. The history/exam learner obtains an appropriate history and performs a physical exam and relays pertinent information to the team leader. The team immediately recognizes a wide complex tachycardia on the monitor and on EKG, so places pads on the patient. The airway learner places the patient on supplemental oxygen. The patient is normotensive, so an antidysrhythmic medication is started, which decreases the heart rate but does not change the rhythm. The team leader also calls cardiology, who recommend medical therapy unless he becomes unstable, at which point they would recommend cardioversion. After a minute, the patient complains of worsening lightheadedness and chest pain and becomes hypotensive. The team informs the patient that he must undergo synchronized cardioversion as he has become unstable, then gives him sedation meds prior to synchronized cardioversion of >100J. Following the cardioversion, the patient’s heart rate is 85 in sinus rhythm with BP 131/84. He continues to have runs of non-sustained ventricular tachycardia, so a second antidysrhythmic medication is started before he is admitted to the ICU. All learners use closed-loop communication throughout the scenario. The team leader demonstrates situation awareness and clearly allocates resources and tasks throughout the scenario.

**Anticipated Management Mistakes**

1. Uncertainty about antidysrhythmic medications: We encouraged use of an antidysrhythmic medication first by having the patient refuse shocks. The patient purposely has frequent runs of non-sustained ventricular tachycardia after defibrillation to encourage the team to give a second antidysrhythmic medication. We reviewed the types of antiarrhythmic medications and their indications during the debrief.
2. Delivering an unsynchronized shock: We reviewed importance and indications for synchronized vs unsynchronized shocks during the debrief. We also introduced the monitor and gave a brief demonstration of how to use it prior to the simulation cases.
3. Failure of the team leader to identify roles for the team members at the beginning of the case, used closed-loop communication, clearly allocate resources and tasks, and/or demonstrate situational awareness during the case: We reviewed the performance of the team leader and the team dynamics during the debrief after each case, including faculty observations regarding the application of CRM and TeamSTEPPS principles.
