# Supplementary material for: Resuscitation Leadership Training: A Simulation Curriculum for Emergency Medicine Residents
Source: MedEdPORTAL. 2022 Oct 11;18:11278. doi: 10.15766/mep_2374-8265.11278 (PMC9550795; doi:10.15766/mep_2374-8265.11278)
Supplement: Supplementary file 1 — Sim Case - STEMI and VFib Arrest.docxCase Media and Labs - STEMI and VFib Arrest.pptxSim Case - Massive Pulmonary Embolism.docxCase Media and Labs - Massive PE.pptxSim Case - Wide Complex Tachycardia.docxCase Media and Labs - WCT.pptxSim Case - Missed Dialysis.docxCase Media and Labs - Missed Dialysis.pptxCAC - STEMI and VFib Arrest.docxCAC - Massive Pulmonary Embolism.docxCAC - Wide Complex Tachycardia.docxCAC - Missed Dialysis.docxCRM Presentation.pptxDebrief Handout.pdfSelect ACGME EM Milestones List.pptxOttawa GRS.docxResident Survey.docx [file mep_2374-8265.11278-s001.zip › G. Sim Case - Missed Dialysis.docx]

| **SIMULATION CASE TITLE:** Missed Dialysis  **AUTHORS:** Michael Abboud, MD, MSEd  **LEARNER AUDIENCE:** PGY-2 Emergency Medicine Residents | | | | | | | |  |
| --- | --- | --- | --- | --- | --- | --- | --- | --- |
| **PATIENT NAME:** George Smith  **PATIENT AGE:** 68 years old  **CHIEF COMPLAINT:** Shortness of breath  **PHYSICAL SETTING:** Emergency Department | | | | | | | |  |
|  | | | | | | | |  |
| **Brief narrative description of case** | | A 68-year-old male with a history of hypertension, diabetes, atrial fibrillation, coronary artery disease, end-stage renal failure on hemodialysis, and COPD presents with shortness of breath. He is in respiratory distress, hypertensive, and hypoxic with signs of volume overload on exam and imaging. He is started on non-invasive positive pressure ventilation and a nitroglycerin infusion with improvement in both his blood pressure and work of breathing. The team needs to identify an elevated potassium and widened QRS on EKG then give medications to treat hyperkalemia. He is admitted to the ICU and started on emergent hemodialysis. Participants must work together to effectively resuscitate this patient and administer appropriate medications before admitting him to the ICU. | | | | | |  |
| **Primary Learning Objectives** | | - Identify and evaluate a patient with pulmonary edema - Identify a widened QRS in the setting of hyperkalemia - Demonstrate knowledge of the management of hyperkalemia, severe pulmonary edema, and hypertension - Apply team leadership and communication skills to direct the resuscitation of an unstable patient | | | | | |  |
| **Critical Actions** | | 1. Obtain an EKG 2. Start positive pressure ventilation (BiPAP, CPAP, or intubation) 3. Give nitroglycerin (sublingual or infusion) 4. Send labs including potassium and troponin 5. Obtain lung imaging (POCUS and/or CXR) 6. Give calcium gluconate IV 7. Give temporizing hyperkalemia medications (albuterol, insulin, and/or bicarb) 8. Give potassium eliminating medications (furosemide or kayexalate) 9. Call nephrology for emergent hemodialysis 10. Admit to the ICU | | | | | |  |
| **Learner Preparation or Prework** | | Learners should treat the mannequin and simulation as if it were a real patient scenario. | | | | | |  |
| Initial Presentation | | | | | | | |  |
| **Initial vital signs** | | T 98.8, HR 127, BP 245/121, RR 34, SpO2 85% on 2L nasal canula | | | | | |  |
| **Overall Setting and Appearance** | | The mannequin is lying in a stretcher in a hospital room. There is a dialysis fistula in the left upper extremity. | | | | | |  |
| **Standardized Participants (and their roles in the room at case start**) | | None. | | | | | |  |
| **HPI** | | A 68-year-old male with a history of coronary artery disease, hypertension, diabetes, atrial fibrillation on coumadin, COPD, end stage renal disease on hemodialysis presents to the ED with shortness of breath for the past day. The patient’s wife called EMS, who found the patient to be in respiratory distress gasping for air.  If asked, the patient missed his last two dialysis sessions. He does not have chest pain. | | | | | |  |
| **Past Medical/Surgical History** | | **Medications** | | **Allergies** | | **Family History** | |  |
| Hypertension  Hyperlipidemia  Diabetes mellitus  Atrial fibrillation  COPD  Coronary artery disease s/p PCI x2  End stage renal disease | | Aspirin  Coumadin  Lisinopril  Amlodipine  Metoprolol  Insulin  Atorvastatin  Flovent | | Shellfish | | Multiple family members with heart disease and diabetes | |  |
| **Physical Examination** | | | | | | | |  |
| **General** | | Speaking one word at a time, in distress | | | | | |  |
| **HEENT** | | PERRL, normocephalic/atraumatic, mucus membranes moist | | | | | |  |
| **Neck** | | Supple, no tracheal deviation | | | | | |  |
| **Lungs** | | Rales throughout, in severe respiratory distress | | | | | |  |
| **Cardiovascular** | | Regular rhythm, tachycardic, +JVD | | | | | |  |
| **Abdomen** | | Soft, nontender, nondistended | | | | | |  |
| **Neurological** | | Alert, oriented x3, moving all extremities. Grossly non-focal neurologic exam | | | | | |  |
| **Skin** | | Diaphoretic, warm/well-perfused. LUE fistula present with palpable thrill | | | | | |  |
| **GU** | | Not done | | | | | |  |
| **Psychiatric** | | Thought content normal, behavior appropriate | | | | | |  |
| Instructor Notes - Changes and CASE Branch Points | | | | | | | | |
| **Intervention / Time point** | | | **Change in Case** | | **Additional Information** | | | |
| If placed on non-rebreather mask | | | SpO2 90%, RR 34 | |  | | | |
| If placed on BiPAP or CPAP | | | SpO2 95%, RR 28 | |  | | | |
| If given sublingual nitroglycerin | | | BP 205/109, no change in respiratory status | |  | | | |
| If started on nitro infusion | | | BP and RR decrease   - Rate 100mcg/hr: BP 200/100, RR 28 - Rate 200mcg/hr: BP 180/90, RR 24 - Rate 300mcg/hr: BP 150/80, RR 22 - Rate 400 mcg/hr: BP 130/80, RR 20 | |  | | | |
| If given calcium gluconate IV | | | QRS narrows | |  | | | |
| If no calcium gluconate is given after 10 minutes | | | Patient codes | | Team then must run ACLS. ROSC achieved if given calcium gluconate in first two rounds, otherwise scenario ends. | | | |
| Call nephrology | | | “We will set up emergent hemodialysis and meet you in the ICU” | |  | | | |

**Ideal Scenario Flow**

The learners enter the room to find a patient in severe respiratory distress. One learner designates himself or herself as the team leader and assigns roles to the other team members (one person for airway, one person to act as bedside nurse, one person to obtain history/exam). The bedside learner immediately places the patient on the monitor and obtains IV access while the team leader asks for labs and an EKG. The airway learner places the patient on a nonrebreather mask with only slight improvement in the patient’s SpO2. The history/exam learner obtains an appropriate history and performs a physical exam and relays pertinent information to the team leader, including missed dialysis sessions and rales on exam. The team recognizes the patient’s elevated blood pressure and exam findings are consistent with volume overload and pulmonary edema, so they start BiPAP and a nitroglycerin infusion with subsequent improvement in the patient’s vitals. The team leader also calls renal to start emergent hemodialysis and admits the patient to the ICU. All learners use closed-loop communication throughout the scenario. The team leader demonstrates situation awareness and clearly allocates resources and tasks throughout the scenario.

**Anticipated Management Mistakes**

1. Not giving hyperkalemia medications in time: We provided an EKG with purposely widened QRS as a result of the hyperkalemia to further push learners towards treating it. We reviewed the dangers of hyperkalemia and the treatments for it during the debrief.
2. Inappropriate treatment of flash pulmonary edema: The patient requires both immediate management (positive pressure, blood pressure control) as well as rapid consultation for definitive intervention (renal consult for hemodialysis). Residents may overlook consultation and only manage the patient with emergency department interventions.
3. Failure of the team leader to identify roles for the team members at the beginning of the case, used closed-loop communication, clearly allocate resources and tasks, and/or demonstrate situational awareness during the case: We reviewed the performance of the team leader and the team dynamics during the debrief after each case, including faculty observations regarding the application of CRM and TeamSTEPPS principles.
