# Supplementary material for: Resuscitation Leadership Training: A Simulation Curriculum for Emergency Medicine Residents
Source: MedEdPORTAL. 2022 Oct 11;18:11278. doi: 10.15766/mep_2374-8265.11278 (PMC9550795; doi:10.15766/mep_2374-8265.11278)
Supplement: Supplementary file 1 — Sim Case - STEMI and VFib Arrest.docxCase Media and Labs - STEMI and VFib Arrest.pptxSim Case - Massive Pulmonary Embolism.docxCase Media and Labs - Massive PE.pptxSim Case - Wide Complex Tachycardia.docxCase Media and Labs - WCT.pptxSim Case - Missed Dialysis.docxCase Media and Labs - Missed Dialysis.pptxCAC - STEMI and VFib Arrest.docxCAC - Massive Pulmonary Embolism.docxCAC - Wide Complex Tachycardia.docxCAC - Missed Dialysis.docxCRM Presentation.pptxDebrief Handout.pdfSelect ACGME EM Milestones List.pptxOttawa GRS.docxResident Survey.docx [file mep_2374-8265.11278-s001.zip › N. Debrief Handout.pdf]

Appendix N. Debrief Teaching Pearls Handout

Technical Skills: Medical knowledge, procedural skills, physical exam skills

Non-Technical Skills (NOTECH): Communication, teamwork, leadership, task delegation, ID bottlenecks

Crisis Resource Management (CRM): Combining medical knowledge with teamwork and efficient resource utilization to deliver effective care in complex situations

Key Principles:

- Know the environment
- Pre-brief when possible
- Mobilize resources early

Leadership:

- Foot of bed, hands-off
- Organize team, assign tasks appropriately
- Global Assessment:
  - Shared Mental Model***
  - Reality Check

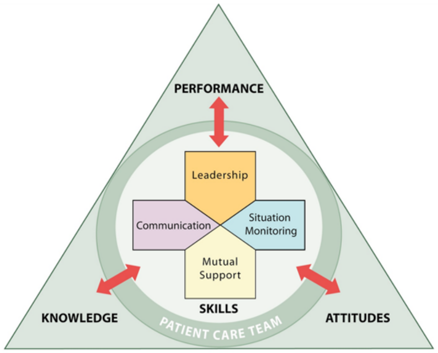

Communication:

- Closed Loop Communication
- Leader ←→ Follower, not Follower ←→ Follower
- Non-judgmental
- Crowd control

Monitoring & Support:

- Advocacy and Assertion
- 2 Challenge Rule
- Team bonding, positive atmosphere, no judgment

STEMI

- ABCs
  - Oxygenation
  - Pads prophylactically
- Meds
  - Aspirin 162-324mg chewable
  - DAPT – clopidogrel or ticagrelor
  - Heparin – 60mg/kg bolus, 12mg/kg gtt
  - Nitro – avoid in RV MI (inferior ST → R-sided EKG)
- Activate Cath Lab ASAP
- VT/VF cardiac arrest
  - 200J unsynchronized defibrillation
  - Epinephrine 1mg q3-5minutes
  - Amiodarone 300mg IV push
    - Gtt – 1mg/min for first 6 hours, then 0.5mg/min for 18
  - Consider therapeutic hypothermia

Massive PE

- ABCs
  - Oxygenation
    - Avoid positive pressure if possible – increased pulmonary hypertension
  - Circulation – inotropic support, systemic vasoconstriction, minimize PVR
    - Epinephrine – inotropy, SVR
    - Norepinephrine – SVR, inotropy
    - Dobutamine – inotropy, S&P vasodilation
    - Vasopressin – increase SVR without PVR
    - Phenylephrine – avoid; increases PVR

Appendix N. Debrief Teaching Pearls Handout

- T-PA
  - Remember checklist
  - Access before t-PA if possible
  - PERT team – thrombectomy, catheter

Wide Complex Tachycardia

- Unstable – hypotension, chest pain, AMS
  - Immediate cardioversion
- Get old EKG if possible!
- Electricity
  - Pulse – synchronized cardioversion > 100J
  - Pulseless – defibrillation 200J, ACLS
  - For cardioversion, consider premedication – propofol, benzo/opioid, etomidate
- Medications
  - Procainamide – most effective
    - Type I antidysrhythmic
    - 20-50mg/min, max 15-17mg/kg
      - Followed by gtt 1-4mg/min
    - Avoid in HFrEF, structural abnormality
    - Watch for QRS prolongation, hypotension
  - Amiodarone – 2nd most effective
    - Antidysrhythmic with mixed properties
    - Easiest to use
    - 150mg push (300mg pulseless)
    - Gtt – 1mg/kg 6 hrs, 0.5mg/kg 18 hrs
    - Hypotension
  - Lidocaine – Least effective
    - 1-1.5mg/kg IV push (~100mg)
      - Followed by gtt 1-4mg/min
    - Fewest side effects

Flash Pulmonary Edema

- Pathophysiology
  - Acute catecholamine surge
  - Elevated arterial pressure and LA pressure
  - Acute pulmonary fluid overload
    - Total body fluid overload? Maybe
- ABCs
  - Oxygenation – positive pressure
    - Non-invasive – CPAP/BiPAP
      - Clear out alveoli, O2 exchange
    - Intubation – refractory hypoxia, unable to tolerate NPPV, AMS
  - Circulation – Preload/afterload reduction
    - Acutely reduce BP
    - Nitro – SL, gtt (at least 100mcg/min)
    - ACE-I
- Other considerations
  - Ultrasound – bedside diagnosis in < 60sec
  - Lasix – not acutely indicated; consider subacutely if volume overloaded
  - HD patients
    - Remember potassium!
      - EKG early
      - Stabilize - calcium
      - Shift – insulin, albuterol, bicarbonate gtt
      - Eliminate – Lasix, kayexalate, lokelma
    - Call renal early for HD
