# Supplementary material for: Resuscitation Leadership Training: A Simulation Curriculum for Emergency Medicine Residents
Source: MedEdPORTAL. 2022 Oct 11;18:11278. doi: 10.15766/mep_2374-8265.11278 (PMC9550795; doi:10.15766/mep_2374-8265.11278)
Supplement: Supplementary file 1 — Sim Case - STEMI and VFib Arrest.docxCase Media and Labs - STEMI and VFib Arrest.pptxSim Case - Massive Pulmonary Embolism.docxCase Media and Labs - Massive PE.pptxSim Case - Wide Complex Tachycardia.docxCase Media and Labs - WCT.pptxSim Case - Missed Dialysis.docxCase Media and Labs - Missed Dialysis.pptxCAC - STEMI and VFib Arrest.docxCAC - Massive Pulmonary Embolism.docxCAC - Wide Complex Tachycardia.docxCAC - Missed Dialysis.docxCRM Presentation.pptxDebrief Handout.pdfSelect ACGME EM Milestones List.pptxOttawa GRS.docxResident Survey.docx [file mep_2374-8265.11278-s001.zip › O. Select ACGME EM Milestones List.pptx]

## Slide 1
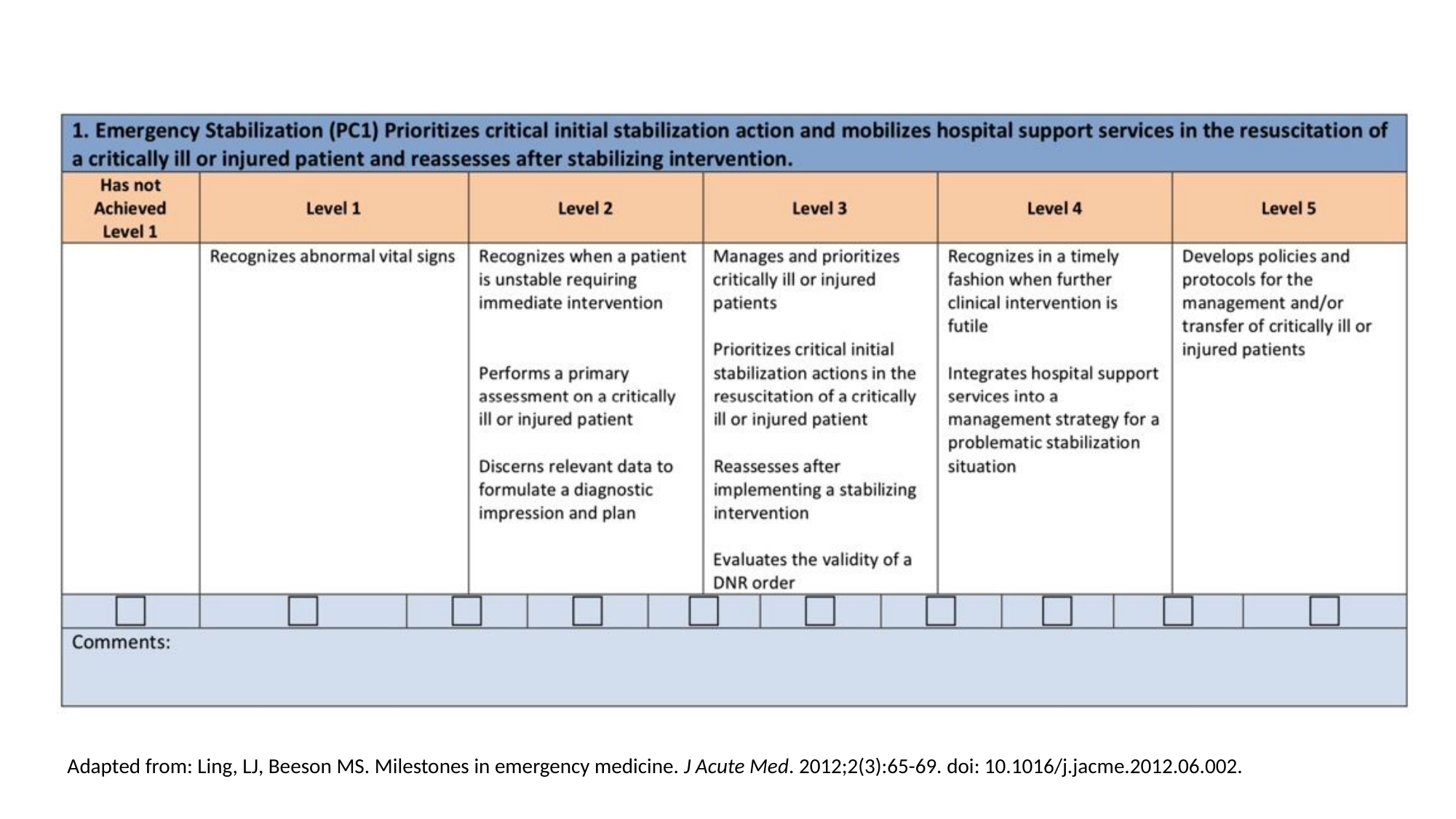

Adapted from: Ling, LJ, Beeson MS. Milestones in emergency medicine. J Acute Med. 2012;2(3):65-69. doi: 10.1016/j.jacme.2012.06.002.

## Slide 2
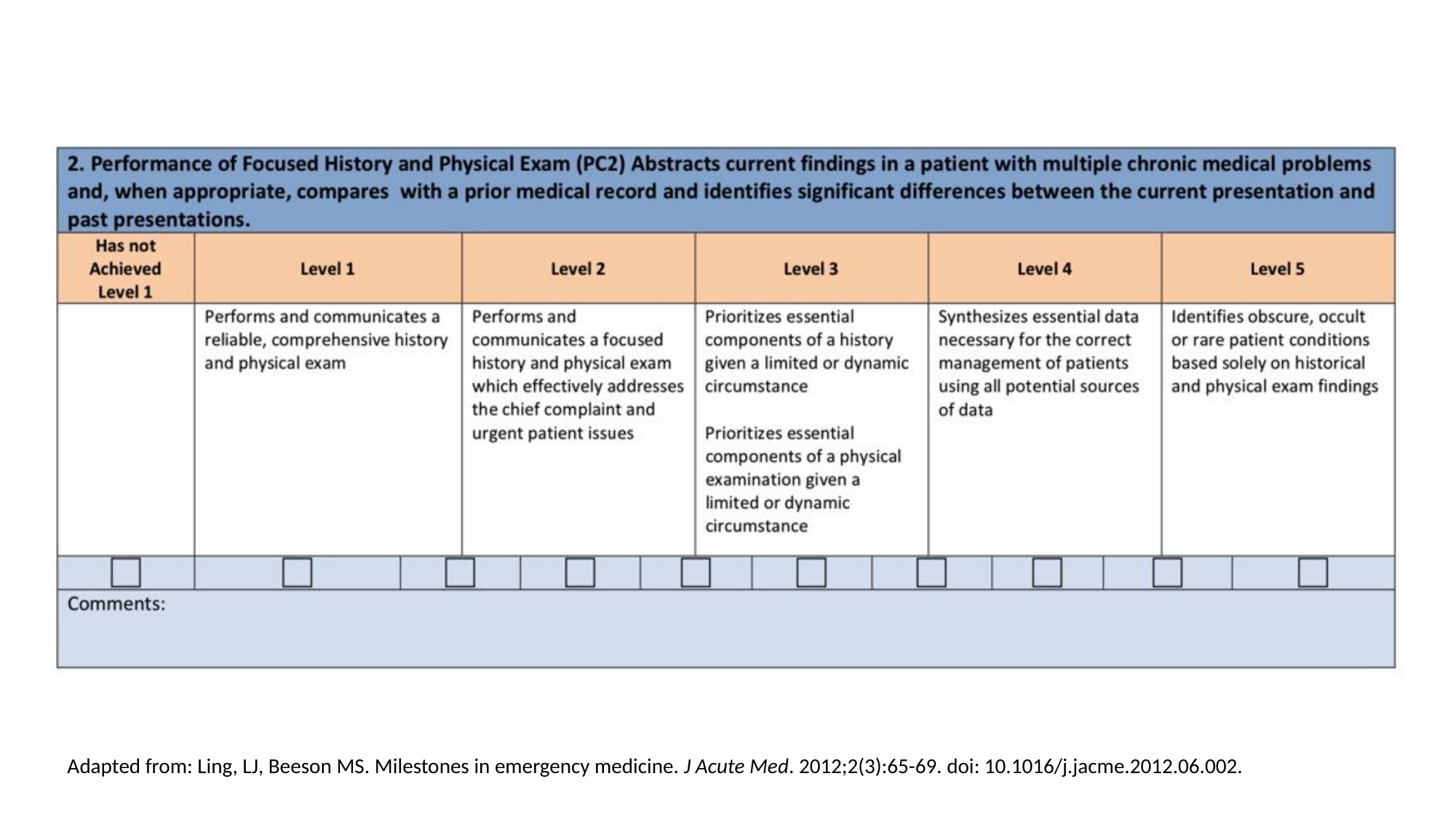

Adapted from: Ling, LJ, Beeson MS. Milestones in emergency medicine. J Acute Med. 2012;2(3):65-69. doi: 10.1016/j.jacme.2012.06.002.

## Slide 3
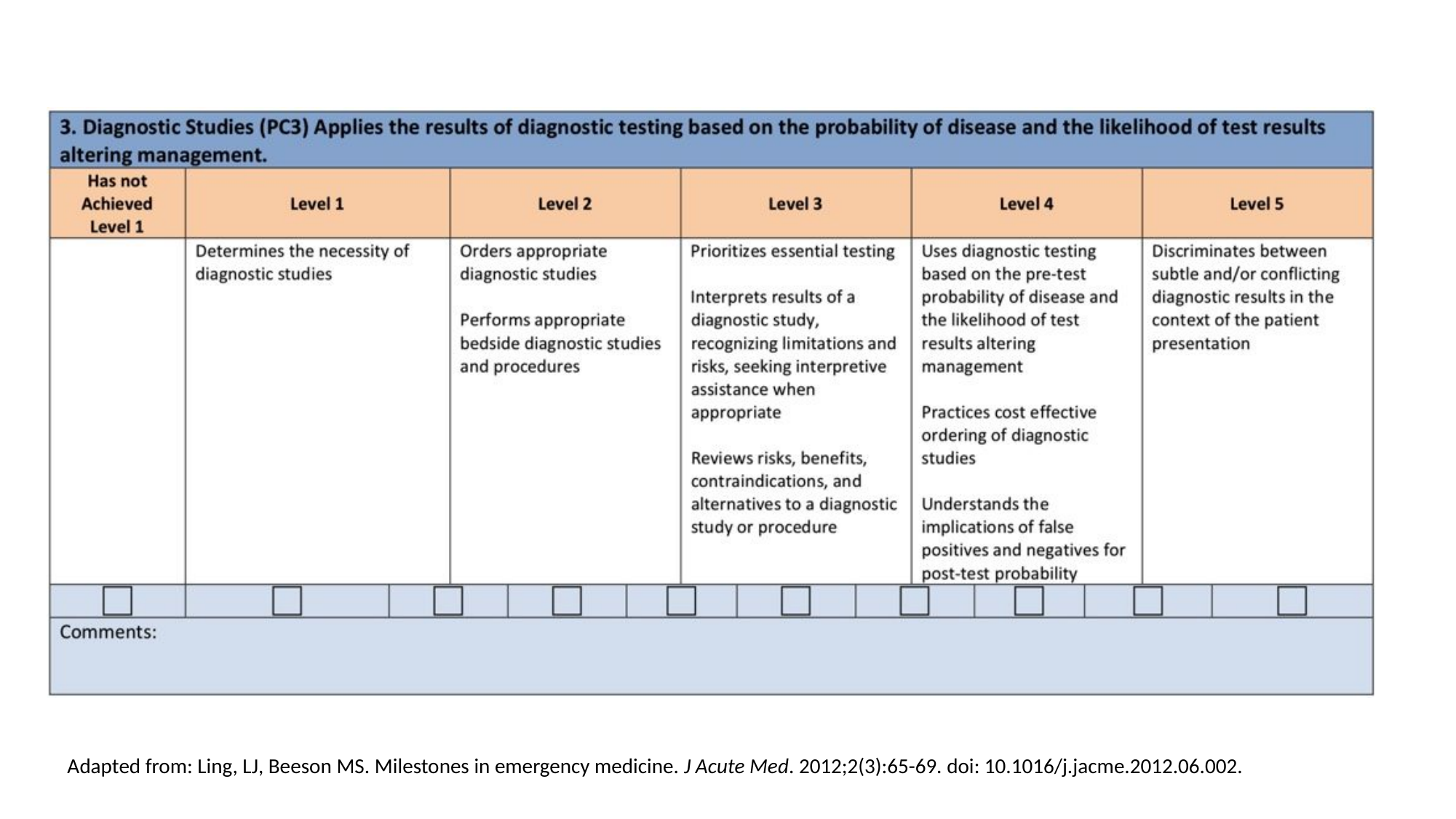

Adapted from: Ling, LJ, Beeson MS. Milestones in emergency medicine. J Acute Med. 2012;2(3):65-69. doi: 10.1016/j.jacme.2012.06.002.

## Slide 4
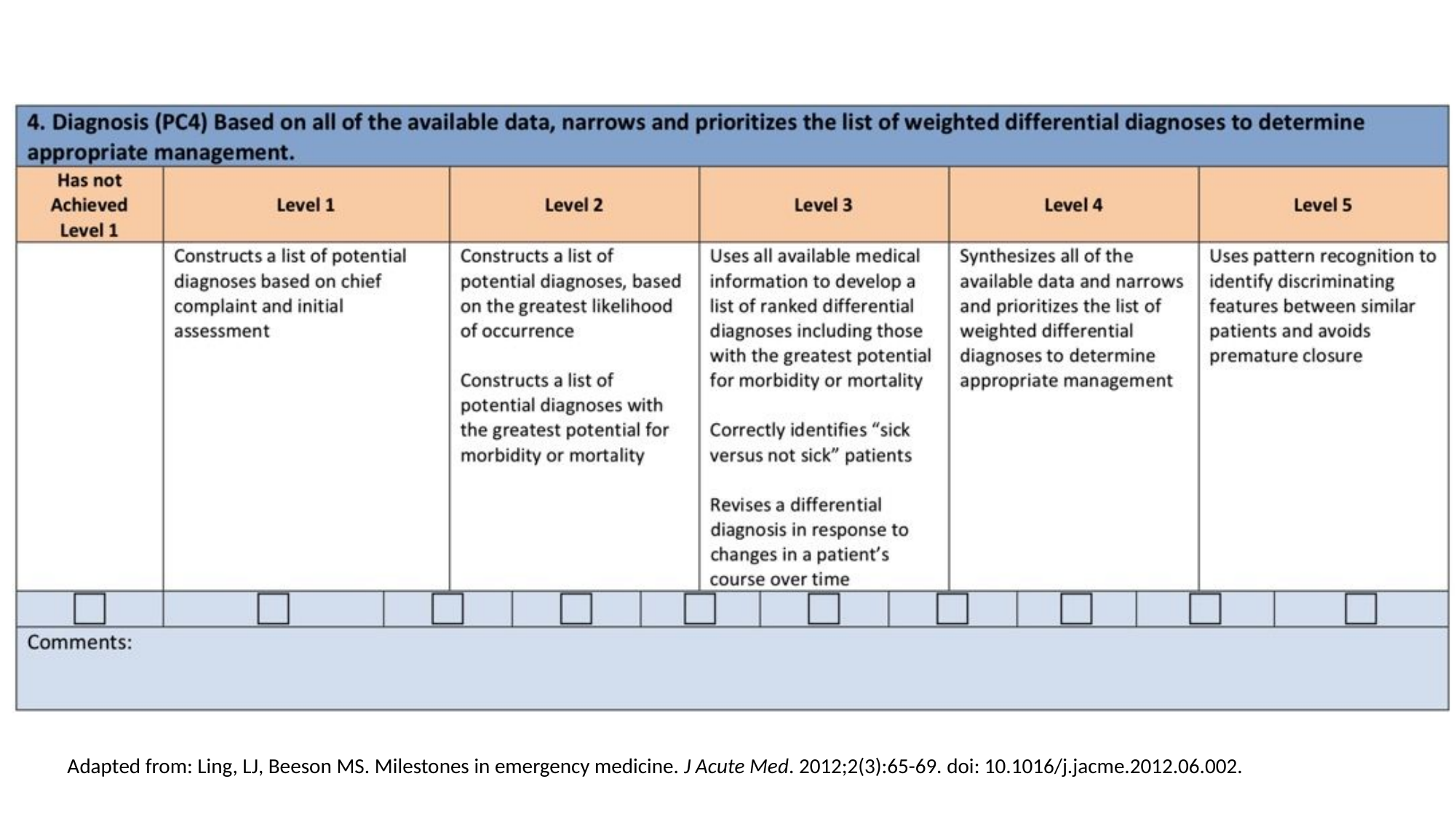

Adapted from: Ling, LJ, Beeson MS. Milestones in emergency medicine. J Acute Med. 2012;2(3):65-69. doi: 10.1016/j.jacme.2012.06.002.

## Slide 5
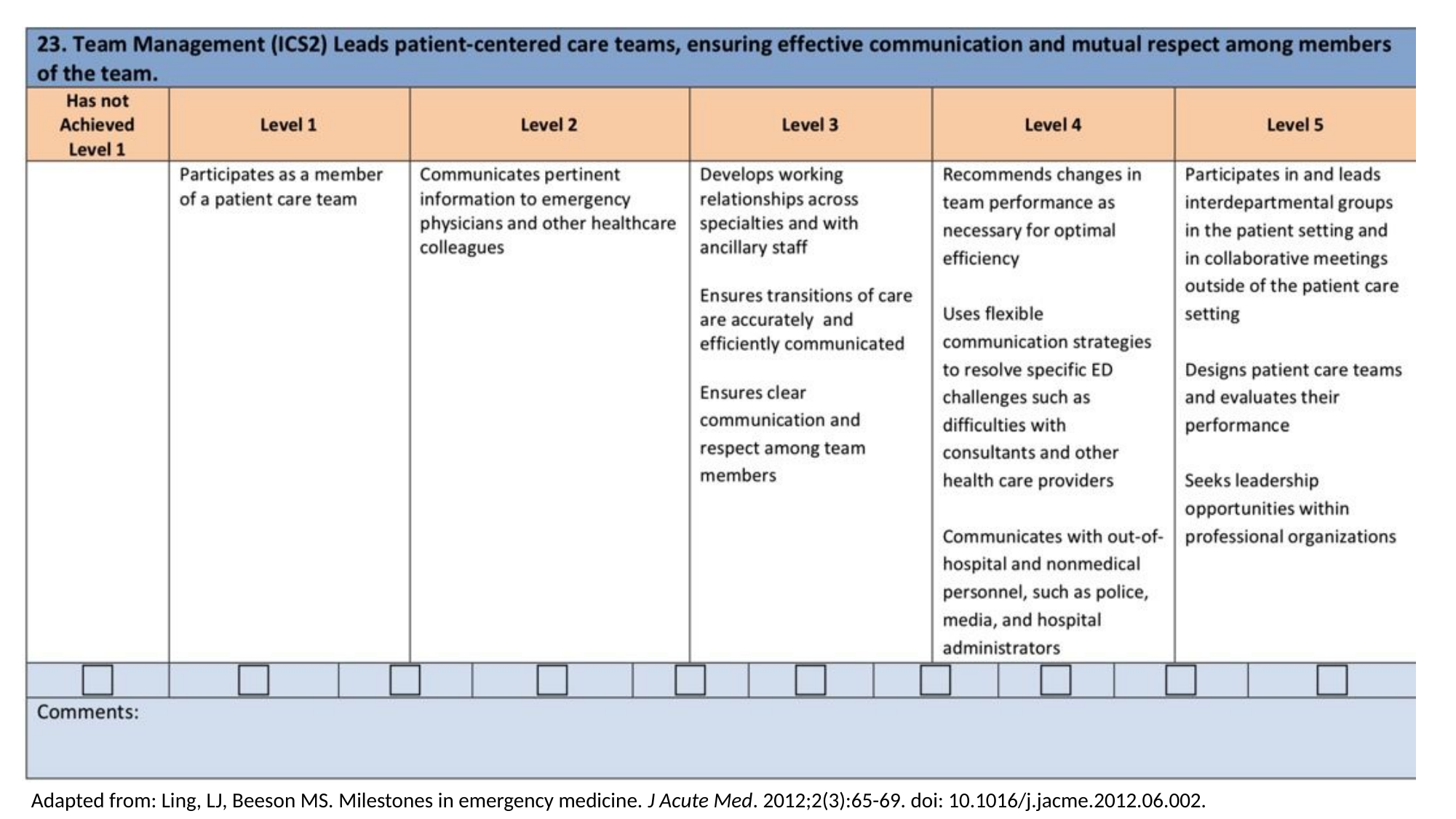

Adapted from: Ling, LJ, Beeson MS. Milestones in emergency medicine. J Acute Med. 2012;2(3):65-69. doi: 10.1016/j.jacme.2012.06.002.
