# Supplementary material for: Resuscitation Leadership Training: A Simulation Curriculum for Emergency Medicine Residents
Source: MedEdPORTAL. 2022 Oct 11;18:11278. doi: 10.15766/mep_2374-8265.11278 (PMC9550795; doi:10.15766/mep_2374-8265.11278)
Supplement: Supplementary file 1 — Sim Case - STEMI and VFib Arrest.docxCase Media and Labs - STEMI and VFib Arrest.pptxSim Case - Massive Pulmonary Embolism.docxCase Media and Labs - Massive PE.pptxSim Case - Wide Complex Tachycardia.docxCase Media and Labs - WCT.pptxSim Case - Missed Dialysis.docxCase Media and Labs - Missed Dialysis.pptxCAC - STEMI and VFib Arrest.docxCAC - Massive Pulmonary Embolism.docxCAC - Wide Complex Tachycardia.docxCAC - Missed Dialysis.docxCRM Presentation.pptxDebrief Handout.pdfSelect ACGME EM Milestones List.pptxOttawa GRS.docxResident Survey.docx [file mep_2374-8265.11278-s001.zip › Q. Resident Survey.docx]

1. Prior to this session of Resuscitation Leadership Training, I was ready to lead a resuscitation.

Not at all ready 1 2 3 **4** 5 6 7 Very Ready

1. After finishing this session of the RLT, I am ready to lead a resuscitation

Not at all ready 1 2 3 **4** 5 6 7 Very Ready

1. Prior to this session of the RLT, I was an effective communicator during resuscitations.

Not at all Effective 1 2 3 **4** 5 6 7 Very Effective

1. After finishing this session of the RLT, I will be an effective communicator during resuscitations.

Not at all Effective 1 2 3 **4** 5 6 7 Very Effective

1. My comfort managing STEMI associated V-fib arrest:

Before: Not Comfortable 1 2 3 **4** 5 6 7 Comfortable

After: Not Comfortable 1 2 3 **4** 5 6 7 Comfortable

1. My comfort managing Massive pulmonary embolism (PE):

Before: Not Comfortable 1 2 3 **4** 5 6 7 Comfortable

After: Not Comfortable 1 2 3 **4** 5 6 7 Comfortable

1. My comfort managing flash pulmonary edema:

Before: Not Comfortable 1 2 3 **4** 5 6 7 Comfortable

After: Not Comfortable 1 2 3 **4** 5 6 7 Comfortable

1. My comfort managing wide complex tachycardias:

Before: Not Comfortable 1 2 3 **4** 5 6 7 Comfortable

After: Not Comfortable 1 2 3 **4** 5 6 7 Comfortable

1. Please list 3 words you would use to describe RLT:
2. Please give any feedback on RLT’s effectiveness, what aspects are effective, and which could be improved (feel free to use the back of the page!):
